# Lignans from Tujia Ethnomedicine Heilaohu: Chemical Characterization and Evaluation of Their Cytotoxicity and Antioxidant Activities

**DOI:** 10.3390/molecules23092147

**Published:** 2018-08-27

**Authors:** Yongbei Liu, Yupei Yang, Shumaila Tasneem, Nusrat Hussain, Muhammad Daniyal, Hanwen Yuan, Qingling Xie, Bin Liu, Jing Sun, Yuqing Jian, Bin Li, Shenghuang Chen, Wei Wang

**Affiliations:** 1 TCM and Ethnomedicine Innovation & Development International Laboratory, Innovative Drug Research Institute, School of Pharmacy, Hunan University of Chinese Medicine, Changsha, 410208, China; ybliu2018@163.com (Y.L.); yangyupei24@163.com (Y.Y.); tasneemshum@gmail.com (S.T.); nusrat_hussain42@yahoo.com (N.H.) daniyaldani151@yahoo.com (M.D.); hanwyuan@hotmail.com (H.Y.); XieQL1992@163.com (Q.X.); cpujyq2010@163.com (Y.J.); libin_hucm@hotmail.com (B.L.); cshtyh@163.com (S.C.); 2 H.E.J. Research Institute of Chemistry, International Center for Chemical and Biological Sciences, University of Karachi, Karachi-75270, Pakistan; 3 Hunan Province Key Laboratory of Plant Functional Genomics and Developmental Regulation, College of Biology, Hunan University, Changsha 410082, China; binliu2001@hotmail.com; 4 Shaanxi Key Laboratory of Basic and New herbal Medicament Research, Shaanxi Collaborative Innovation Center of Chinese Medicinal Resource Industrialization, Shaanxi University of Chinese Medicine, Xianyang 712046, China; ph.175@163.com

**Keywords:** lignans, heilaohu, tujia ethnomedicine, chemical characterization, cytotoxicity, antioxidant

## Abstract

Heilaohu, the roots of *Kadsura coccinea,* has a long history of use in Tujia ethnomedicine for the treatment of rheumatoid arthritis and gastroenteric disorders, and a lot of work has been done in order to know the material basis of its pharmacological activities. The chemical investigation led to the isolation and characterization of three new (**1**–**3**) and twenty known (**4**–**23**) lignans. Three new heilaohulignans A-C (**1**–**3**) and seventeen known (**4**–**20**) lignans possessed dibenzocyclooctadiene skeletons. Similarly, one was a diarylbutane (**21**) and two were spirobenzofuranoid dibenzocyclooctadiene (**22**–**23**) lignans. Among the known compounds, **4**–**5**, **7**, **13**–**15** and **17**–**22** were isolated from this species for the first time. The structures were established, using IR, UV, MS and NMR data. The absolute configurations of the new compounds were determined by circular dichroism (CD) spectra. The isolated lignans were further evaluated for their cytotoxicity and antioxidant activities. Compound **3** demonstrated strong cytotoxic activity with an IC_50_ value of 9.92 µM, compounds **9** and **13** revealed weak cytotoxicity with IC_50_ values of 21.72 µM and 18.72 µM, respectively in the HepG-2 human liver cancer cell line. Compound **3** also showed weak cytotoxicity against the BGC-823 human gastric cancer cell line and the HCT-116 human colon cancer cell line with IC_50_ values of 16.75 µM and 16.59 µM, respectively. A chemiluminescence assay for antioxidant status of isolated compounds implied compounds **11** and **20,** which showed weak activity with IC_50_ values of 25.56 µM and 21.20 µM, respectively.

## 1. Introduction

*Kadsura coccinea* (Lem.) A. C. Smith belongs to the medicinally important genus *Kadsura* from the Schisandraceae family. It is an evergreen climbing shrub, which is mainly distributed in south-western provinces of P. R. China [1]. Its leaves, fruits, stems and roots are used as medicine. The fruits have unique shapes and high nutritional and medicinal values [2]. The stems and roots are called Heilaohu in Tujia ethnomedicine for looking swarthy while dispelling wind effectively. The isolates of this plant mainly contain lignans, triterpenoids and essential oils. Bioactive lignans and triterpenoids from this plant are of special interest [3]. The compounds from genus *Kadsura* have been reported with different bioactivities including anti-tumor [4,5], anti-HIV [6,7,8], anti-inflammatory [9,10], inhibition of nitric oxide (NO) production [11,12] and other pharmacological effects.

The lignans from Heilaohu are very important due to their bioactivities and structural diversity. The lignans from this plant can be divided into four different categories on the basis of skeleton types: dibenzocyclooctadienes, spirobenzofuranoid dibenzocyclooctadienes, diarylbutanes, and aryltetralins lignans. Dibenzocyclooctadiene (two benzene rings sharing an eight membered ring neighborhood) is the most common basic skeleton in Heilaohu. Methoxy, hydroxyl and methylenedioxy are the most frequently found substituents at benzene rings, while other important substituents including acetyl-, angeloyl-, tigloyl-, propanoyl-, benzoyl-, cinnamoyl- and butyryl- groups are invariably presented at C-1, C-6 or C-9 [13,14,15]. Spirobenzofuranoid dibenzocyclooctadienes are rare in other genera and can be considered as the characteristic chemical constituents of genus *Kadsura*. This category features a furan-ring at C-14, C-15 and C-16 positions and a ketonic group at the C-1 or C-3 position [3], and the same connections on the eight membered ring located at the C-6 or C-9 position. Diarylbutanes and aryltetralins have previously been reported but are not very common in genus *Kadsura*, and most of them were found in the DCM (CHCl_3_) layer and EtOAc layer.

This work was conducted to further explore lignans from Heilaohu. The chemical investigation led to the isolation and characterization of three new (**1**–**3**) and twenty known (**4**–**23**) lignans. The three new *Kadsura* lignans A–C (**1**–**3**) and seventeen known lignans, schizandrin (**4**) [16], binankadsurin A (**5**) [17], acetylbinankadsurin A (**6**) [18], isobutyroylbinankadsurin A (**7**) [19], isovaleroylbinankadsurin A (**8**) [19], kadsuralignan I (**9**) [20], kadsuralignan J (**10**) [20], kadsuralignan L (**11**) [21], kadsulignan N (**12**) [22], longipedunin B (**13**) [15], schisantherin F (**14**) [23], schizanrin D (**15**) [23], acetylgomisin R (**16**) [24], intermedin A (**17**) [25], kadsurarin (**18**) [14], kadsutherin A (**19**) [25] and kadsuphilol A (**20**) [26] possessed dibenzocyclooctadiene skeletons. Similarly, meso-dihydroguaiaretic acid dimethyl (**21**) [27] had a diarylbutane type. Schiarianrin E (**22**) [28] and schiarisanrin A (**23**) [29] contained spirobenzofuranoid dibenzocyclooctadiene lignan skeletons.

A literature survey revealed that kadsulignan I (**9**) exhibited inhibitory effects on LPS-induced NO production in BV-2 cells with IC_50_ value of 21.00 µM [30]. Kadsuralignan L (**11**) demonstrated moderate NO production inhibitory activity with an IC_50_ value of 52.50 µM [21]. Heilaohu has been used for the treatment of rheumatoid arthritis in traditional medicine for a long time, and a few of its isolated compounds have been used for their anti-inflammatory and cytotoxic activities [3]. With the aim of searching for natural compounds which are responsible for folk efficacy and medicinal application as anti-cancer agents and as anti-inflammatory agents, we employed a chemiluminescence assay for anti-oxidant activity to find out the anti-inflammatory properties of a compound. We also used a cytotoxicity assay against cancer cell lines, namely HepG-2 human liver cancer cells, BGC-823 human gastric cancer cells and HCT-116 human colon cancer cells, after the chemical characterization of compounds.

## 2. Results and Discussion

### 2.1. Structure Characterization of the Isolated Compounds from Heilaohu

**Figure 1:**
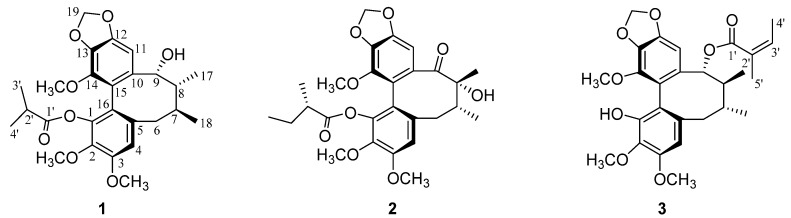
Structures of heilaohulignans A–C (**1**–**3**).

Heilaohulignan A (**1**) (Figure 1) was obtained as an amorphous powder. Its molecular formula, C_26_H_32_O_8_, was determined by [M + Na]^+^ ion peak at *m*/*z* 495.1998 (calcd. 495.1995) in HR-ESI-MS, showing 11 degrees of unsaturation. The UV data, with absorption maxima at *λ*_max_ 242 nm, and its IR spectrum, with absorption bands at 3419 (OH), 1645 (C=C) and 1463 cm^−1^ (aromatic moiety), suggested that **1** is a dibenzocyclooctadiene lignan with a hydroxyl substitution.

The ^1^H- and ^13^C-NMR spectra of **1** (Table 1) indicated the presence of 12 aromatic carbons (*δ*_C_ 141.7 (C-1), 138.8 (C-2), 151.5 (C-3), 113.0 (C-4), 134.8 (C-5), 102.4 (C-11), 148.8 (C-12), 135.0 (C-13), 140.3 (C-14), 118.2 (C-15) and 122.9 (C-16)) and two aromatic proton singlets at *δ*_H_ 6.71 (1H, s) and 6.32 (1H, s), which were assignable to H-4 and H-11, respectively. A butane chain was deduced on the cross-peaks of H-6 (*δ*_H_ 2.65, m), H-7 (*δ*_H_ 2.01, m), H-8 (*δ*_H_ 1.81, m) and H-9 (*δ*_H_ 4.73, s) in the ^1^H-^1^H COSY spectrum. In addition, in the HMBC spectrum, correlations were found between H-9 and C-10, C-8 and C-15, and between H-6 and C-4, C-7 and C-16. The functional moieties evident from the ^1^H- and ^13^C-NMR data included one methylenedioxy, three methoxy groups and four methyl groups; the presence of signals at *δ*_H_ 0.97 (d, *J* = 7.0 Hz, 3H), 1.09 (d, *J* = 7.0 Hz, 3H) and 2.61 (m, 1H), and *δ*_C_ 176.7 (C=O), 18.7 (CH_3_), 18.7 (CH_3_), 34.0 (CH) suggested the presence of an isobutyroyl group.

**Figure 2:**
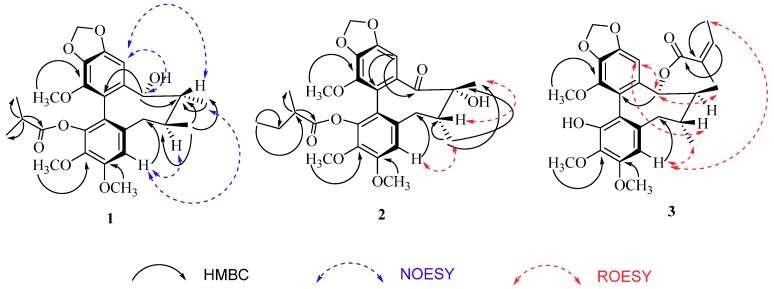
Key HMBC and NOESY correlations of heilaohulignan A (**1**) and ROESY correlations of heilaohulignans B–C (**2**–**3**).

Further analysis of the HMBC spectrum (Figure 2) showed three methoxy groups (*δ*_H_ 3.96, 3.78, 3.89, 2-OCH_3_, 3-OCH_3_ and 14-OCH_3_, respectively), with two secondary methyl groups (*δ*_C_ 15.3 and 20.0) assignable to CH_3_-17 and CH_3_-18, respectively, and one methylenedioxy group (*δ*_H_ 6.07, 6.02, each 1H, d, *J* = 1.5 Hz) located between C-12 and C-13. The NMR data was similar to a known compound, binankadsurin A (**5**) [17]. However, different carbon and proton chemical shifts for C-1′, C-2′, C-3′ and C-4′ indicated that the methyl group located at C-1 was substituted by an isobutyroyl group. Thus, the planar structure of compound **1** was established.

The biphenyl group in **1** was found to have a twisted boat/chair configuration from its CD spectrum (Figure S63), which showed a negative Cotton effect around 250 nm and a positive value around 220 nm, favoring the *S*-biphenyl configuration as gomisin F [31,32] suggesting **1** possesses an *S*-biphenyl configuration [28]. The observed NOESY correlations (Figure 2) of *δ*_H_ 6.71 (H-4) and *δ*_H_ 2.01 (H-7), *δ*_H_ 1.01 (H_3_-17), *δ*_H_ 6.32 (H-11) and *δ*_H_ 1.81 (H-8), *δ*_H_ 4.73 (H-9), indicated that CH_3_-17 was *α*-oriented, and CH_3_-18 and H-9 as *β*-oriented. Hence, 7*S*, 8*R*, and 9*R* configurations were confirmed at C-7, C-8, and C-9, respectively. Based on these data, the structure of **1** was unambiguously determined and was named as heilaohulignan A.

Heilaohulignan B (**2**) (Figure. 1) was obtained as an amorphous powder. Its molecular formula C_27_H_32_O_9_ was determined by [M + COOH]^−^ ion peak at *m*/*z* 545.2028 (calcd. 545.2026) in HR-ESI-MS. The UV absorption bands at 241 nm and IR absorption bands at 3446 (OH), 1704 (C=O) and 1457, 1579 cm^−1^ (aromatic ring) suggested **2** as a dibenzocyclooctadiene lignan possessing a hydroxy group and an ester.

The ^1^H- and ^13^C-NMR spectra of **2** (Table 1) supported a dibenzocyclooctadiene lignan basic skeleton with one methylenedioxy, three methoxy, and a 2-methylbutyryloxy (O-isovaleryl) substituents. The ^1^H-NMR signals at *δ*_H_ 2.43 (m, H-2′), 1.40 (m, H-3′), 1.62 (m, H-3′), 0.86 (t, *J* = 7.4, H-4′), 1.02 (d, *J* = 7.0, H-5′) and ^13^C-NMR signals at *δ*_C_ 173.1 (C-1), 41.5 (C-2′), 26.8 (C-3′), 11.7 (C-4′), and 16.9 (C-5′) were assignable to a 2-methylbutyryloxy group. Comparison of the NMR data of **2** with a known lignan, kadoblongifolins A, showed great similarity [14]. The only difference was the presence of a 2-methylbutyryloxy (*O*-isovaleryl) group at C-1 in **2**.

The HMBC correlations (Figure 2) of methylenedioxy hydrogens (*δ*_H_ 5.96, 1H, s, OCH_2_O-19a, 6.02, 1H, s, OCH_2_O-19b) with carbons at *δ*_C_ 138.8 (C-12) and 151.5 (C-13) were used to locate its attachment to C-12 and C-13. The methoxy groups were located at C-2, C-3, and C-14, with one secondary methyl group (*δ*_C_ 14.8) assignable to CH_3_-18 and one quaternary methyl group (*δ*_C_ 23.3) assignable to CH_3_-17. The keto group position was confirmed at C-9 by HMBC correlations of H-11 (*δ*_H_ 6.52) and H_3_-17 (*δ*_H_ 1.33) with C-9 (*δ*_C_ 207.3).

The CD curve of **2** (Figure S64) showed a positive Cotton effect around 205 nm and a negative Cotton effect around 254 nm, favoring the *S*-biphenyl configuration as gomisin F [31,32]. The ROESY (Rotating Frame Overhauser Effect) spectrum (Figure 2) of **2** showed cross-correlation peaks between *δ*_H_ 6.58 (H-4) and *δ*_H_ 0.89 (CH_3_-18); *δ*_H_ 2.04 (H-7) and *δ*_H_ 1.33 (CH_3_-17), which confirmed that CH_3_-17 was *β*-oriented and CH_3_-18 was *α*-oriented, thus supporting 7*R* and 8*R* configurations. The compound 2-methylbutyryl is derived from 2-methylbutyryl-CoA biosynthetically, in which the stereochemistry of 2-methyl group is *S*. As stereochemistry is retained, the configuration in the 2-methylbutyryl group was shown as *S*. Based on these spectral data, the structure of **2** was deduced and named as heilaohulignan B.

Heilaohulignan C (**3**) (Figure 1) was obtained as a yellow oil. Its molecular formula, C_27_H_32_O_8_, was determined by [M + Na]^+^ ion at *m*/*z* 507.1990 (calcd. 507.1995) in HR-ESI-MS, suggesting 12 degrees of unsaturation. The UV data, with absorption maxima at *λ*_max_ 242 nm, and its IR spectrum, with absorption bands at 3417 (-OH), 1700 (C=O) and 1613, 1503 cm^−1^ (aromatic moiety), suggested **3** as a dibenzocyclooctadiene lignan with a hydroxyl substitution.

The ^1^H- and ^13^C-NMR spectra of **3** (Table 1) indicated the presence of 12 aromatic carbons, two aromatic protons, one methylenedioxy and three methoxy groups, suggesting the presence of a biphenyl moiety. A butane chain was deduced on the cross-peaks of H-6 (*δ*_H_ 2.66, m), H-7 (*δ*_H_ 2.12, m), H-8 (*δ*_H_ 2.10, m) and H-9 (*δ*_H_ 5.62, s) in the ^1^H-^1^H COSY spectrum. In the HMBC spectrum (Figure 2.), two methyl groups (CH_3_-17, CH_3_-18) exhibited correlations with C-8 and C-9, and three methoxy groups at *δ*_H_ 3.84, 3.84 and 3.90 (2-OCH_3_, 3-OCH_3_ and 14-OCH_3_) showed correlations with C-2, C-3, and C-14, respectively, confirming these substituted groups of positions undoubtedly. Thus, the planar structure of compound **3** was the same as angloybinankadsurin A [15]. However, the chemical shifts of C-4′ and C-5′ of **3** were around 4–5 ppm different from the known, which led to doubt about the stereochemistry of **3**.

The biphenyl group in **3** was determined to have an *S*-biphenyl configuration from its CD spectrum (Figure S65), identical to that of **1** and **2**. However, the ROESY experiment (Figure 2) revealed that cross-correlation peaks between *δ*_H_ 6.41 (H-4) and *δ*_H_ 0.97 (H_3_-5′); *δ*_H_ 6.54 (H-11) and *δ*_H_ 2.12 (H-7), *δ*_H_ 5.62 (H-9); *δ*_H_ 5.62 (H-9) and *δ*_H_ 1.09 (H_3_-17), *δ*_H_ 2.12 (H-7); *δ*_H_ 1.61 (H_3_-18) and *δ*_H_ 1.47 (H_3_-4′) confirmed that CH_3_-17 was *β*-oriented and CH_3_-18 was *α*-oriented, which were essentially different from the known angloybinankadsurin A [15], where CH_3_-17 and CH_3_-18 are both *α*-oriented. Thus *R*, *S*, and *R* configurations were confirmed at C-7, C-8, and C-9, respectively. The ROESY correlation peaks between *δ*_H_ 6.02 (H-3′) and *δ*_H_ 0.97 (H_3_-5′), and comparison of data in the literature supported *Z*-configuration for the double bond in the angeloyloxy moiety. Based on these spectral data, the complete structure of **3** was established and it was named as heilaohulignan C.

The spectroscopic data of known compounds (Figures S20–S59) were in good agreement with those reported in the literature. Thus, the known compounds were identified as schizandrin (**4**), binankadsurin A (**5**), acetylbinankadsurin A (**6**), isobutyroylbinankadsurin A (**7**), isovaleroybinankadsurin A (**8**), kadsuralignan I (**9**), kadsuralignan J (**10**), kadsuralignan L (**11**), kadsulignan N (**12**), longipedunin B (**13**), schisantherin F (**14**), schizanrin D (**15**), acetylgomisin R (**16**), intermedin A (**17**), kadsurarin (**18**), kadsutherin A (**19**), kadsuphilol A (**20**), meso-dihydroguaiaretic acid dimethyl ether (**21**), schiarianrin E (**22**), and schiarisanrin A (**23**) (Figure S1).

For the chemical characterization of dibenzocyclooctadienes, there was little to distinguish among different compounds whether the substituents linked to C-1 or C-6/C-9. When the substituents such as acetyl-, angeloyl-, tigloyl-, propanoyl-, benzoyl-, cinnamoyl- and butyryl- groups connected to C-6/C-9, *δ*_H__-6/9_ was displayed over 5.5 ppm and the relationship with C-1′ could be found in HMBC, while *δ*_H-6/9_ would be revealed around 4.7 ppm if substituents attached to C-1. For spirobenzofuranoid dibenzocyclooctadienes, *δ*_C-1/3_ with a ketonic group at 195 ppm nearby and *δ*_C-16_ around 65 ppm could be classified in ^13^C-NMR. In addition, *δ*_C-20_ around 78 ppm (CH_2_) is a typical signal in this compound.

### 2.2. Cytotoxic Activity of Isolated Compounds

Compounds **1**–**23** were assayed for their cytotoxic activity against the HepG-2 human liver cancer cell line, the BGC-823 human gastric cancer cell line and the HCT-116 human colon cancer cell line. The results are summarized in Table 2: heilaohulignan C (**3**) showed good cytotoxicity in HepG-2 human liver cancer cells with IC_50_ values of 9.92 µM, and weak cytotoxicity against BGC-823 human gastric cancer cells and HCT-116 human colon cancer cells with IC_50_ values of 16.75 µM and 16.59 µM, respectively. Meanwhile, in the HepG-2 human liver cancer cell line, kadsuralignan I (**9**) and longipedunin B (**13**) revealed weak cytotoxicity with IC_50_ values of 21.72 µM and 18.72 µM, respectively. The remaining compounds showed no cytotoxicity against the three cancer cell lines. Compounds **3**, **9** and **20** demonstrated good activity against all cells. Compounds **1**–**20**, bearing the same dibenzocyclooctadiene skeleton, indicate that spatial configuration and the relative configuration of structures may have an impact on bioactivities.

### 2.3. Antioxidant Activity of Isolated Compounds

Compounds **1**–**23** were assayed for their antioxidant activity using a chemiluminescence assay. As shown in Table 3, kadsuralignan L (**11**) showed weak activity with an IC_50_ value of 25.56 µM, and kadsuphilol A (**20**) with an IC_50_ value of 21.20 µM. The remaining compounds exhibited no antioxidant activity.

## 3. Materials and methods

### 3.1. Plant Material

Heilaohu were collected from Huaihua City of Hunan Province, China. The plant was identified by Wei Wang. It has been deposited at Sino-Pakistan TCM (Traditional Chinese Medicine) and the Ethnomedicine Research Center, School of Pharmacy, Hunan University of Chinese Medicine, Changsha, China.

### 3.2. General and Solvents

The HR-ESI-MS spectra were performed on Waters UHPLC-H-CLASS/XEVO G2-XS Qtof, Waters Corporation, Milford, MA, USA. NMR data were recorded on Bruker AV-600 spectrometers (Bruker Technology Co., Ltd., Karlsruhe, Germany) with TMS (Tetramethylsilane) as an internal standard. Column chromatographic silica gel (80–100 mesh, 200–300 mesh and 300–400 mesh) was purchased from Qingdao Marine Chemical Inc., Qingdao, China. Semipreparative HPLC was performed on an Agilent 1100 liquid chromatograph (Agilent Technologies, Santa Clara, CA, USA) with an Agilent C_18_ (34 mm × 25 cm) column. Fractions were monitored by TLC, and spots were visualized by heating silica gel plates sprayed with 5% H_2_SO_4_ in vanillin solution. Petroleum ether (PE), hexane, ethyl acetate (EtOAc), ethanol, n-butanol (n-BuOH), methanol (MeOH) and dichloromethane (CH_2_Cl_2_) were purchased from Shanghai Titan Scientific Co., Ltd, Shanghai, China. Acetonitrile (HPLC grade) and methanol (HPLC grade) were from Merck KGaA, 64271 Darmstadt, Germany.

### 3.3. Experimental Procedures

Heilaohu (200 kg) was extracted twice with 80% ethanol for 2 h under reflux extraction. All extract solvents were evaporated under vacuum to obtain a crude extract (6 kg). Half of the extracts (3 kg) were suspended in water and partitioned with PE, CH_2_Cl_2_, EtOAc and n-BuOH, respectively.

The CH_2_Cl_2_ layer (945 g) was crudely separated on a silica gel column (6 kg, 25 cm × 75 cm) using gradient elution with cyclohexane/ethyl acetate/methanol (80:1:0, 20:1:0, 10:1:0, 5:1:0, 1:1:0, 0:1:0, 0:0:1, *v*:*v*) to afford twelve fractions. Fraction 5 (49.5 g) was subjected to a silica gel column (8 cm × 45 cm, 800 g), and eluted with cyclohexane/CH_2_Cl_2_ /EA (1:0:0, 80:1:0, 40:1:0, 20:1:0, 10:1:0, 5:1:0, 3:1:0, 2:1:0, 1:1:0, 0:1:0, 0:40:1, 0:20:1, 0:10:1, 0:5:1, *v*:*v*:*v*) to obtain twelve sub-fractions (E1–E12). Sub-fraction E6 (2.0 g) was repeated purified by a silica gel column (3 cm × 60cm, 40 g) eluted with hexane/CHCl_3_/acetone (10:20:1, 20:10:1, 20:20:1, 40:10:1, *v*:*v*:*v*) to yield **2** (3 mg). Fraction 8 (40 g) was chromatographed by column chromatography on a silica gel (5 cm× 80 cm, 400 g) using the gradient system (CH_2_Cl_2_/methanol, 40:1, 20:1, 10:1, 5:1, 3:1, 1:1, 0:1, *v*:*v*) to afford ten fractions (H1–H10). Sub-fraction H7 (PE/CHCl_3_/methanol, 80:1:0, 15.0 g) was repeat purified by a silica gel column (4 cm × 45cm, 100 g) eluted with PE/CHCl_3_/methanol (40:1:0, 20:1:0, 10:1:0, 5:1:0, 3:1:0, 2:1:0, 1:1:0, 0:1:0, 0:40:1, 0:20:1, 0:10:1, 0:0:1, *v*:*v*:*v*) to afford **6** (800 mg). Sub-fraction H8 (15.0 g) was repeat purified by a silica gel column (4cm × 60 cm, 450 g) eluted with PE/acetone (40:1, 20:1, 10:1, 5:1, 3:1, 2:1:0, 1:1, *v*:*v*) to yield **5** (300 mg). Fraction 9 (53.9 g) was chromatographed by column chromatography on a silica gel (7cm× 60 cm, 500 g) using the gradient system (PE/EA,10:1, 5:1, 3:1, 2:1, 1:1, 0:1, *v*:*v*) to afford fourteen fractions (I1–I14). Sub-fraction I10 (0.5 g) was repeated purified by an RP-18 column eluted with methanol/water (40%, 50%, 60%, 70%, 80%, 90%, 100%) to yield **4** (5 mg). Sub-fraction I10-4 was purified by semi preparative HPLC with 73% MeOH-H_2_O to obtain **17** (5 mg, t_R_ = 20.6 min). Sub-fraction I10-6 was purified by semi preparative HPLC with 71% MeOH-H_2_O to obtain **18** (15 mg, t_R_ = 41.3 min). Sub-fraction I12 was purified by semi preparative HPLC with 72% MeOH-H_2_O to yield **19** (15 mg, t_R_ = 78.1 min) and **20** (25 mg, t_R_ = 29.0 min).

The EtOAc layer (530 g) was separated into eight fractions (fraction 1–8) on a 80–100 mesh silica gel column (6.5 kg), using a step gradient elution with PE/EtOAc (10:0, 20:1, 9:1, 8:2, 7:3, 6:4, 1:1, 0:10). Fraction 3 (90 g) was applied to a silica gel column (200–300 mesh, 4.5 kg) with cyclohexane/EtOAc (10:0, 95:5, 90:1, 85:15, 8:2, 7:3, 6:4, 1:1), so as to afford 10 sub-fractions. Sub-fractions were subjected to repeated silica gel columns (isocratic elution and step gradient elution) and Sephadex LH-20 (MeOH/H_2_O = 1:1) to give compounds **1** (11.9 mg), **7** (7.7 mg) and **8** (2.1 g), and the mini-fractions were conducted to semi preparative HPLC (MeOH-H_2_O) to gain compound **3** (14.6 mg) (77% MeOH-H_2_O), **9** (28.4 mg) (80% MeOH-H_2_O), **10** (35.6 mg) (80%MeOH/H_2_O), **13** (7.3 mg) (76%MeOH-H_2_O) and **22** (9.0 mg) (80%MeOH-H_2_O). Fraction 4 (60 g) was purified by a silica gel column (300–400 mesh, 4 kg) with PE/EtOAc (10:0, 10:1, 9:1, 8:2,7:3, 6:4, 1:1) to provide 12 sub-fractions. Fraction 5 (50 g) was chromatographed on a silica gel (300–400 mesh, 3.5 kg) to obtain 12 sub-fractions. Sub-fractions from fraction 4 and 5 were fractionated under the same chromatography conditions to obtain compounds **11** (130.1 mg), **12** (29.6 mg), **14** (7.3 mg), **15** (23.2 mg), **16** (2.7 mg), **21** (1.3 mg) and **23** (4.8 mg). The solvents of recrystallization of **7**, **8** and **9** were MeOH (HPLC grade), cyclohexane and hexane, respectively.

### 3.4. Spectroscopic Data of New Compounds

Heilaohulignan A (**1**): Amorphous powder, [*α*]${}_{D}^{25}$ − 160.0 (*c* = 0.0125, CHCl_3_), UV (MeOH) *λ*_max_ (log *ε*): 242 (4.57) nm, IR (KBr) *ν*_max_ 3419, 1645, 1463, 1101, 721, 655 cm^−1^; ^1^H- and ^13^C-NMR data, Table 1; ^+^HR-ESI-MS *m*/*z* 495.1998 ([M + Na]^+^, calcd. 495.1995).

Heilaohulignan B (**2**):Amorphous powder, [*α*]${}_{D}^{25}$ + 40.0 (*c* = 0.10, CHCl_3_), UV (MeOH) *λ*_max_ (log *ε*): 241(4.58) nm, IR (KBr) *ν*_max_ 3446, 2932, 2360, 1704, 1457, 1102; ^1^H- and ^13^C-NMR data, Table 1; ^−^HR-ESI-MS *m*/*z* 545.2028 ([M + COOH]^−^, calcd. 545.2026).

Heilaohulignan C (**3**): Yellow oil, [*α*]${}_{D}^{25}$ + 48.0 (*c* = 0.09, CH_3_OH), UV (MeOH) *λ*_max_ (log *ε*): 241 (4.59) nm; IR (KBr) *ν*_max_ 3417, 2945, 1700, 1457, 1368, 1248, 1108, 1025, ^1^H- and ^13^C-NMR data, Table 1; +HR-ESI-MS *m*/*z* 507.1990 ([M + Na]^+^, calcd. 507.1995).

### 3.5. Cytotoxicity Assay

Cell viability was determined by MTT assay [33]. Taxol was used as a positive control. HepG-2 human liver cancer cells, BGC-823 human gastric cancer cells and HCT-116 human colon cancer cells were seeded at 6 × 10^3^ cells/well in 96-well plates. Cells were allowed to adhere overnight, and then the media were replaced with fresh medium containing selected concentrations of the natural compounds dissolved in DMSO. After 48 h incubation, the growth of the cells was measured. The effect on cell viability was assessed as the percent cell viability compared with the untreated control group, which were arbitrarily assigned 100% viability. The compound concentration required to cause 50% cell growth inhibition (IC_50_) was determined by interpolation from dose–response curves. All experiments were performed in triplicate.

### 3.6. Antioxidant Assay

Chemiluminescence (CL) [34] was applied to the antioxidant assay process. Chemiluminescence (CL) is a sensitive and accurate method for the measurement of the ability of samples to inhibit the generation of reactive oxygen species (ROS). The positive control was Vitamin E. In our study, we used phorbol 12-myristate 13-acetate (PMA) as stimulus for the production of different ROS by the phagocytic cells. PMA is activator of protein kinase C and an activator of nicotinamide adenine dinucleotide phosphate (NADPH) oxidase. Neutrophils stimulated with PMA give rise to robust chemiluminescence signals by a consequent increase in ROS production. The results were monitored by an Enspire Multimode Plate Reader, Perkin Elmer (EnSpire 2300, PerkinElmer, Singapore) as counts per second (CPS). Briefly, 40 µL diluted whole blood (1:25 dilution in sterile PBS, pH 7.4) or 40 µL poly morphonuclear neutrophils (PMN) (1 × 10^6^/mL) suspended in hanks balanced salt solution (HBSS++), were incubated with different concentrations of compounds. The cells were stimulated with 40 µL of PMA followed by lucigenin as an enhancer (0.5 mM), and then HBSS++ was added to adjust the final volume to 200 µL. The final concentrations of the samples in the mixture were 2.5 µM, 5 µM, 10 µM, 20 µM and 40 µM. Tests were performed in white 96-well microplates which were incubated at 22 °C for 30 min. Control wells contained HBSS++ alone, lucigenin with PMA and cells but no test compounds, and cells with positive control. The inhibition percentage (%) for each concentration was calculated using the following formula:

Inhibition percentage (%) = 100 − (CPS test / CPS control) × 100

## 4. Conclusions

Phytochemical investigation on DCM and EtOAc fractions from Heilaohu were carried out. Twenty-three lignans were isolated and identified by spectroscopic techniques such as 1D-, 2D-NMR and HR-ESI-MS, including three new dibenzocyclooctadiene lignans, heilaohulignans A–C (**1**–**3**), together with 20 known compounds. Among the known compounds, 12 compounds (**4**–**5**, **7**, **13**–**15** and **17**–**22**) were isolated from this species for the first time.

All isolated compounds were evaluated for their cytotoxicities and antioxidant bioassays. The new dibenzocyclooctadiene heilaohulignans A and B (**1**–**2**) did not exhibit potential activity on evaluation of cytotoxicity and antioxidant activity. Heilaohulignan C (**3**) demonstrated good cytotoxicity with IC_50_ value of 9.92 µM against HepG-2 human liver cancer cell line, as well as weak cytotoxicity against BGC-823 human gastric cancer cells and HCT-116 human colon cancer cells with IC_50_ values of 16.75 µM and 16.59 µM, respectively. Compounds **9** and **13** revealed weak cytotoxicity with IC_50_ values of 21.72 µM and 18.72 µM, respectively in HepG-2 human liver cancer cells. The chemiluminescence assay implied that compounds **11** and **20** showed weak activity with IC_50_ values of 25.56 µM and 21.20 µM, respectively. Consequently, the underlying cytotoxicity and antioxidant mechanisms of dibenzocyclooctadiene lignans, as well as their main active constituents, need to be further investigated and clarified, providing the material basis on the relationship between traditional uses and modern pharmacological activities.

## Figures and Tables

**Table 1:** ^1^H- (600 MHz) and ^13^C-NMR (150 MHz) data of compounds **1**, **2**, and **3** (CDCl_3_).

Number	1		2		3	
	*δ*_H_ (ppm) *J* (Hz)	*δ*_C_ (ppm)	*δ*_H_ (ppm) *J* (Hz)	*δ*_C_ (ppm)	*δ*_H_ (ppm) *J* (Hz)	*δ*_C_ (ppm)
1	−	141.7	−	143.0	−	147.0
2	−	138.8	−	140.2	−	133.6
3	−	151.5	−	152.2	−	150.5
4	6.71 s	113.0	6.58 s	113.5	6.41 s	106.9
5	−	134.8	−	131.3	−	133.5
6	2.65 m	38.9	2.50 m, 3.03 m	34.6	2.66 m	38.6
7	2.01 m	35.1	2.04 m	43.0	2.12 m	34.8
8	1.81 m	43.0	−	80.9	2.10 m	41.7
9	4.73 s	82.8	−	207.3	5.62 s	82.9
10	−	134.8	−	135.4	−	136.0
11	6.32 s	102.4	6.52 s	100.7	6.54 s	103.0
12	−	148.8	−	148.7	−	148.9
13	−	135.0	−	136.9	−	136.1
14	−	140.3	−	141.7	−	141.1
15	−	118.2	−	117.7	−	119.0
16	−	122.9	−	121.6	−	117.1
17	1.01 d (7.3)	15.3	1.33 s	23.3	1.09 d (7.0)	19.8
18	1.17 d (7.3)	20.0	0.89 d (7.1)	14.8	1.61 dd (7.1, 1.1)	14.2
19	5.93 dd (8.9, 1.4)	101.0	5.96 s, 6.02 s	101.6	5.98 s, 5.93 s	101.2
1′	−	176.7	−	173.1	−	167.5
2′	2.61 dt (13.9, 6.9)	34.0	2.43 m	41.5	−	127.6
3′	0.97 d (7.0)	18.7	1.40 m, 1.62 m	26.8	6.02 d (1.5)	137.2
4′	1.09 d (7.0)	18.7	0.86 t (7.4)	11.7	1.47 s	11.8
5′	−	−	1.02 d (7.0)	16.9	0.97 d (7.1)	15.0
2-OCH_3_	3.96 s	59.6	3.80 s	60.6	3.84 s	60.7
3-OCH_3_	3.78 s	61.1	3.86 s	56.1	3.84 s	59.8
14-OCH_3_	3.89 s	56.0	3.88 s	59.8	3.90 s	55.8

**Table 2:** Cytotoxicity data of compounds **3, 9** and **13**.

Compound	Cell Lines		
	Hep G-2	HCT-116	BGC-823
3	9.92	16.59	16.75
9	21.72	NO	NO
13	18.72	NO	NO
Taxol	≤0.10	≤0.10	≤0.10

Results are expressed as IC_50_ in µM; Taxol used as a positive control; ‘NO′ = no activity.

**Table 3:** Antioxidant activity data of compounds **11** and **20**.

Compounds	Neutrophils IC_50_ (µM)
11	25.56
20	21.20
Vitamin E	77.29

Results are expressed as IC_50_ in µM; Vitamin E used as a positive control.

## Supplementary Materials

The following are available online. Figure S1 is structures of compounds **1**–**23** isolated from Heilaohu. Figures S2–S19 are NMR data of new compounds (**1**–**3**); Figures S20–S59 are ^1^H- and ^13^C-NMR of known compounds (**4**–**23**); Figures S60–S62 HRESIMS spectrum of new compounds (**1**–**3**); Figures S63–S65 are CD spectrum of new compounds (**1**–**3**).
