# Supplementary material for: Lignans from Tujia Ethnomedicine Heilaohu: Chemical Characterization and Evaluation of Their Cytotoxicity and Antioxidant Activities
Source: Molecules. 2018 Aug 27;23(9):2147. doi: 10.3390/molecules23092147 (PMC6225210; doi:10.3390/molecules23092147)

Article

Lignans from Heilaohu: Chemical Characterization and Evaluation of their Cytotoxicity and Antioxidant Activities

Yongbei Liu[1,†], Yupei Yang[1,†], Shumaila Tasneem [1], Nusrat Hussain [1,4], Muhammad Daniyal [1], Hanwen Yuan [1], Qingling Xie [1], Bin Liu [2], Jing Sun [3], Yuqing Jian [1], Bin Li [1], Shenghuang Chen [1] and Wei Wang [1,3,4,*]

1 TCM and Ethnomedicine Innovation & Development International Laboratory, Innovative Drug Research Institute, School of Pharmacy, Hunan University of Chinese Medicine, Changsha, 410208, China; ybliu2018@163.com (Y.L.); yangyupei24@163.com (Y.Y.);; tasneemshum@gmail.com (S.T.); nusrat_hussain42@yahoo.com (N.H.) daniyaldani151@yahoo.com (M.D.); hanwyuan@hotmail.com (H. Y.); XieQL1992@163.com (Q. X.); cpujjyq2010@163.com (Y. J.); libin_hucm@hotmail.com (B.L.); cshtyh@163.com (S.C.)

2 College of Biology, Hunan Province Key Laboratory of Plant Functional Genomics and Developmental Regulation, Hunan University, Changsha 410082, China; binliu2001@hotmail.com

3 Shaanxi Key Laboratory of Basic and New herbal Medicament Research, Shaanxi Collaborative Innovation Center of Chinese Medicinal Resource Industrialization, Shaanxi University of Chinese Medicine; ph.175@163.com

4 H.E.J. Research Institute of Chemistry, International Center for Chemical and Biological Sciences, University of Karachi, Karachi-75270, Pakistan

* Correspondence: wangwei402@hotmail.com; Tel.: +86-136-5743-8606,

† These authors contributed equally to this work.

List of Figures

Figure S1. Structures of compounds **1-23** isolated from Heilaohu.
Figure S2. ^{1}H NMR (600 MHz, $CDCl_3$) spectrum of heilaohulignan A (**1**)
Figure S3. ^{13}C NMR (150 MHz, $CDCl_3$) spectrum of heilaohulignan A (**1**)
Figure S4. ^{1}H-^{1}H COSY NMR (600 MHz, $CDCl_3$) spectrum of heilaohulignan A (**1**)
Figure S5. HSQC NMR (600 MHz, $CDCl_3$) spectrum of heilaohulignanA (**1**)
Figure S6. HMBC NMR (600 MHz, $CDCl_3$) spectrum of heilaohulignan A (**1**)
Figure S7. NOESY NMR (600 MHz, $CDCl_3$) spectrum of heilaohulignan A (**1**)
Figure S8. ^{1}H NMR (600 MHz, $CDCl_3$) spectrum of heilaohulignan B (**2**)
Figure S9. ^{13}C NMR (150 MHz, $CDCl_3$) spectrum of heilaohulignanB (**2**)
Figure S10. ^{1}H-^{1}H COSY NMR (600 MHz, $CDCl_3$) spectrum of heilaohulignan B (**2**)
Figure S11. HSQC NMR (600 MHz, $CDCl_3$) spectrum of heilaohulignan B (**2**)
Figure S12. HMBC NMR (600 MHz, $CDCl_3$) spectrum of heilaohulignan B (**2**)
Figure S13. ROESY NMR (600 MHz, $CDCl_3$) spectrum of heilaohulignan B (**2**)
Figure S14. ^{1}H NMR (600 MHz, $CDCl_3$) spectrum of heilaohulignan C (**3**)
Figure S15. ^{13}C NMR (150 MHz, $CDCl_3$) spectrum of heilaohulignan C (**3**)
Figure S16. ^{1}H-^{1}H COSY NMR (600 MHz, $CDCl_3$) spectrum of heilaohulignan C (**3**)
Figure S17. HSQC NMR (600 MHz, $CDCl_3$) spectrum of heilaohulignan C (**3**)
Figure S18. HMBC NMR (600 MHz, $CDCl_3$) spectrum of heilaohulignan C (**3**)
Figure S19. ROESY NMR (600 MHz, $CDCl_3$) spectrum of heilaohulignan C (**3**)
Figure S20. ^{1}H NMR (600 MHz, $CDCl_3$) spectrum of schizandrin (**4**)
Figure S21. ^{13}C NMR (150 MHz, $CDCl_3$) spectrum of schizandrin (**4**)
Figure S22. ^{1}H NMR (600 MHz, $CDCl_3$) spectrum of binankadsurin A (**5**)
Figure S23. ^{13}C NMR (150 MHz, $CDCl_3$) spectrum of binankadsurin A (**5**)
Figure S24. ^{1}H NMR (600 MHz, $CDCl_3$) spectrum of acetylbinankadsurin A (**6**)
Figure S25. ^{13}C NMR (150 MHz, $CDCl_3$) spectrum of acetylbinankadsurin A (**6**)
Figure S26. ^{1}H NMR (600 MHz, MeOD) spectrum of isobutyroylbinankadsurin A (**7**)
Figure S27. ^{13}C NMR (150 MHz, MeOD) spectrum of isobutyroylbinankadsurin A (**7**)
Figure S28. ^{1}H NMR (600 MHz, $CDCl_3$) spectrum of isovaleroylbinankadsurin A (**8**)
Figure S29. ^{13}C NMR (150 MHz, $CDCl_3$) spectrum of isovaleroylbinankadsurin A (**8**)
Figure S30. ^{1}H NMR (600 MHz, $CDCl_3$) spectrum of kadsuralignan I (**9**)
Figure S31. ^{13}C NMR (150 MHz, $CDCl_3$) spectrum of kadsuralignan I (**9**)
Figure S32. ^{1}H NMR (600 MHz, $CDCl_3$) spectrum of kadsuralignan J (**10**)
Figure S33. ^{13}C NMR (150 MHz, $CDCl_3$) spectrum of kadsuralignan J (**10**)
Figure S34. ^{1}H NMR (600 MHz, $CDCl_3$) spectrum of kadsuralignan L (**11**)
Figure S35. ^{13}C NMR (150 MHz, $CDCl_3$) spectrum of kadsuralignan L (**11**)
Figure S36. ^{1}H NMR (600 MHz, $CDCl_3$) spectrum of kadsulignan N (**12**)
Figure S37. ^{13}C NMR (150 MHz, $CDCl_3$) spectrum of kadsulignan N (**12**)
Figure S38. ^{1}H NMR (600 MHz, $CDCl_3$) spectrum of longipedunin B (**13**)
Figure S39. ^{13}C NMR (150 MHz, $CDCl_3$) spectrum of longipedunin B (**13**)
Figure S40. ^{1}H NMR (600 MHz, $CDCl_3$) spectrum of schisantherin F (**14**)
Figure S41. ^{13}C NMR (150 MHz, $CDCl_3$) spectrum of schisantherin F (**14**)
Figure S42. ^{1}H NMR (600 MHz, MeOD) spectrum of schizanrin D (**15**)

Figure S43. ^{13}C NMR (150 MHz, MeOD) spectrum of schizanrin D (**15**)
Figure S44. ^{1}H NMR (600 MHz, MeOD) spectrum of acetylgomisin R (**16**)
Figure S45. ^{13}C NMR (150 MHz, MeOD) spectrum of acetylgomisin R (**16**)
Figure S46. 1H NMR (600 MHz, $CDCl_3$) spectrum of intermedin A (**17**)
Figure S47. ^{13}C NMR (150 MHz, $CDCl_3$) spectrum of intermedin A (**17**)
Figure S48. ^{1}H NMR (600 MHz, $CDCl_3$) spectrum of kadsurarin (**18**)
Figure S49. ^{13}C NMR (150 MHz, $CDCl_3$) spectrum of kadsurarin (**18**)
Figure S50. ^{1}H NMR (600 MHz, $CDCl_3$) spectrum of kadsutherin A (**19**)
Figure S51. ^{13}C NMR (150 MHz, $CDCl_3$) spectrum of kadsutherin A (**19**)
Figure S52. ^{1}H NMR (600 MHz, $CDCl_3$) spectrum of kadsuphilol A (**20**)
Figure S53. ^{13}C NMR (150 MHz, $CDCl_3$) spectrum of kadsuphilol A (**20**)
Figure S54. ^{1}H NMR (600 MHz, $CDCl_3$) spectrum of meso-dihydroguaiaretic acid dimethyl (**21**)
Figure S55. ^{13}C NMR (150 MHz, $CDCl_3$) spectrum of meso-dihydroguaiaretic acid dimethyl (**21**)
Figure S56. ^{1}H NMR (600 MHz, $CDCl_3$) spectrum of schiarianrin E (**22**)
Figure S57. ^{13}C NMR (150 MHz, $CDCl_3$) spectrum of schiarianrin E (**22**)
Figure S58. ^{1}H NMR (600 MHz, $CDCl_3$) spectrum of schiarisanrin A (**23**)
Figure S59. ^{13}C NMR (150 MHz, $CDCl_3$) spectrum of schiarisanrin A (**23**)
Figure S60. HRESIMS spectrum of heilaohulignan A (**1**)
Figure S61. HRESIMS spectrum of heilaohulignan B (**2**)
Figure S62. HRESIMS spectrum of heilaohulignan C (**3**)
Figure S63. CD spectrum of heilaohulignan A (**1**)
Figure S64. CD spectrum of heilaohulignan B (**2**)
Figure S65. CD spectrum of heilaohulignan C (**3**)

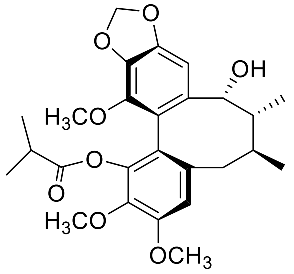

1

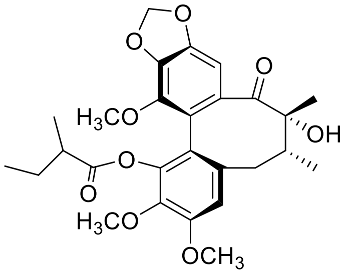

2

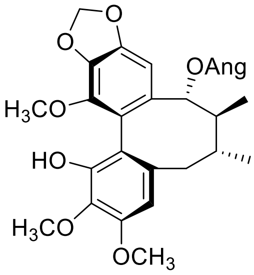

3

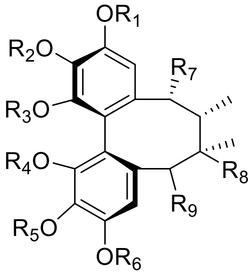

4 R_1=R_2=R_3=R_4=R_5=R_6=CH_3, R_7=R_9=H, R_8=OH
5 R_5+R_6=CH_2, R_1=R_2=R_4=CH_3, R_3=R_7=R_8=H, R_9=α OH
6 R_1+R_2=CH_2, R_3=R_5=R_6=CH_3, R_4=R_8=R_9=H, R_7=OAc
7 R_1+R_2=CH_2, R_3=R_5=R_6=CH_3, R_4=R_8=R_9=H, R_7=OIsobut
8 R_1+R_2=CH_2, R_3=R_5=R_6=CH_3, R_4=R_8=R_9=H, R_7=OIsoval
9 R_1+R_2=CH_2, R_3=R_5=R_6=CH_3, R_8=R_9=H, R_7=OH, R_4=OAng
10 R_1+R_2=CH_2, R_3=R_5=R_6=CH_3, R_8=R_9=H, R_7=OH, R_4=OIsoval
11 R_2=R_3=R_5=R_6=CH_3, R_1=R_8=R_9=H, R_7=OH, R_4=OIsoval
13 R_1+R_2=CH_2, R_3=R_5=R_6=CH_3, R_4=OH, R_8=R_9=H, R_7=OProp
14 R_2=R_3=R_5=R_6=CH_3, R_1=R_8=R_9=H, R_7=OH, R_4=OAng
15 R_1+R_2=CH_2, R_4=R_5=R_6=CH_3, R_3=R_8=R_9=H, R_7=OTig
16 R_1+R_2=R_5+R_6=CH_2, R_3=R_4=CH_3, R_9=R_8=H, R_7=OAc
18 R_1+R_2=CH_2, R_3=R_5=R_6=CH_3, R_4=R_8=H, R_7=OAc, R_9=α OAng
19 R_5+R_6=CH_2, R_1=R_2=CH_3, R_3=R_4=R_7=R_8=H, R_9=OAng
20 R_5+R_6=CH_2, R_1=R_2=R_4=CH_3, R_3=R_7=R_8=H, R_9=β OH

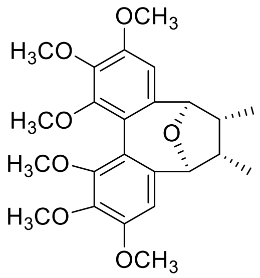

12

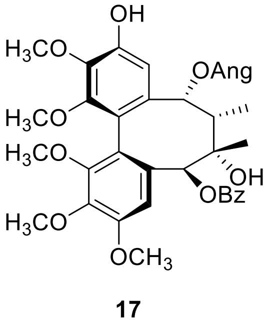

17

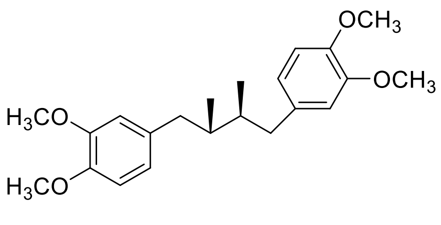

21

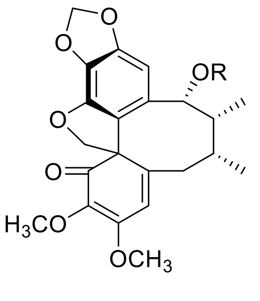

22 R=Ang
23 R=Isoval

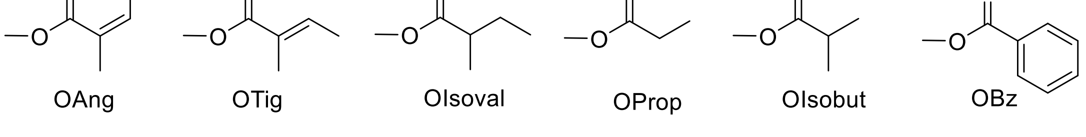

OAng OTig OIsoval OProp OIsobut OBz

Figure S1. Structures of compounds **1-23** isolated from Heilaohu.

Figure S2. ^{1}H NMR (600 MHz, $CDCl_3$) spectrum of heilaohulignan A (**1**)

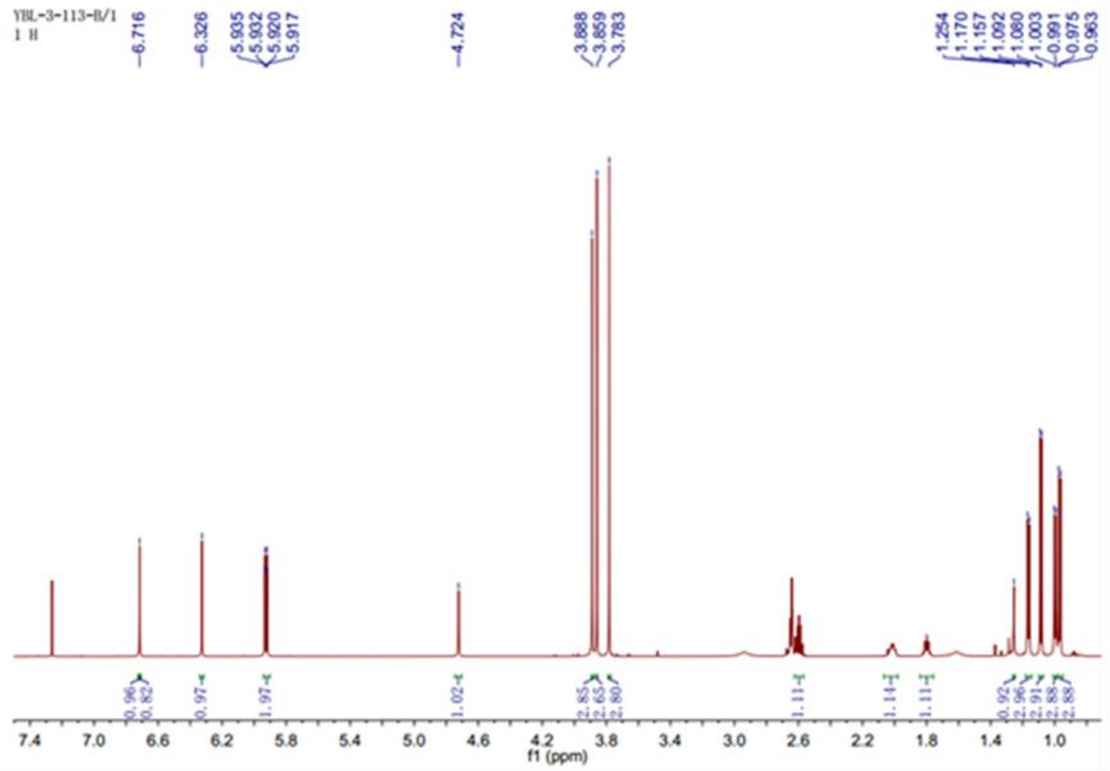

Figure S3. ^{13}C NMR (150 MHz, $CDCl_3$) spectrum of heilaohulignan A (**1**)

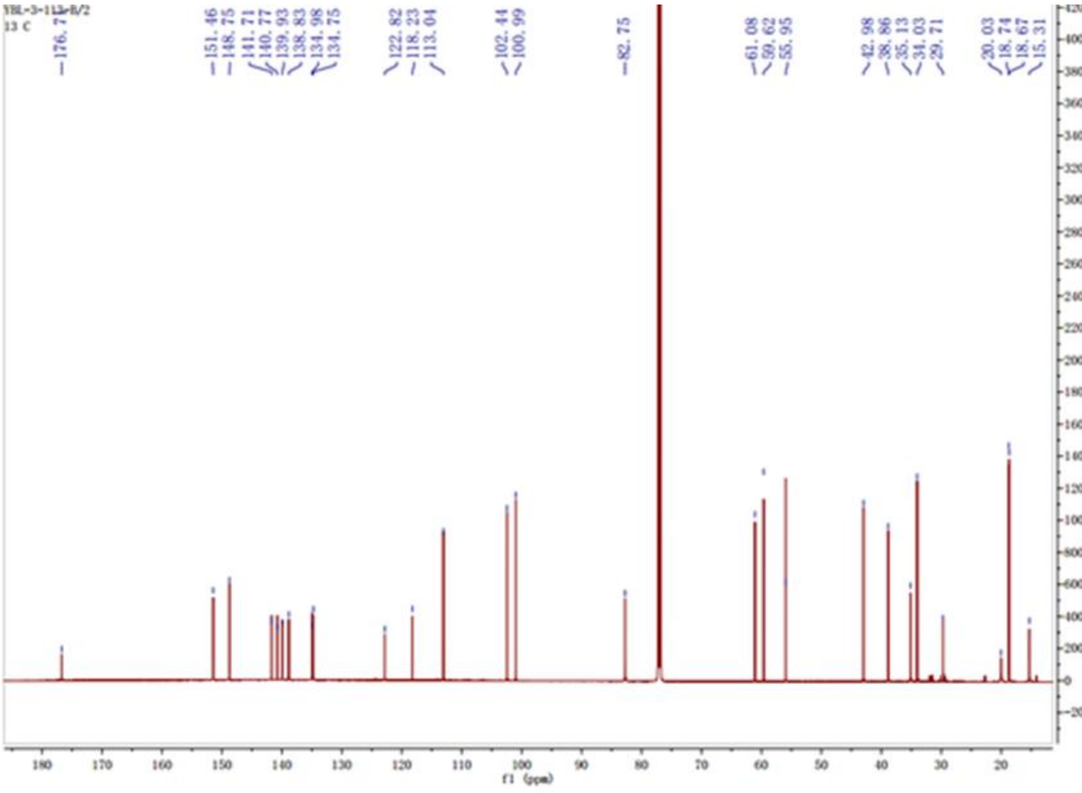

Figure S4. ^{1}H-^{1}H COSY NMR (600 MHz, $CDCl_3$) spectrum of heilaohulignan A (**1**)

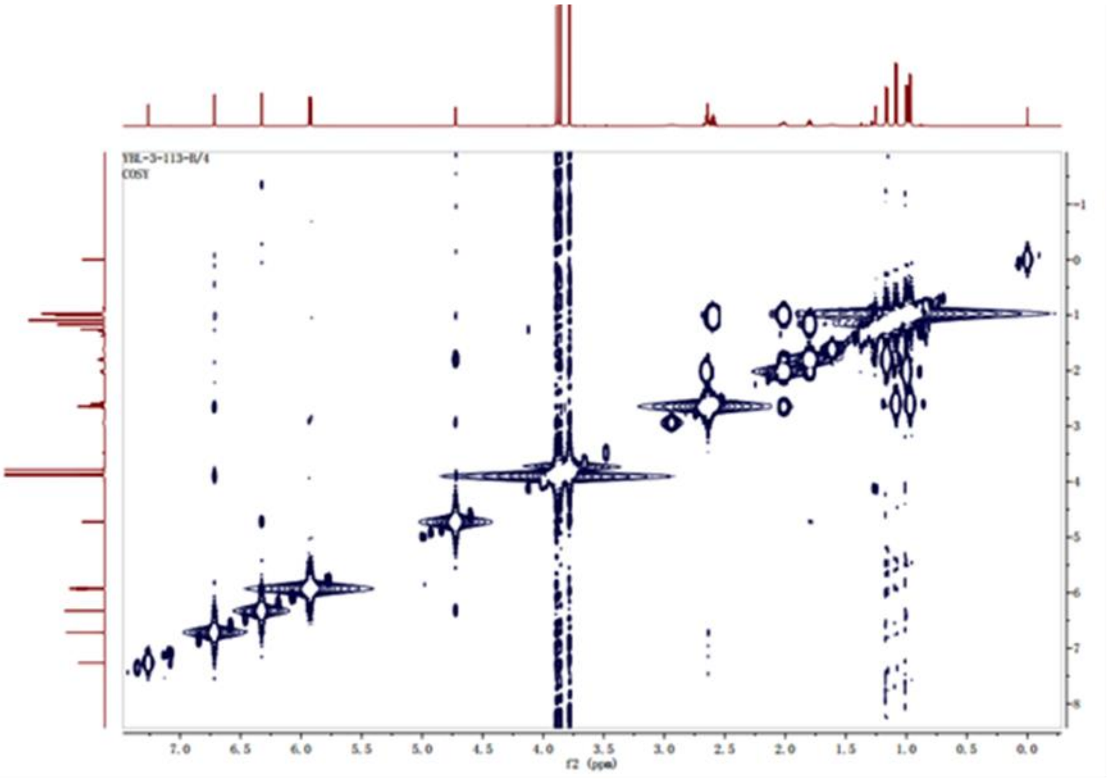

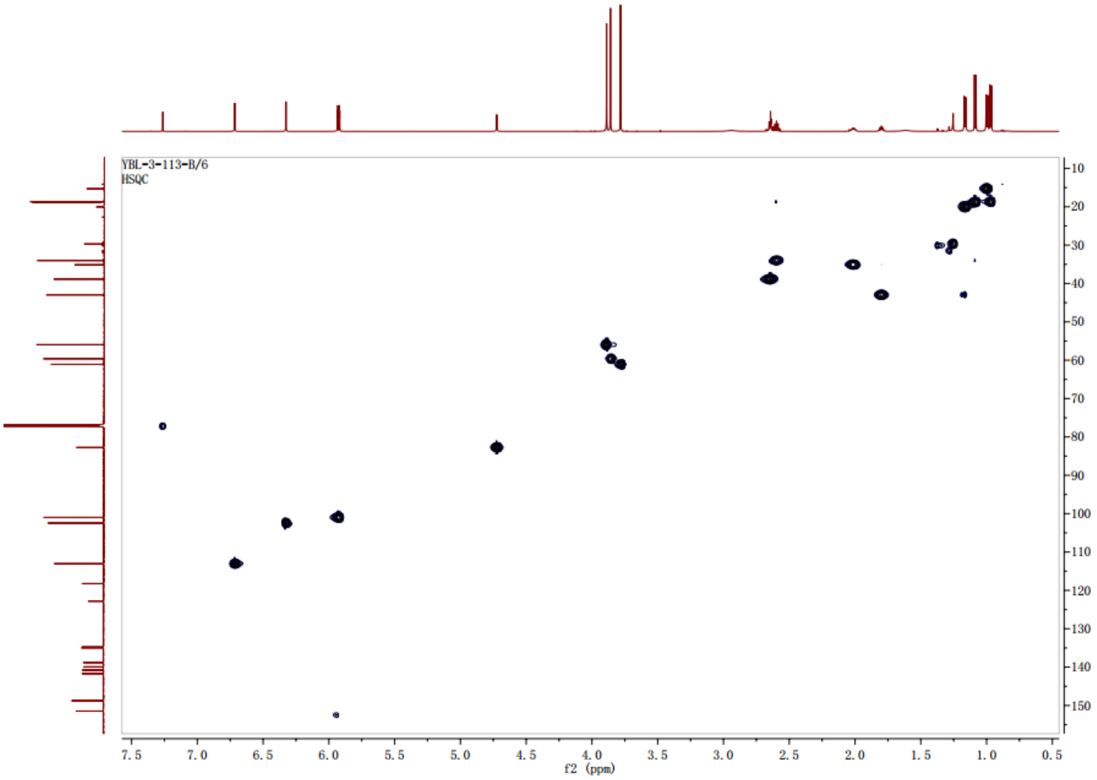

Figure S5. HSQC NMR (600 MHz, $CDCl_3$) spectrum of heilaohulignan A (**1**)

Figure S6. HMBC NMR (600 MHz, CDCl3) spectrum of heilaohulignan A (**1**)

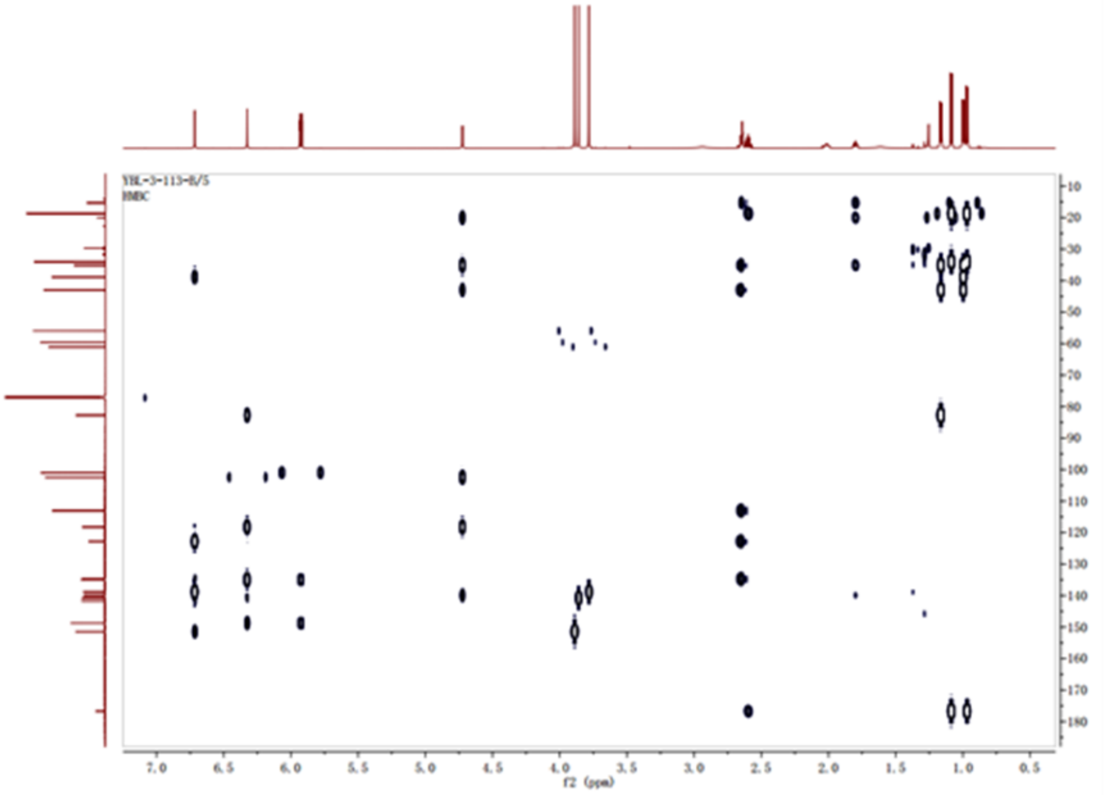

Figure S7. NOESY NMR (600 MHz, CDCl$_3$) spectrum of heilaohulignan A (1)

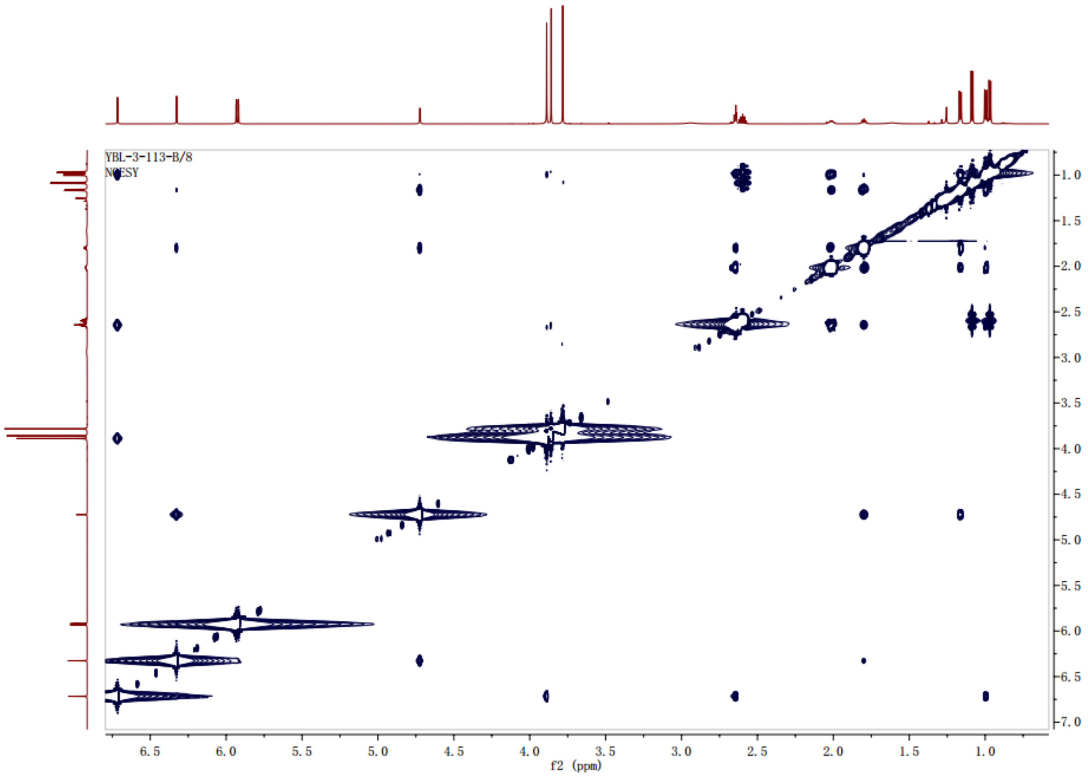

Figure S8. ^{1}H NMR (600 MHz, CDCl$_3$) spectrum of heilaohulignan B (**2**)

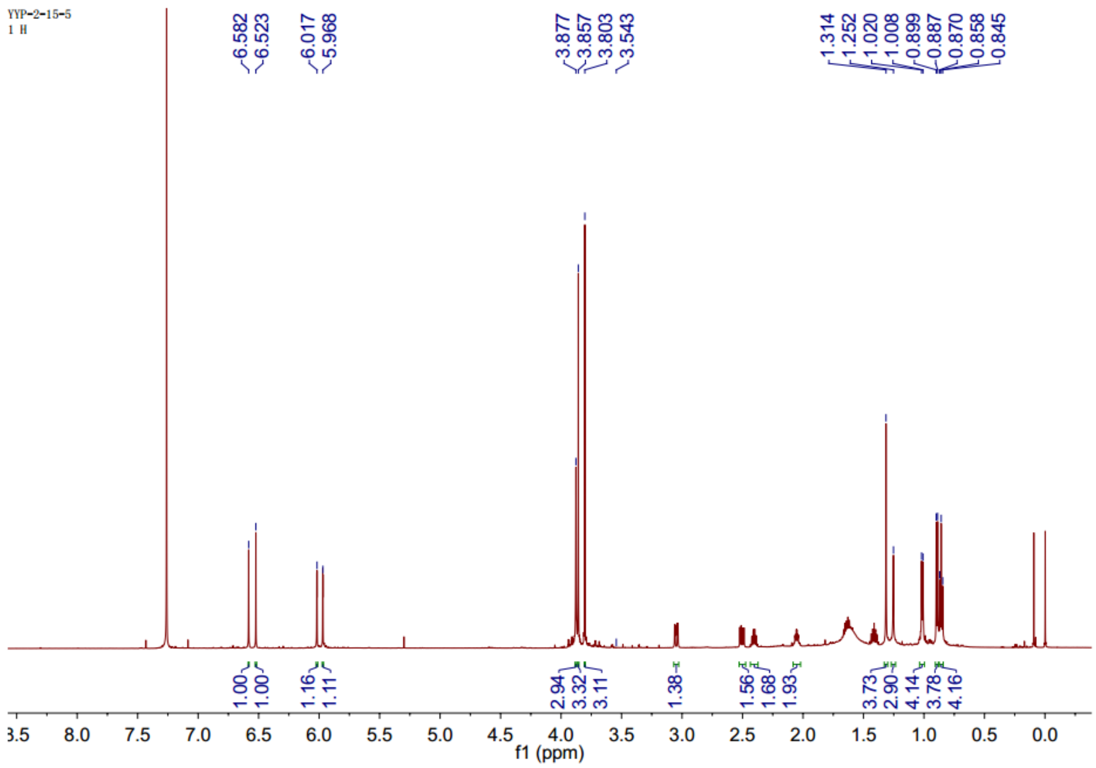

Figure S9. ^{13}C NMR (150 MHz, $CDCl_3$) spectrum of heilaohulignan B (**2**)

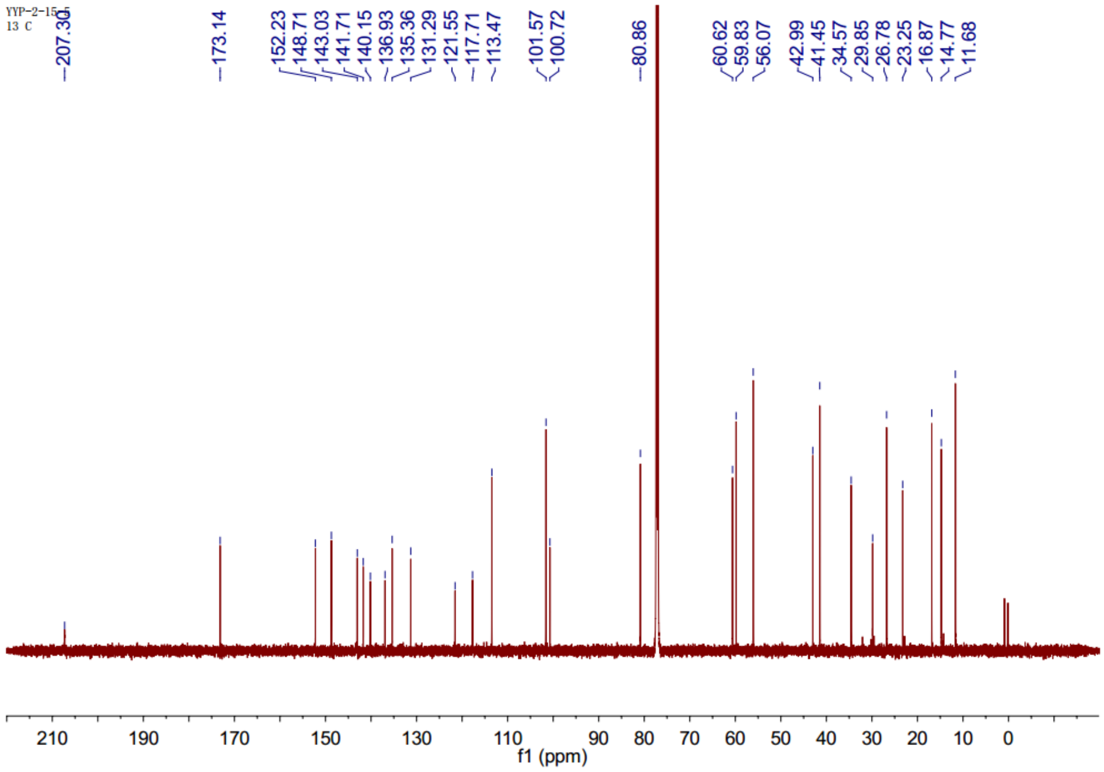

Figure S10. ^1H-^1H COSY NMR (600 MHz, $CDCl_3$) spectrum of heilaohulignan B (**2**)

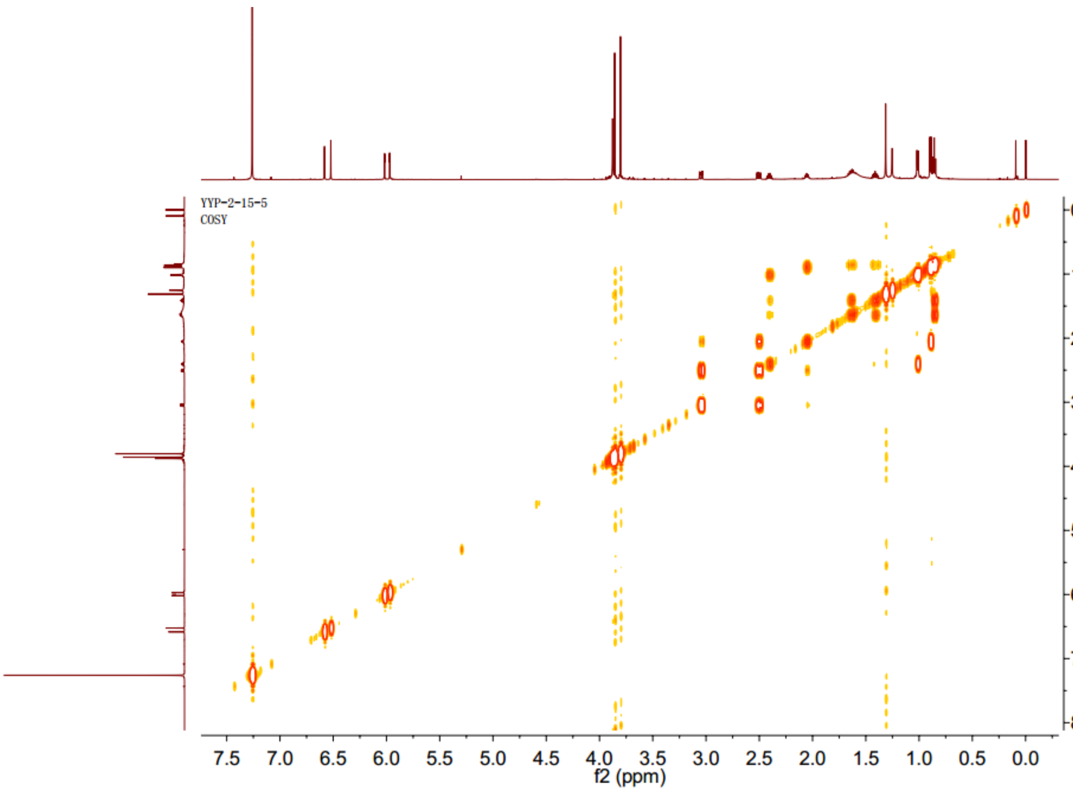

Figure S11. HSQC NMR (600 MHz, $CDCl_3$) spectrum of heilaohulignan B (**2**)

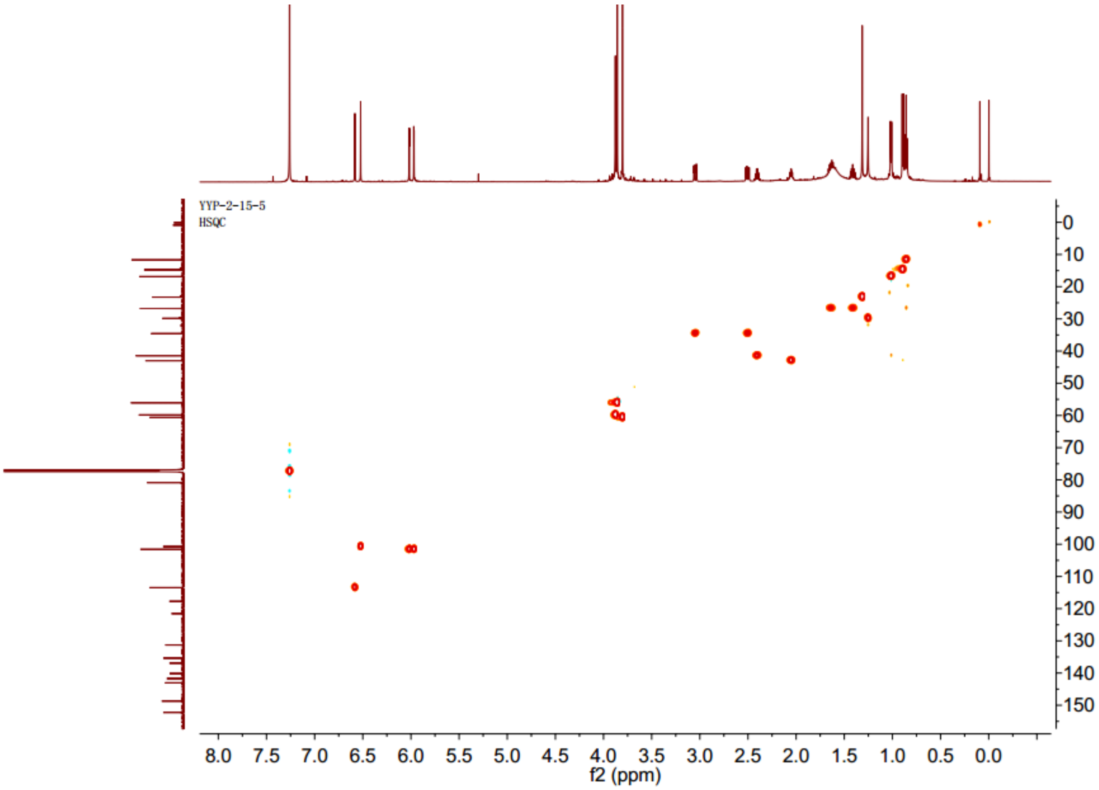

Figure S12. HMBC NMR (600 MHz, $CDCl_3$) spectrum of heilaohulignan B (**2**)

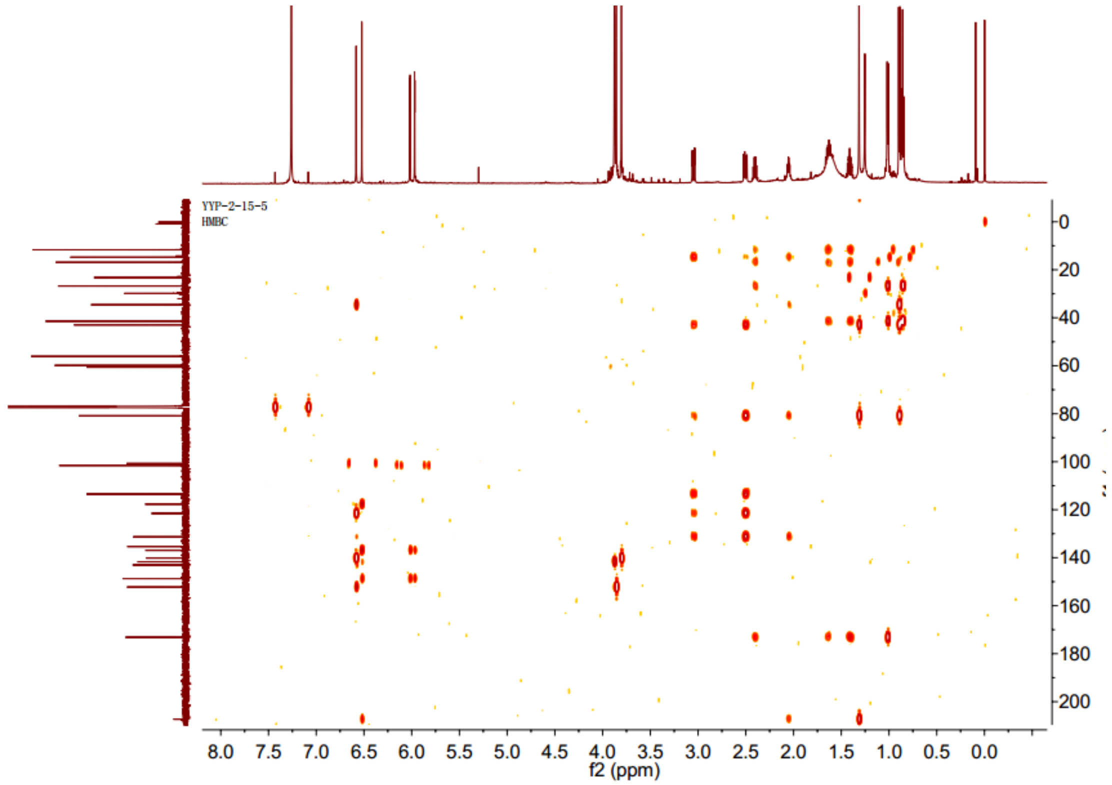

Figure S13. ROESY NMR (600 MHz, $CDCl_3$) spectrum of heilaohulignan B (**2**)

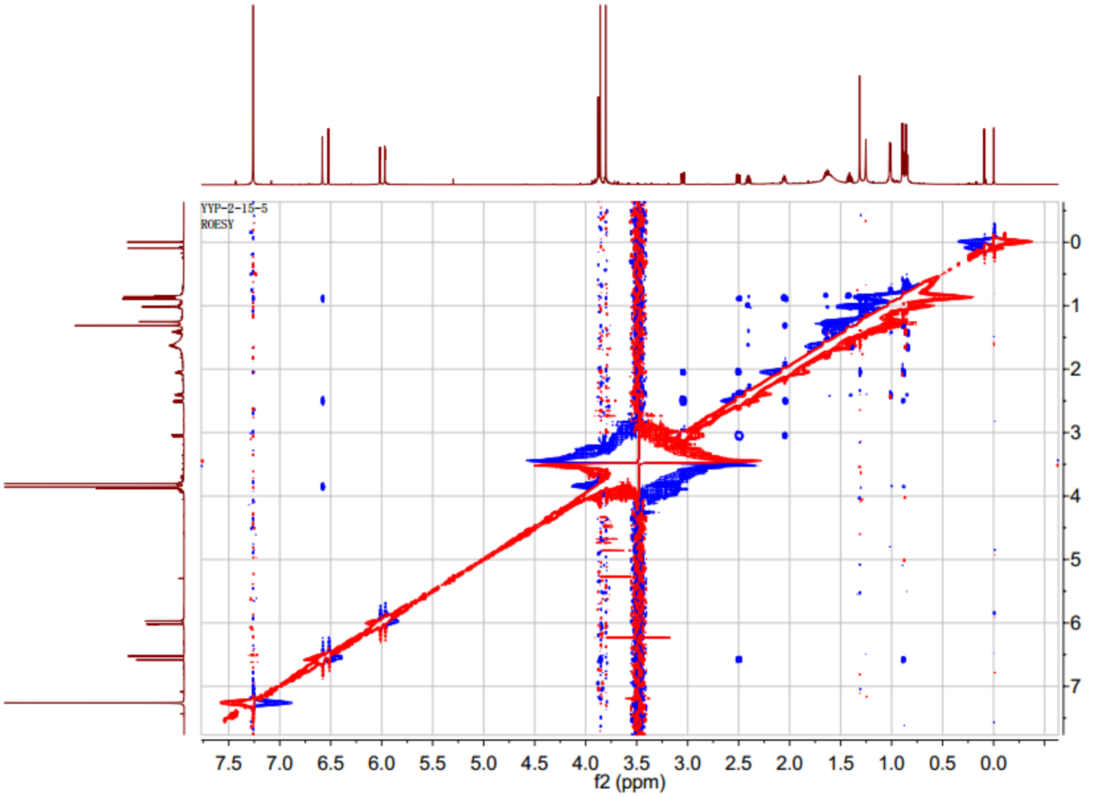

Figure S14. ^{1}H NMR (600 MHz, $CDCl_3$) spectrum of heilaohulignan C (**3**)

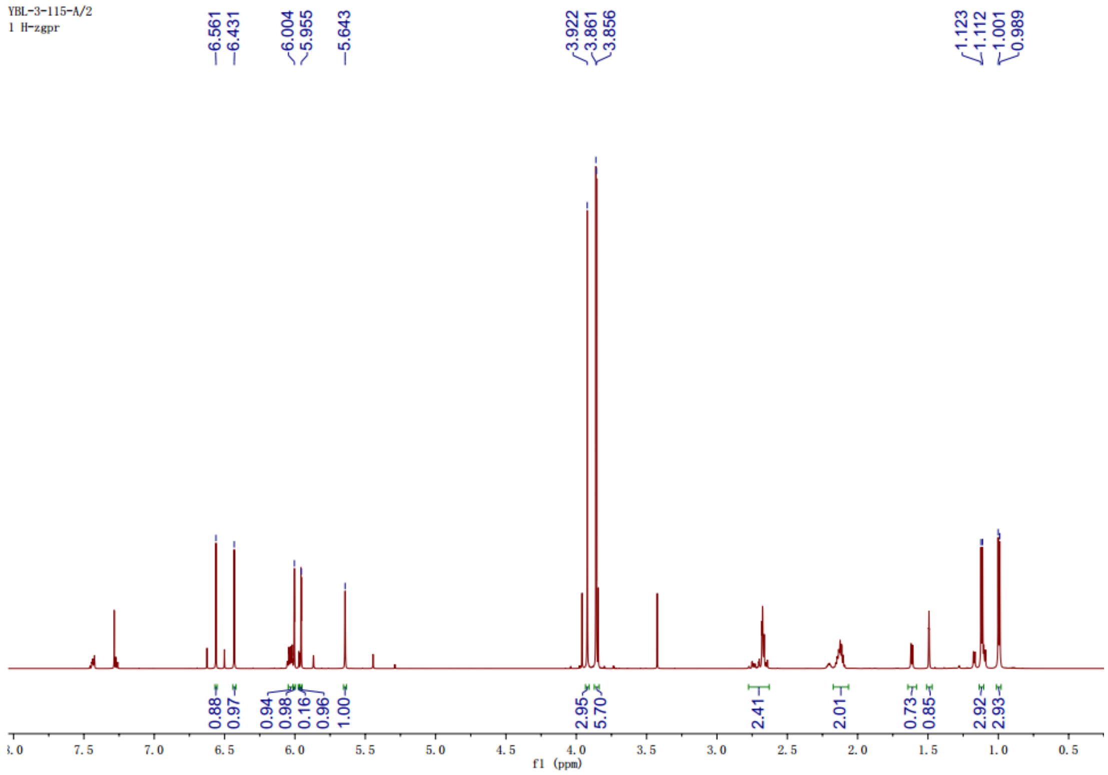

Figure S15. ^{13}C NMR (150 MHz, $CDCl_3$) spectrum of heilaohulignan C (**3**)

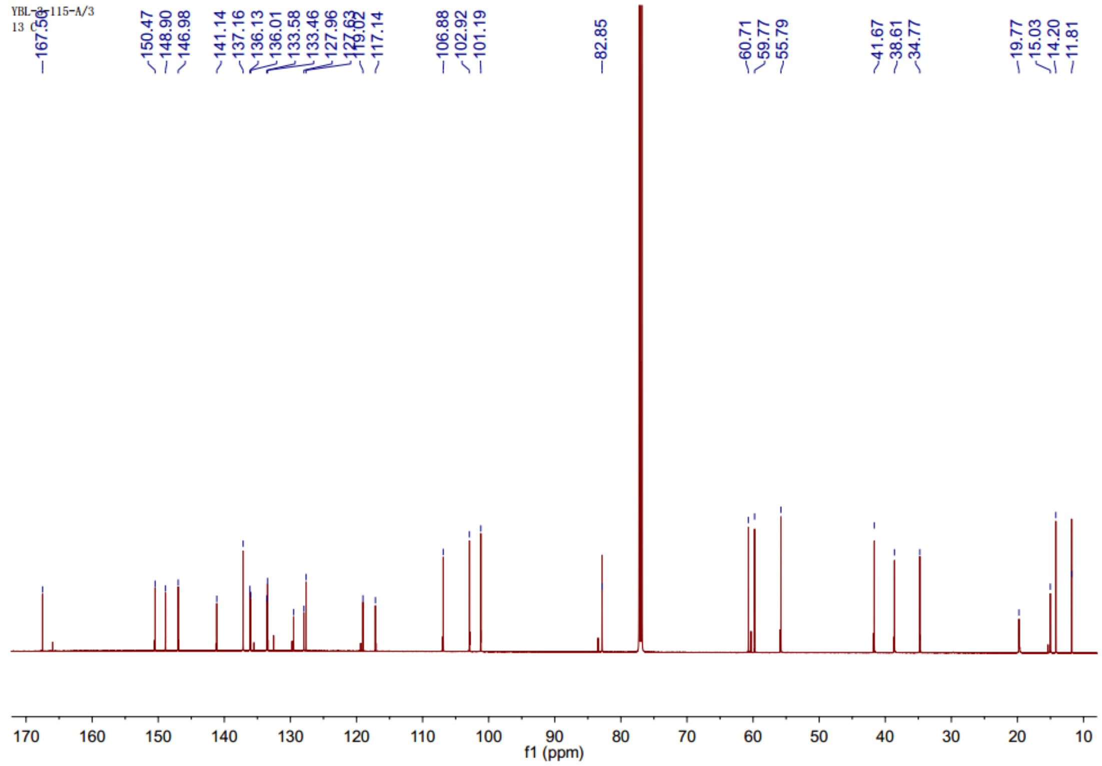

Figure S16. ^1H-^1H COSY NMR (600 MHz, $CDCl_3$) spectrum of heilaohulignan C (**3**)

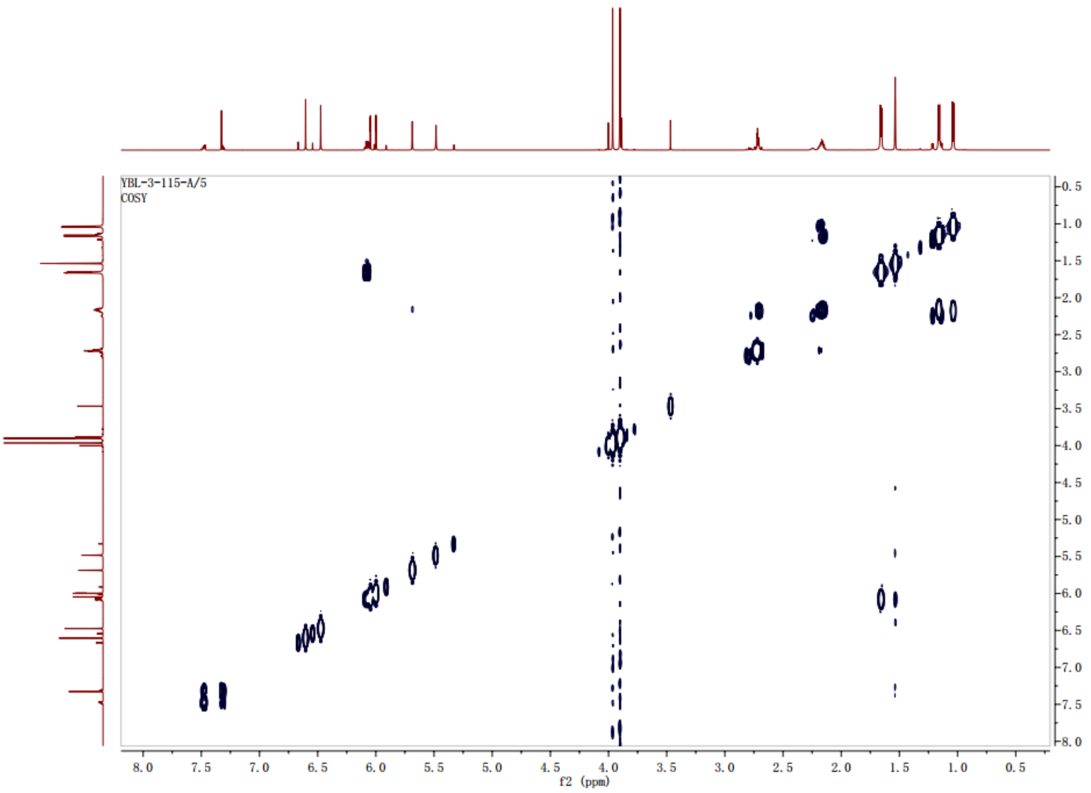

Figure S17. HSQC NMR (600 MHz, $CDCl_3$) spectrum of heilaohulignan C (**3**)

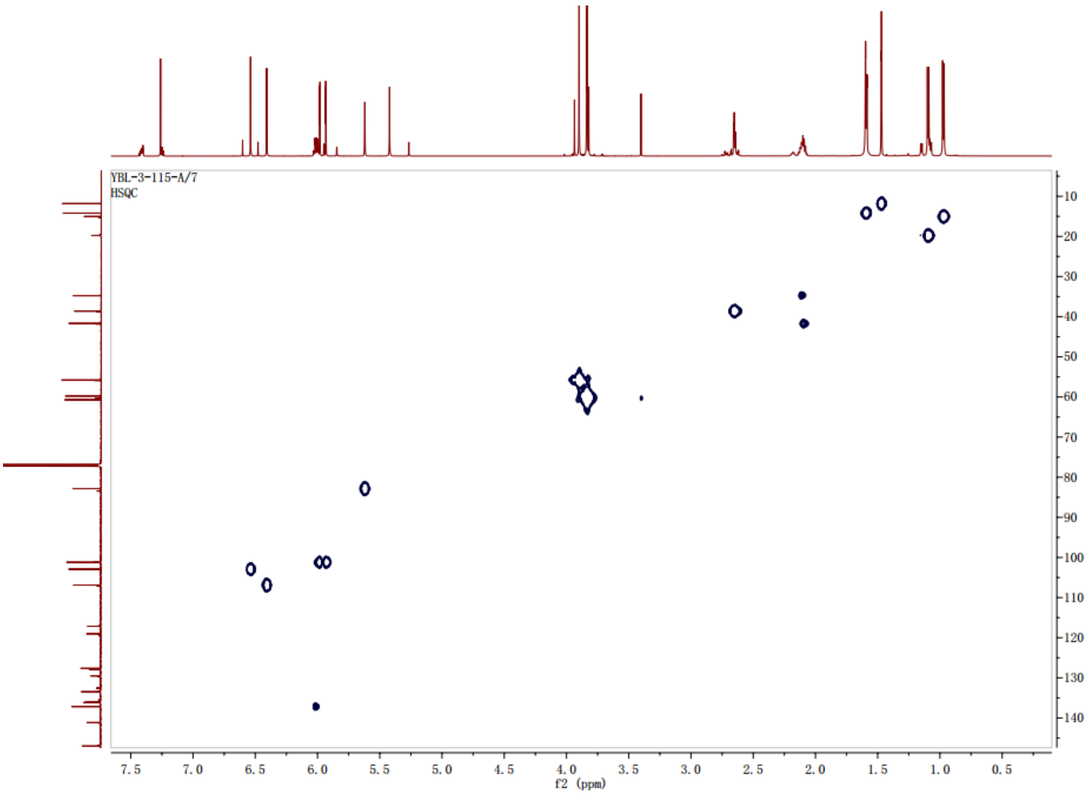

Figure S18. HMBC NMR (600 MHz, $CDCl_3$) spectrum of heilaohulignan C (**3**)

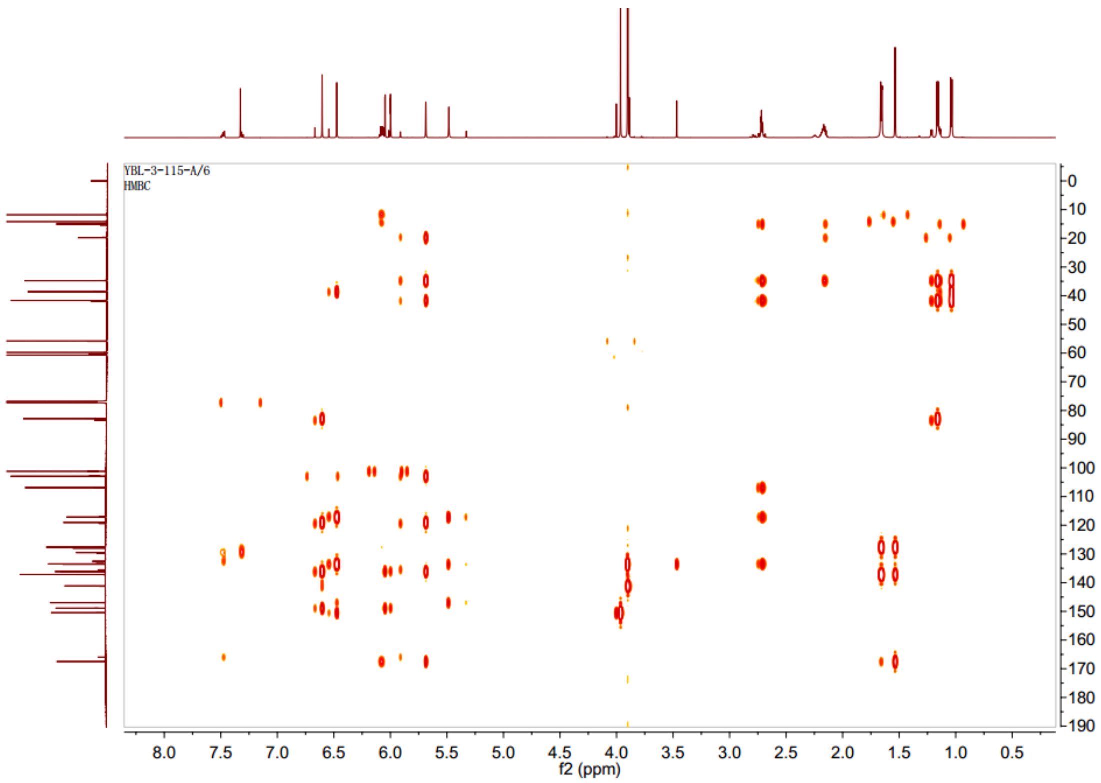

Figure S19. ROESY NMR (600 MHz, $CDCl_3$) spectrum of heilaohulignan C (**3**)

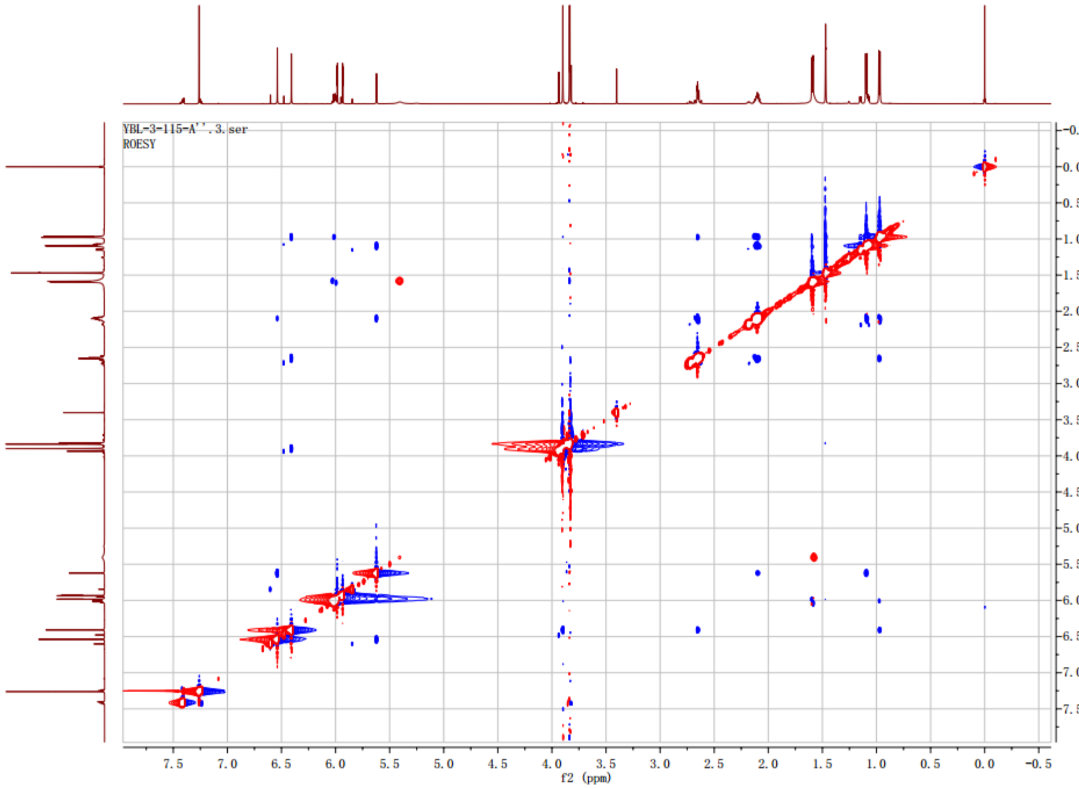

Figure S20. ^{1}H NMR (600 MHz, $CDCl_3$) spectrum of schizandrin (**4**)

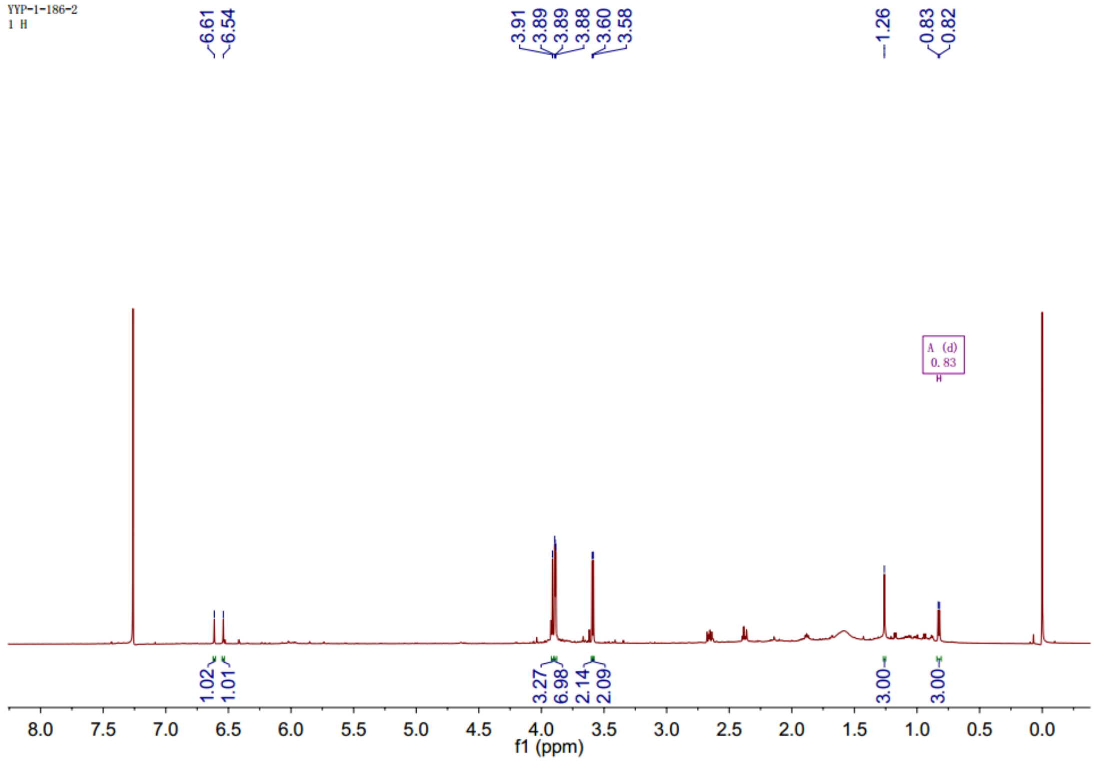

Figure S21. ^{13}C NMR (150 MHz, $CDCl_3$) spectrum of schizandrin (**4**)

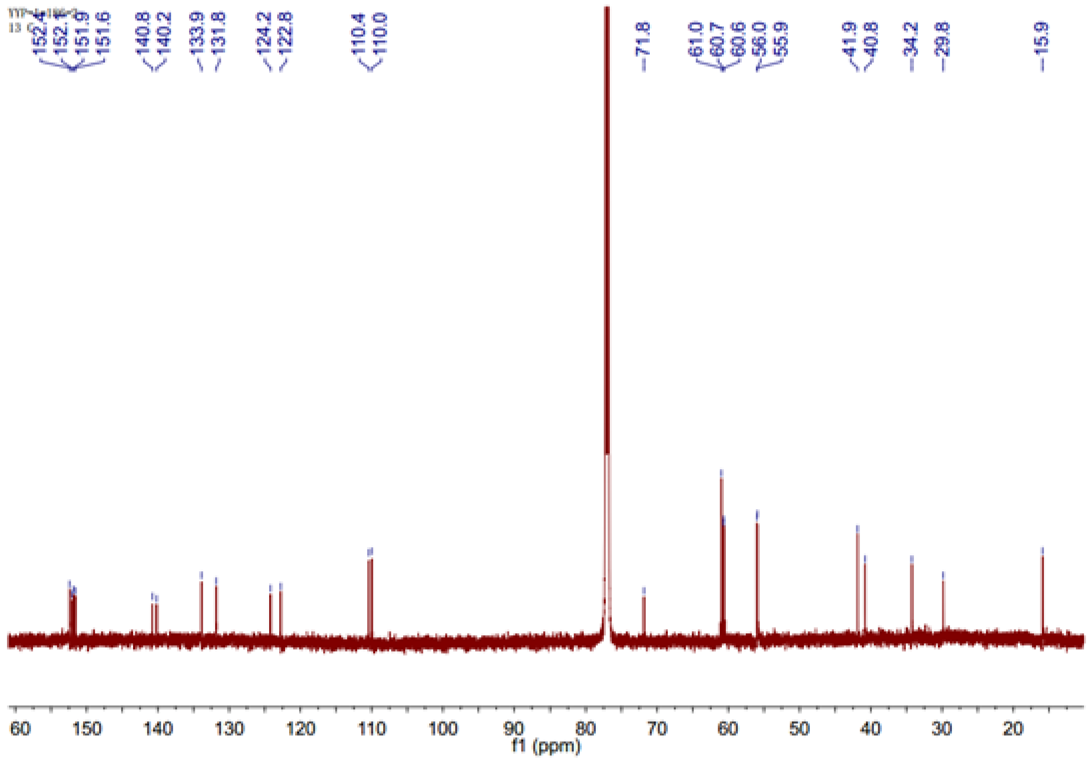

Figure S22. ^{1}H NMR (600 MHz, $CDCl_3$) spectrum of binankadsurin A (**5**)

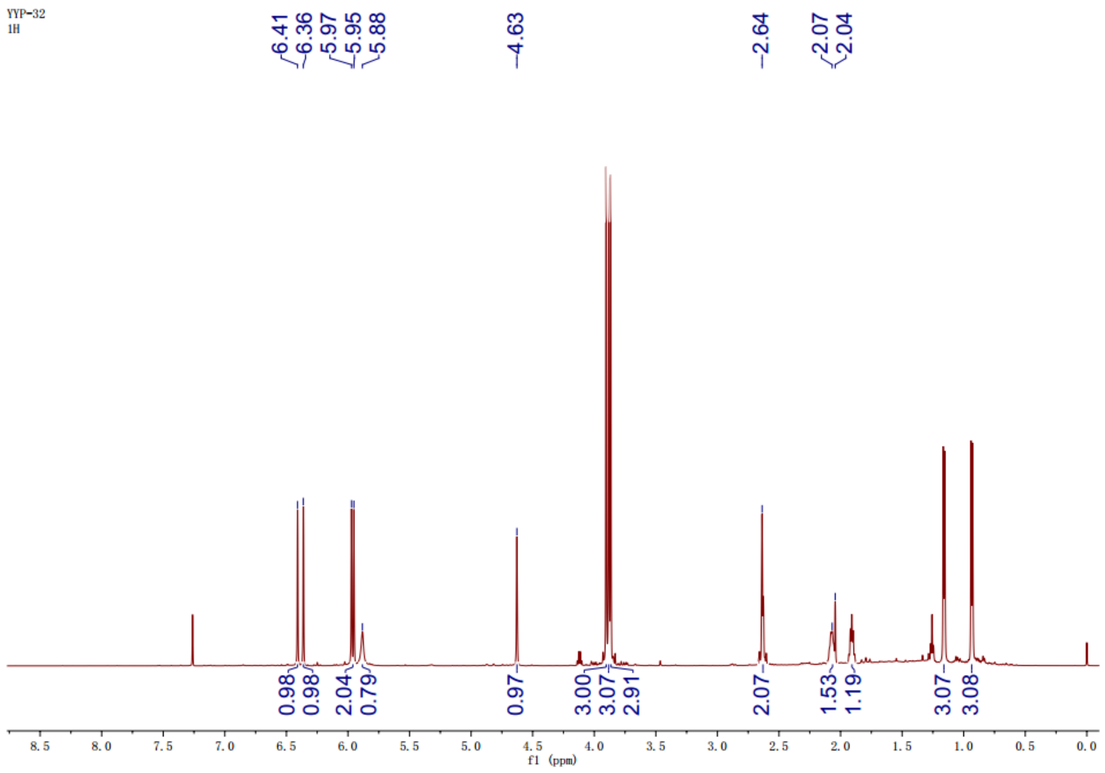

Figure S23. ^{13}C NMR (150 MHz, $CDCl_3$) spectrum of binankadsurin A (**5**)

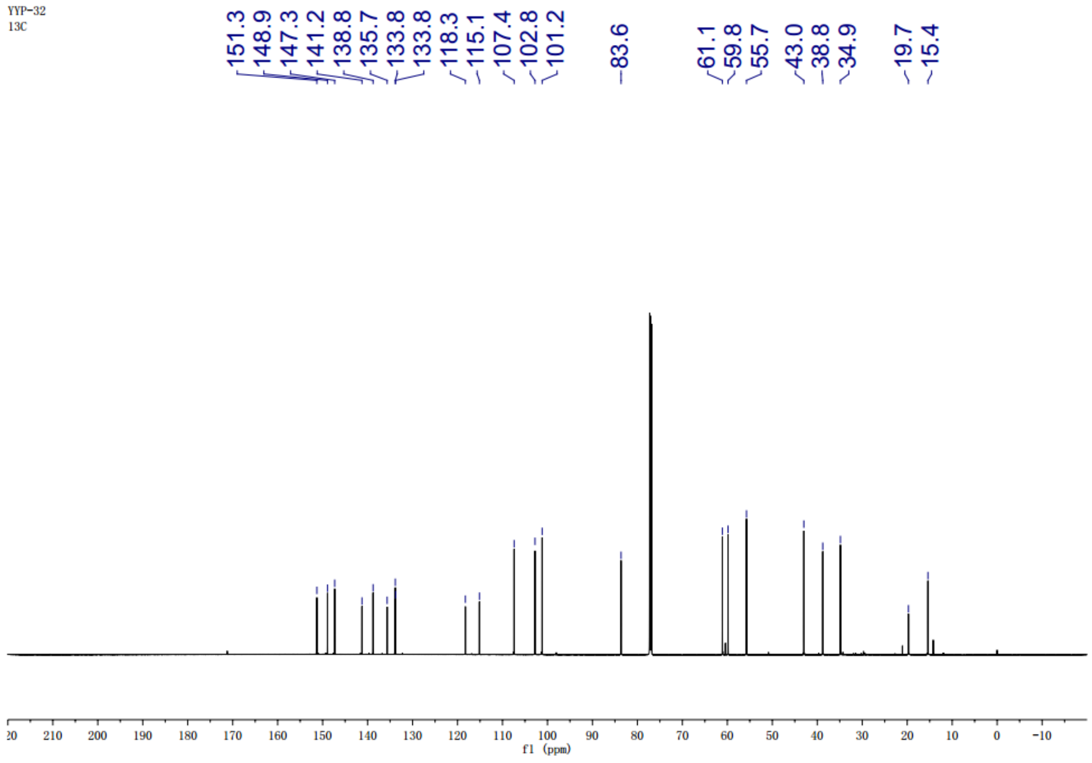

Figure S24. ^{1}H NMR (600 MHz, $CDCl_3$) spectrum of acetylbinankadsurin A (**6**)

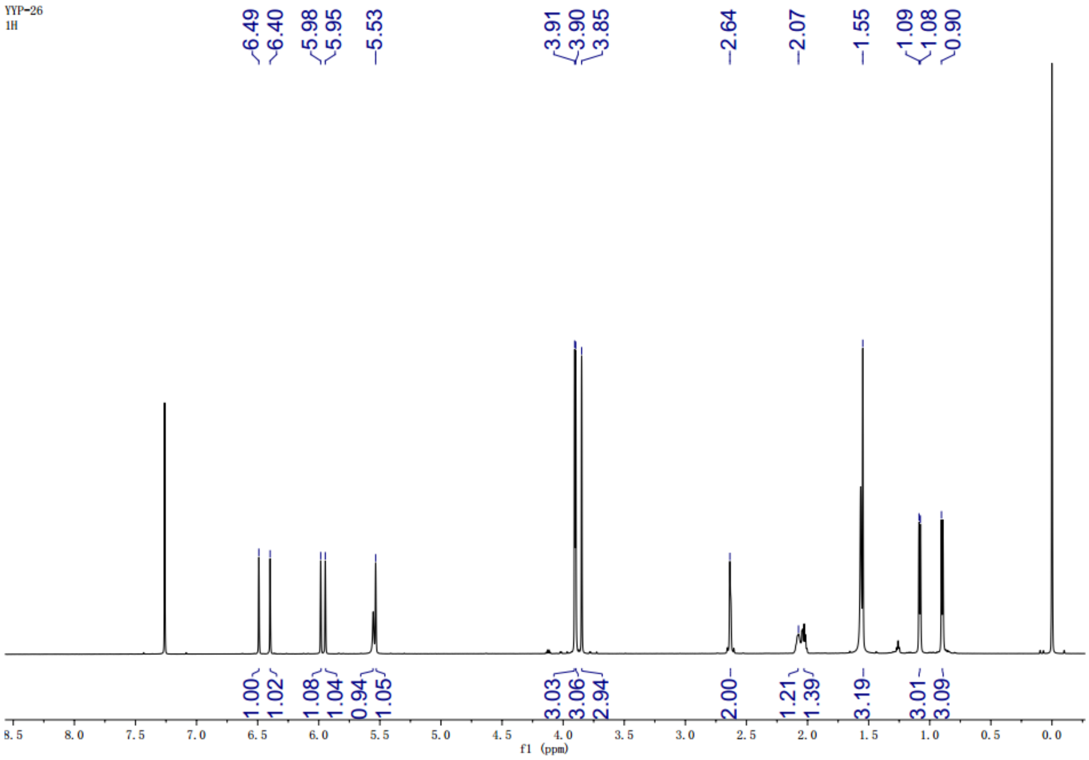

Figure S25. ^{13}C NMR (150 MHz, $CDCl_3$) spectrum of acetylbinankadsurin A (**6**)

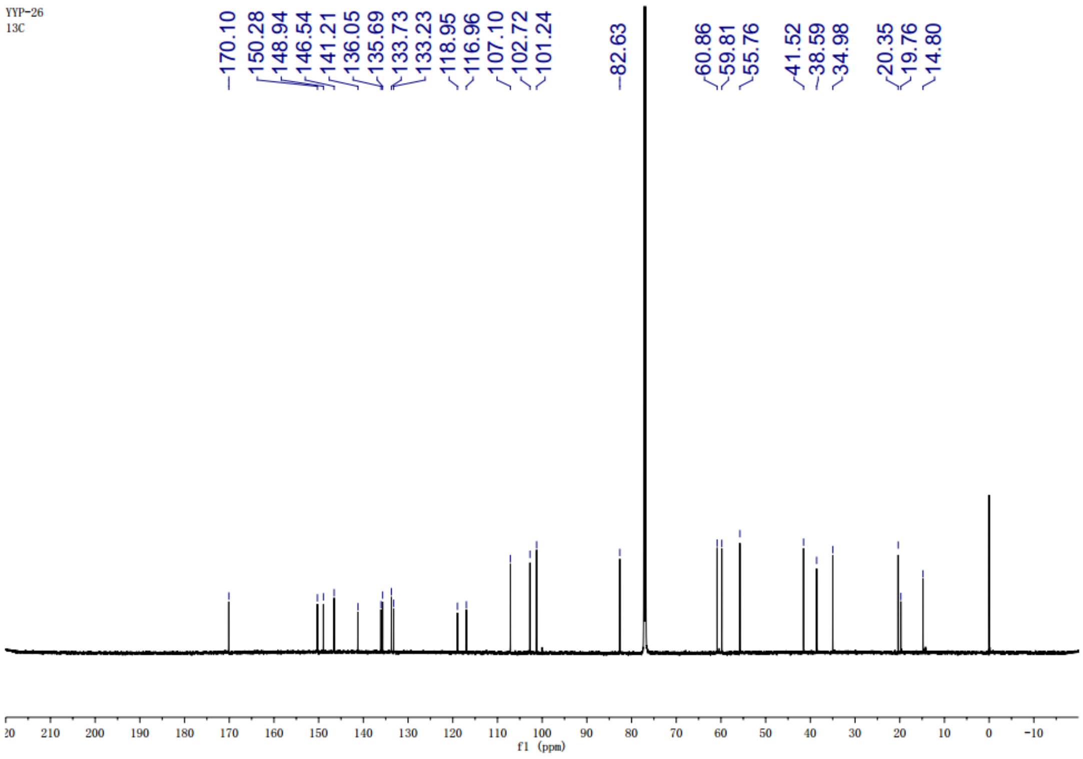

Figure S26. ^{1}H NMR (600 MHz, MeOD) spectrum of isobutyroylbinankadsurin A (**7**)

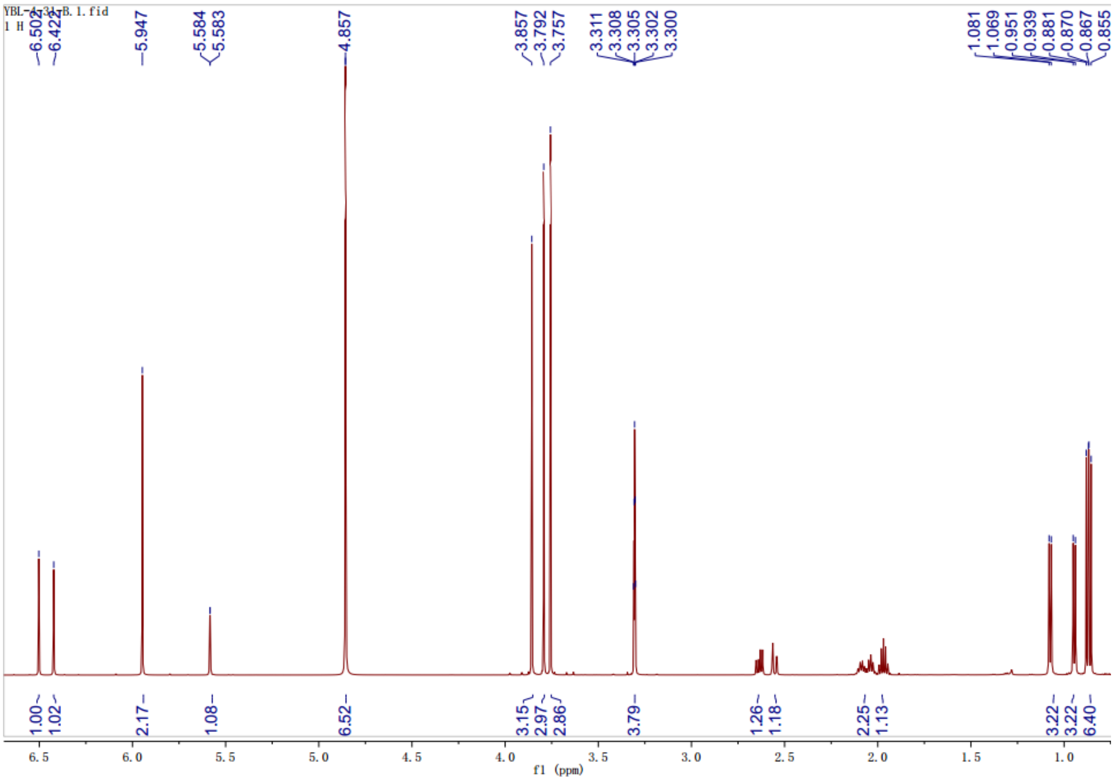

Figure S27. ^{13}C NMR (150 MHz, MeOD) spectrum of isobutyroylbinankadsurin A (**7**)

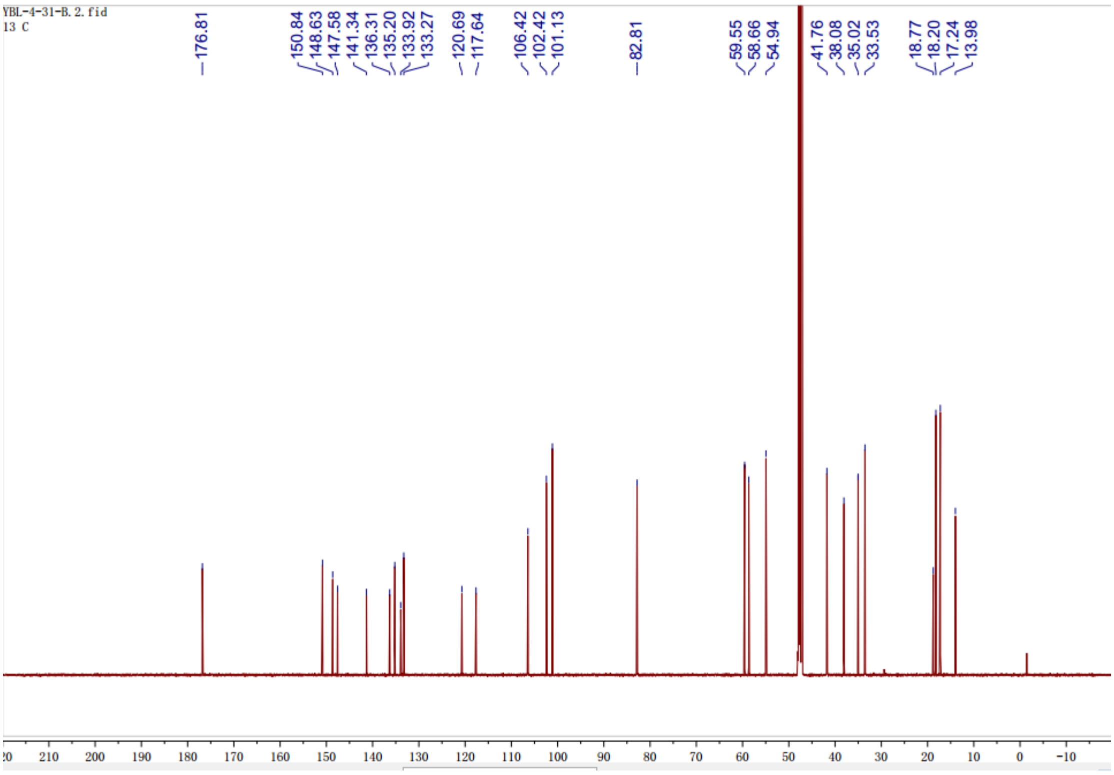

Figure S28. ^{1}H NMR (600 MHz, $CDCl_3$) spectrum of isovaleroylbinankadsurin A (**8**)

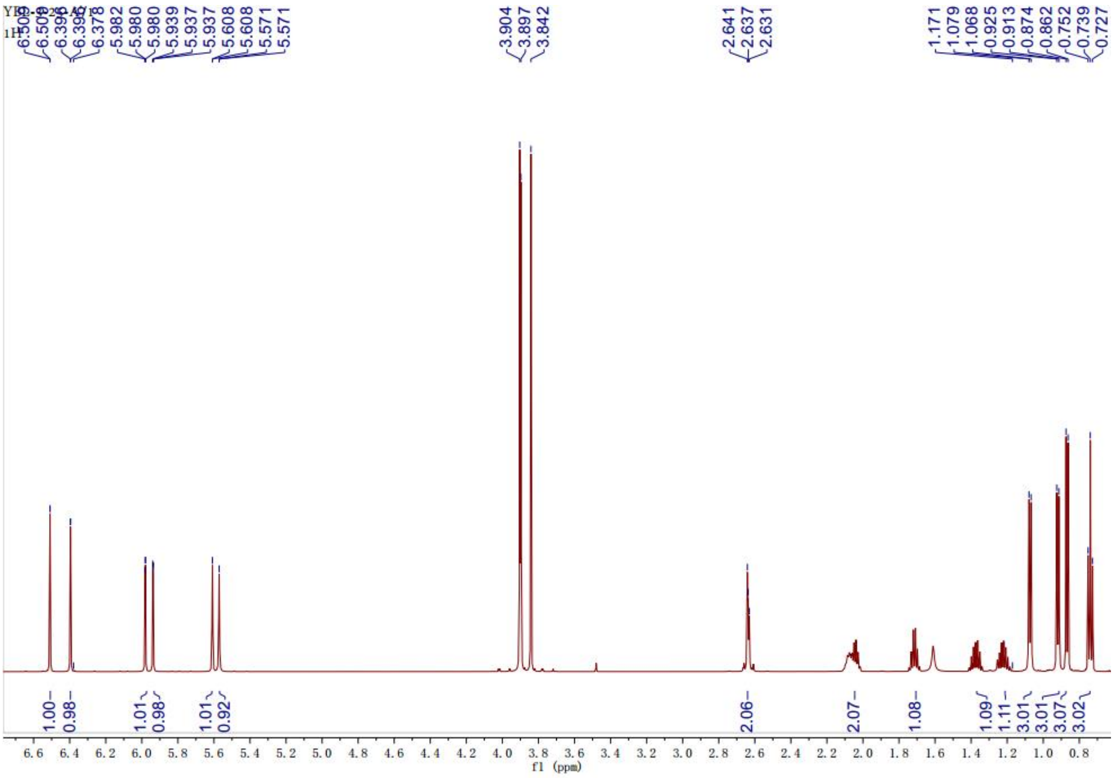

Figure S29. ^{13}C NMR (150 MHz, $CDCl_3$) spectrum of isovaleroylbinankadsurin A (**8**)

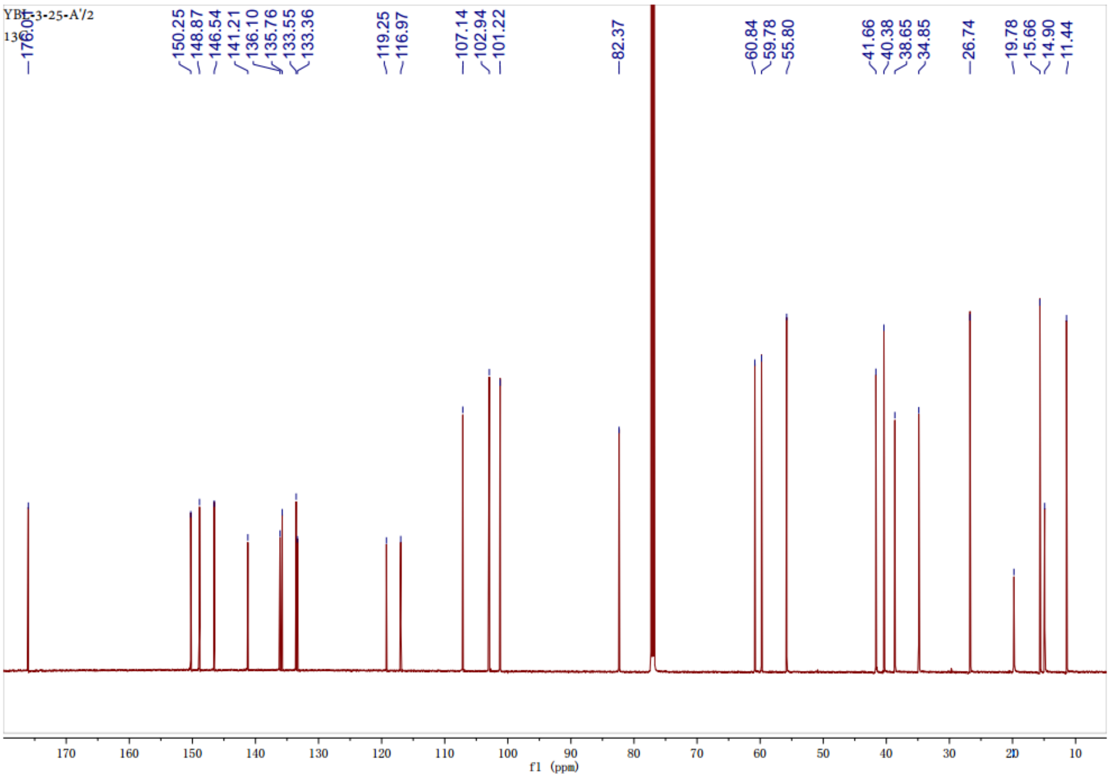

Figure S30. ^{1}H NMR (600 MHz, $CDCl_3$) spectrum of kadsuralignan I (**9**)

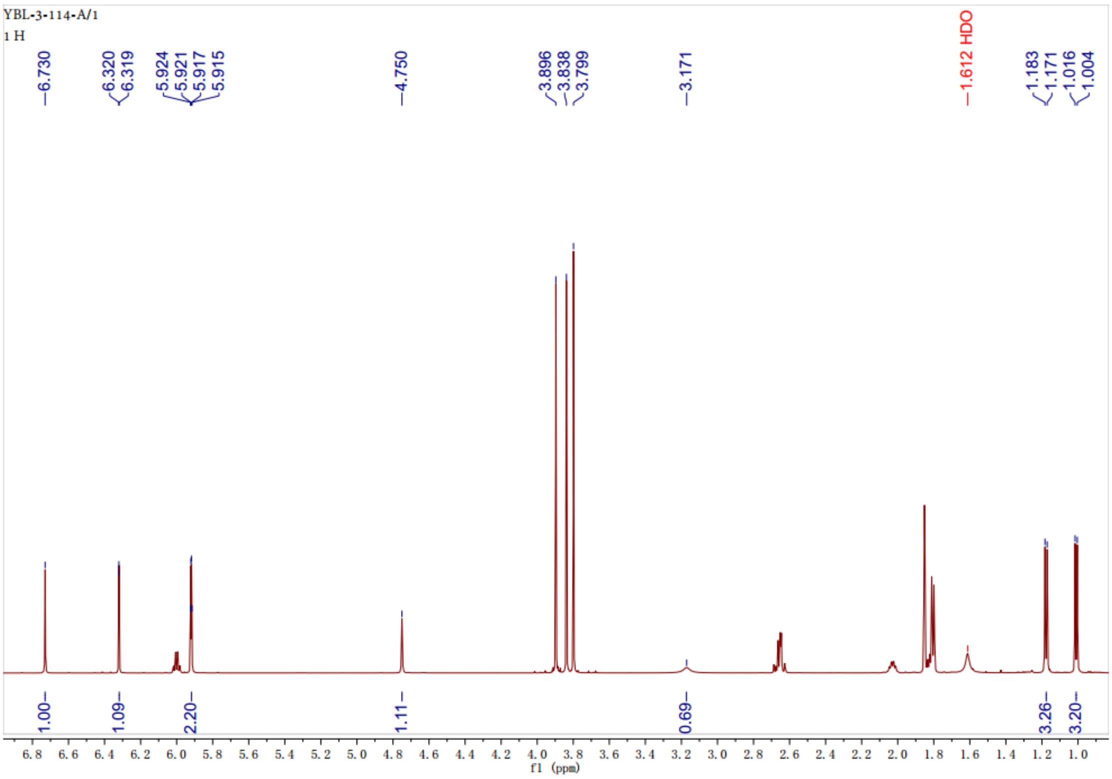

Figure S31. ^{13}C NMR (150 MHz, $CDCl_3$) spectrum of kadsuralignan I (**9**)

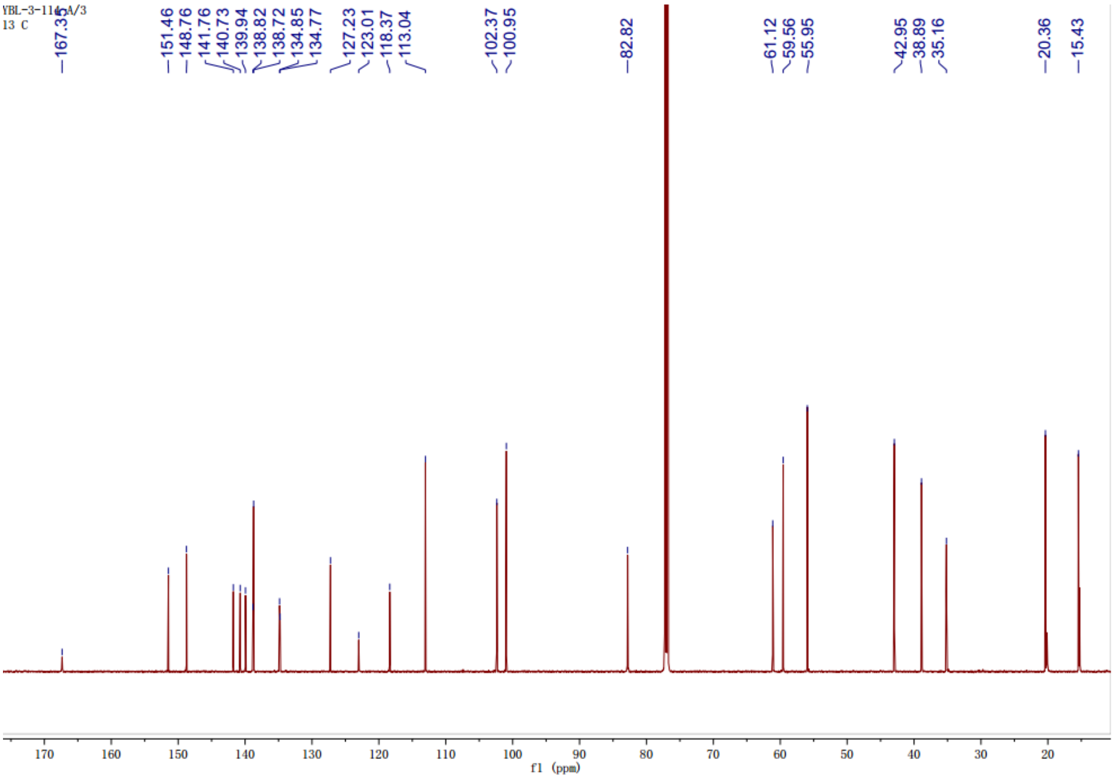

Figure S32. [1]**H NMR (600 MHz,** $CDCl_3$**) spectrum of kadsuralignan J (10)**

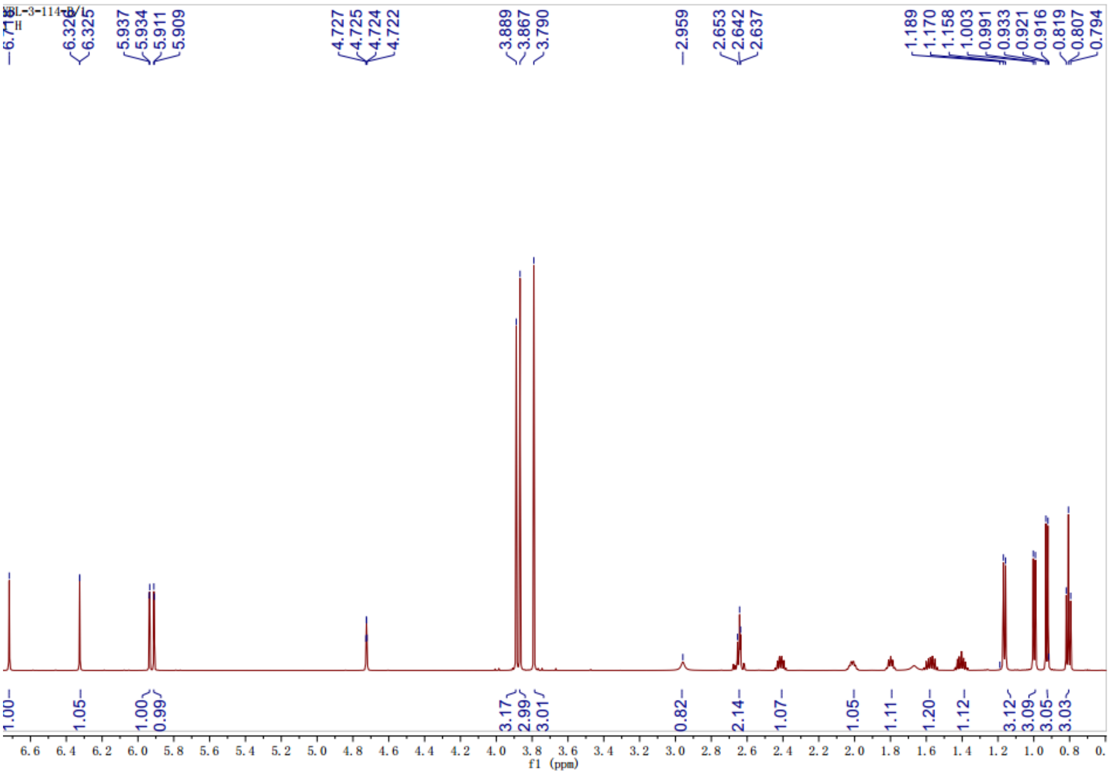

Figure S33. ^{13}C NMR (150 MHz, $CDCl_3$) spectrum of kadsuralignan J (**10**)

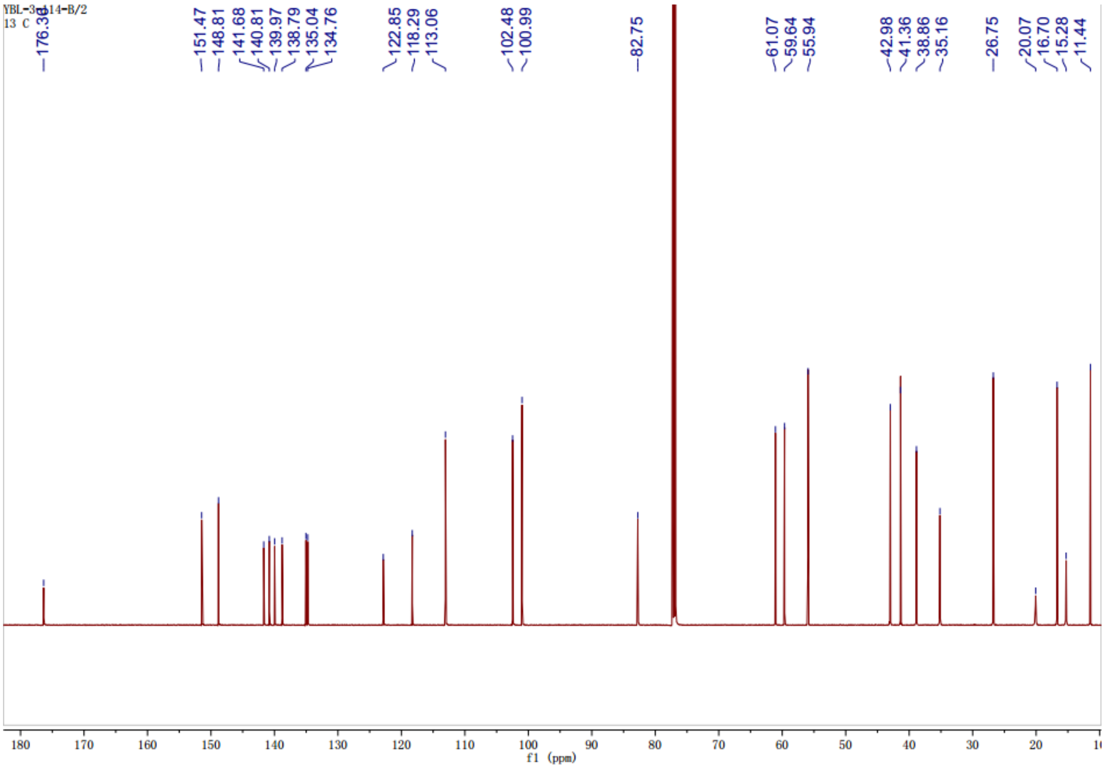

Figure S34. ^{1}H NMR (600 MHz, $CDCl_3$) spectrum of kadsuralignan L (11)

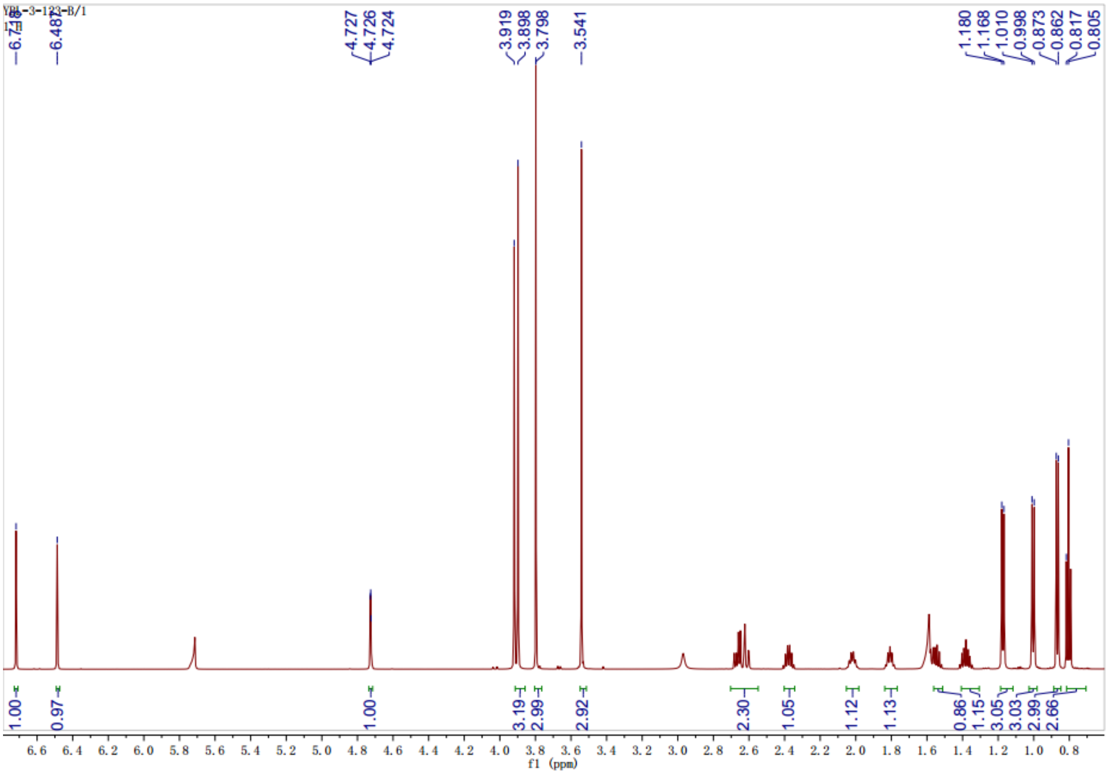

Figure S35. ^{13}C NMR (150 MHz, $CDCl_3$) spectrum of kadsuralignan L (11)

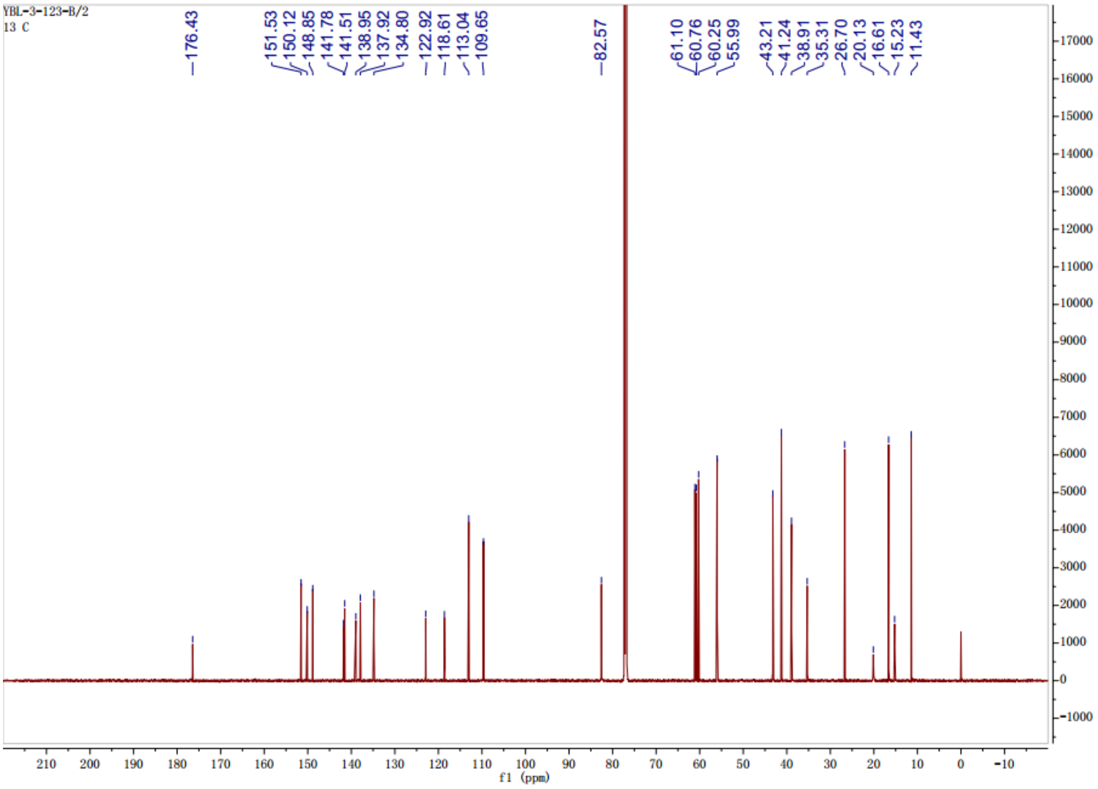

Figure S36. ^{1}H NMR (600 MHz, $CDCl_3$) spectrum of kadsulignan N (**12**)

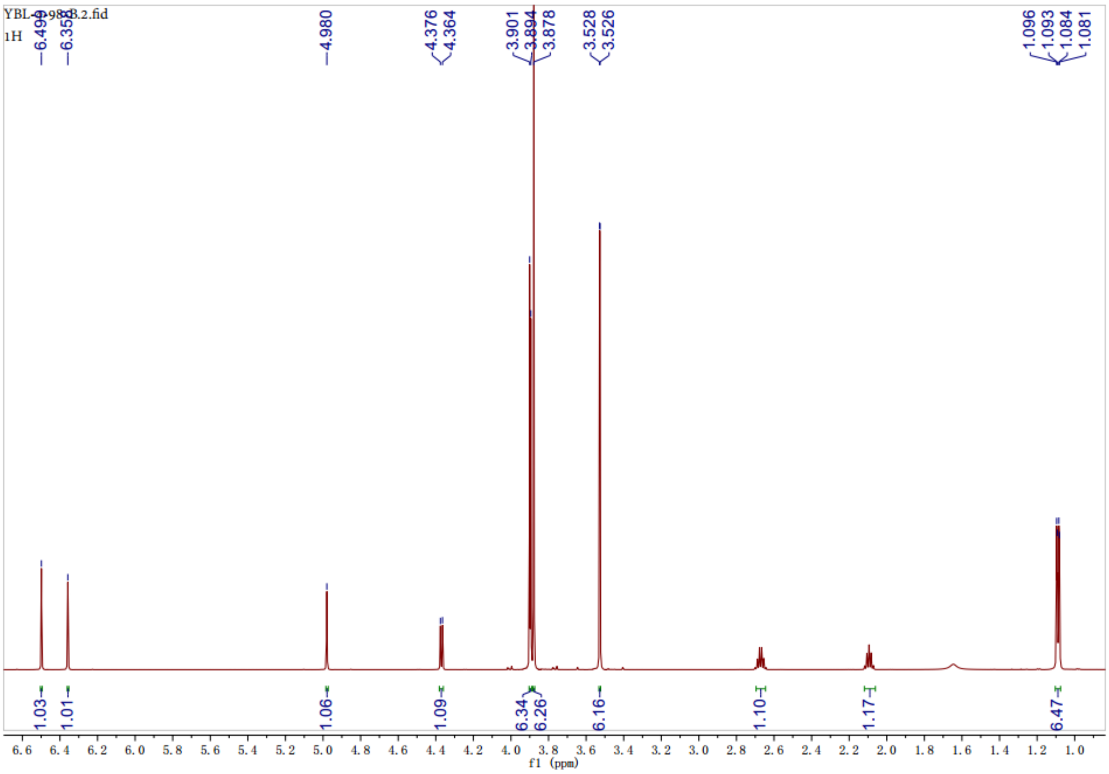

Figure S37. ^{13}C NMR (150 MHz, $CDCl_3$) spectrum of kadsulignan N (**12**)

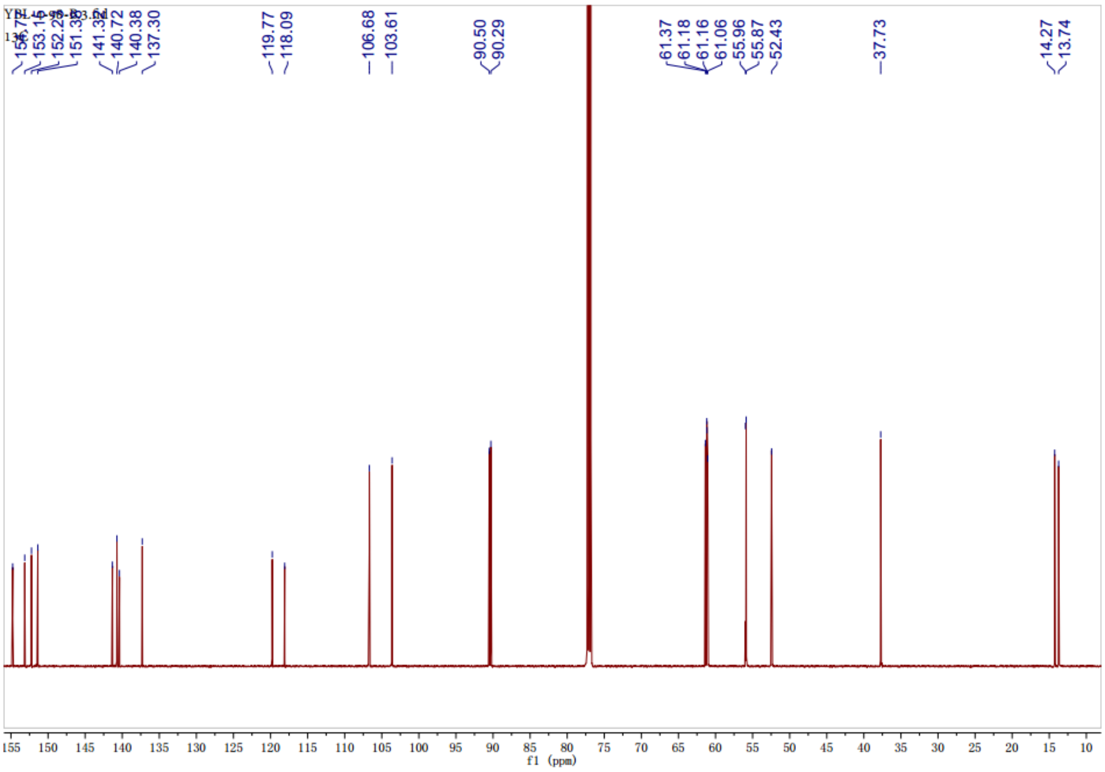

Figure S38. ^{1}H NMR (600 MHz, $CDCl_3$) spectrum of longipedunin B (**13**)

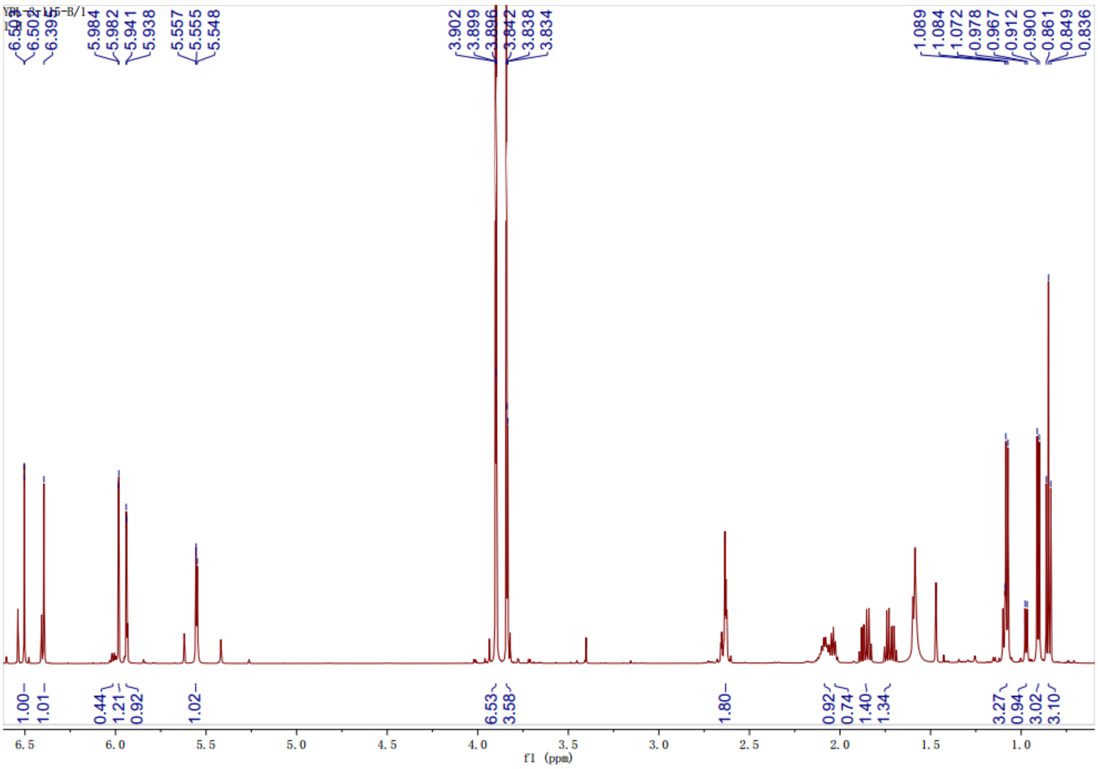

Figure S39. ^{13}C NMR (150 MHz, $CDCl_3$) spectrum of longipedunin B (**13**)

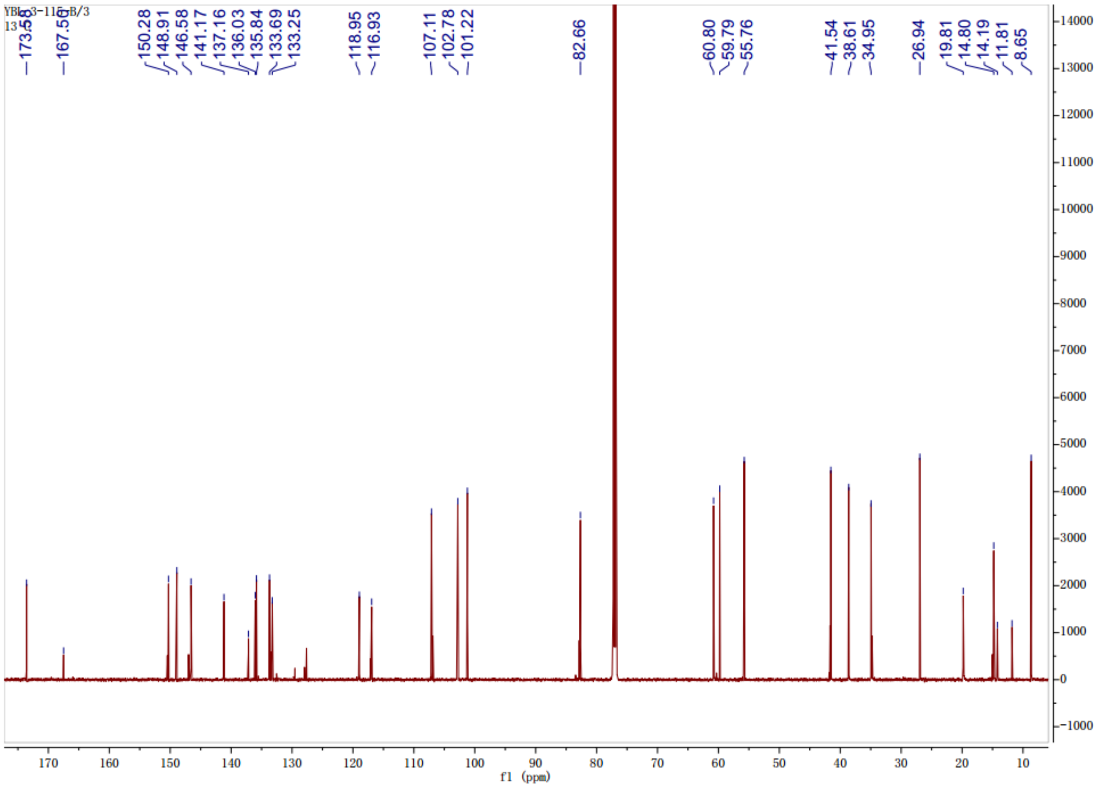

Figure S40. ^{1}H NMR (600 MHz, $CDCl_3$) spectrum of schisantherin F (**14**)

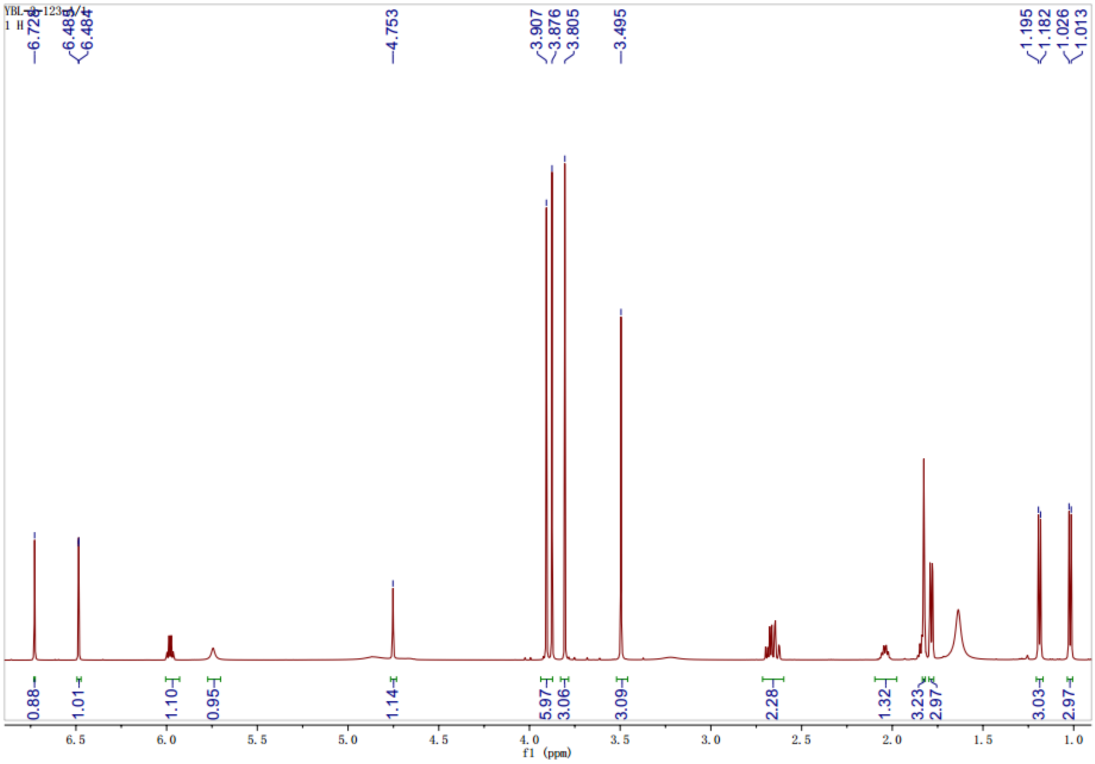

Figure S41. ^{13}C NMR (150 MHz, $CDCl_3$) spectrum of schisantherin F (**14**)

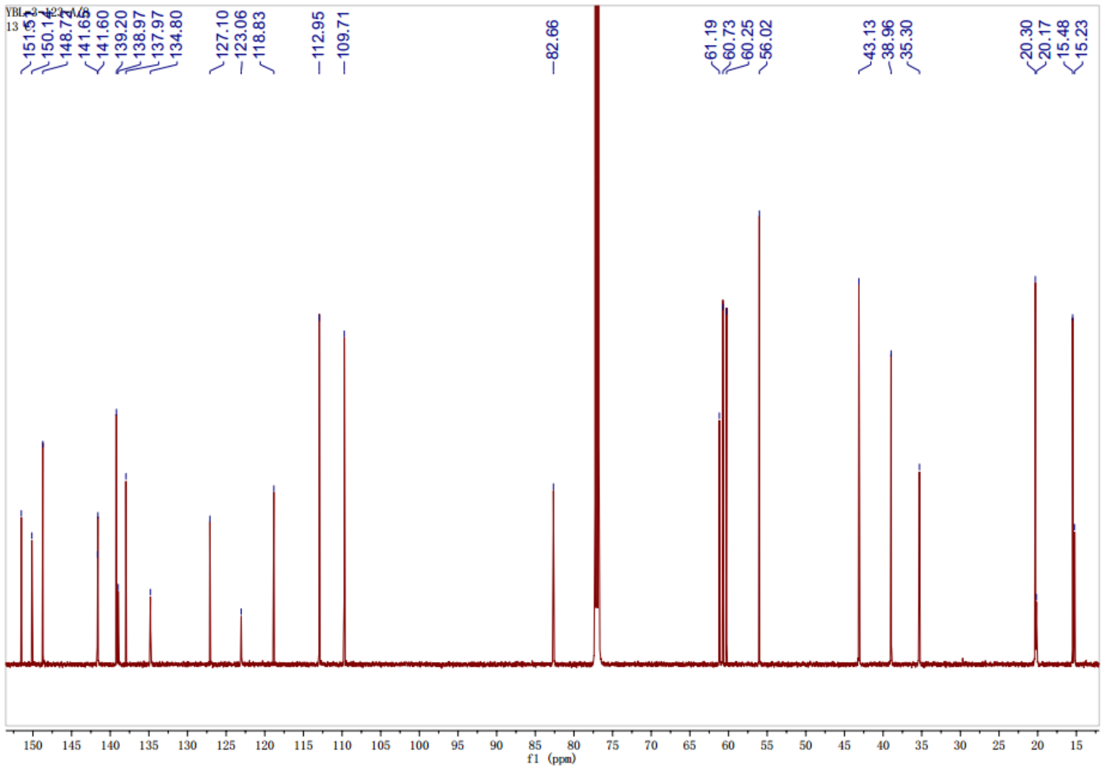

Figure S42. ^{1}H NMR (600 MHz, MeOD) spectrum of schizanrin D (**15**)

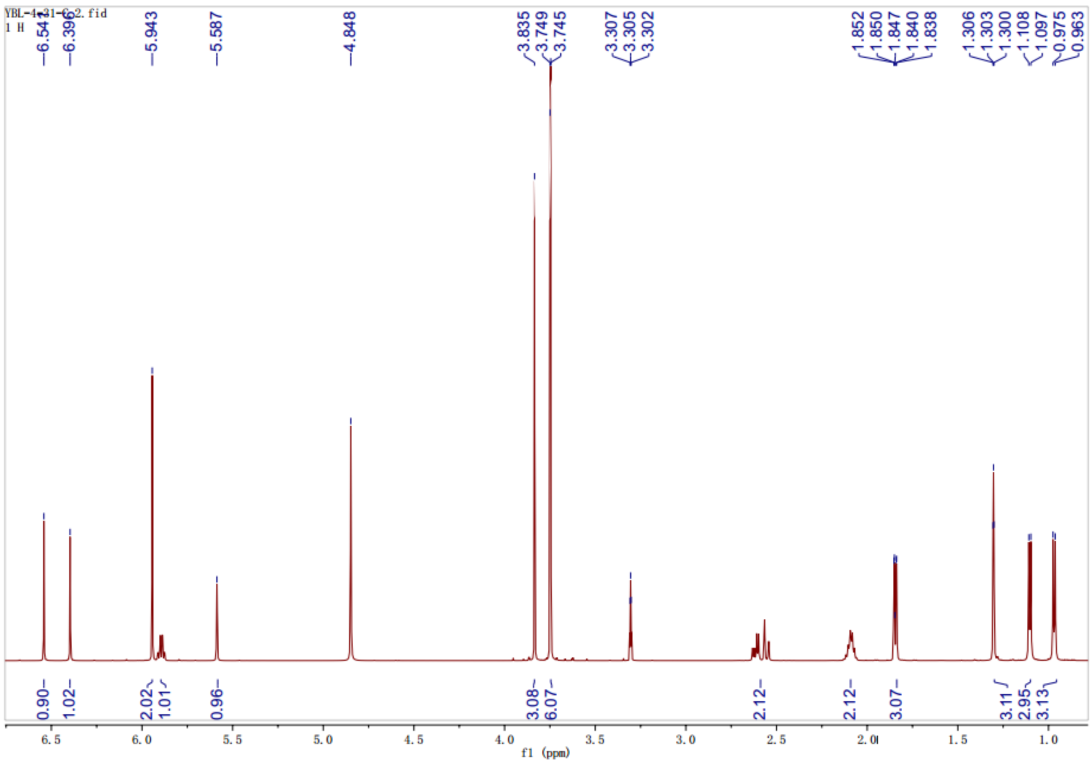

Figure S43. ^{13}C NMR (150 MHz, MeOD) spectrum of schizanrin D (**15**)

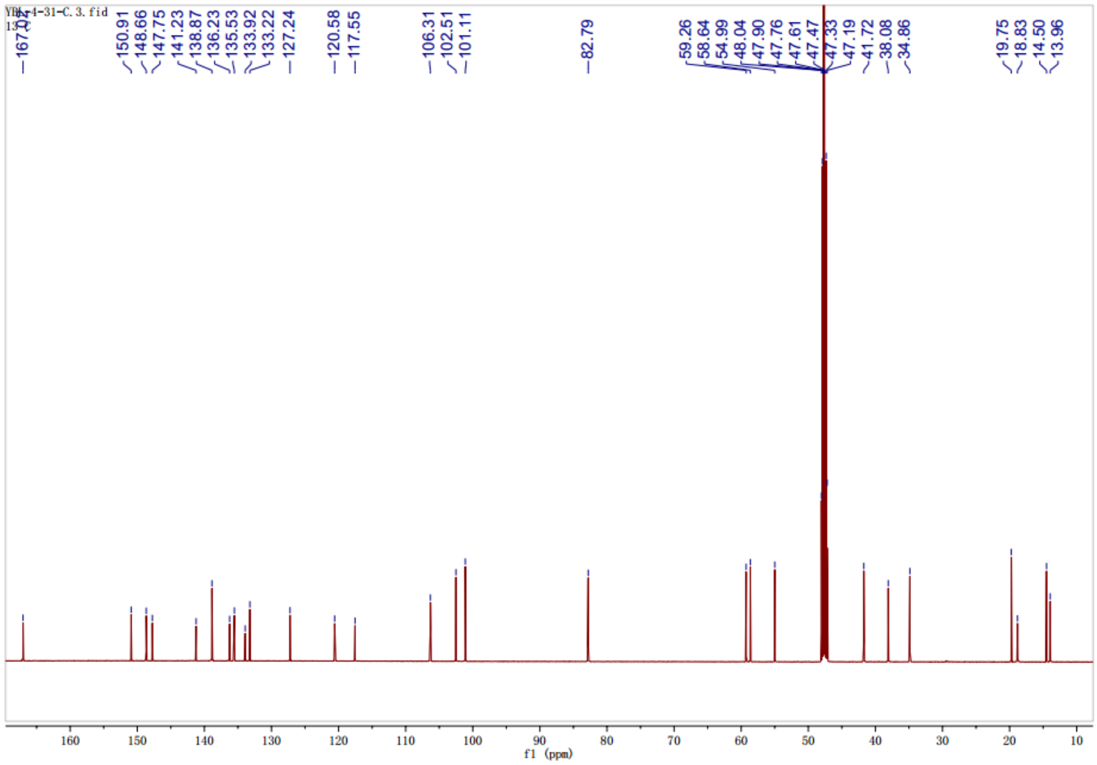

Figure S44. ^{1}H NMR (600 MHz, MeOD) spectrum of acetylgomisin R (**16**)

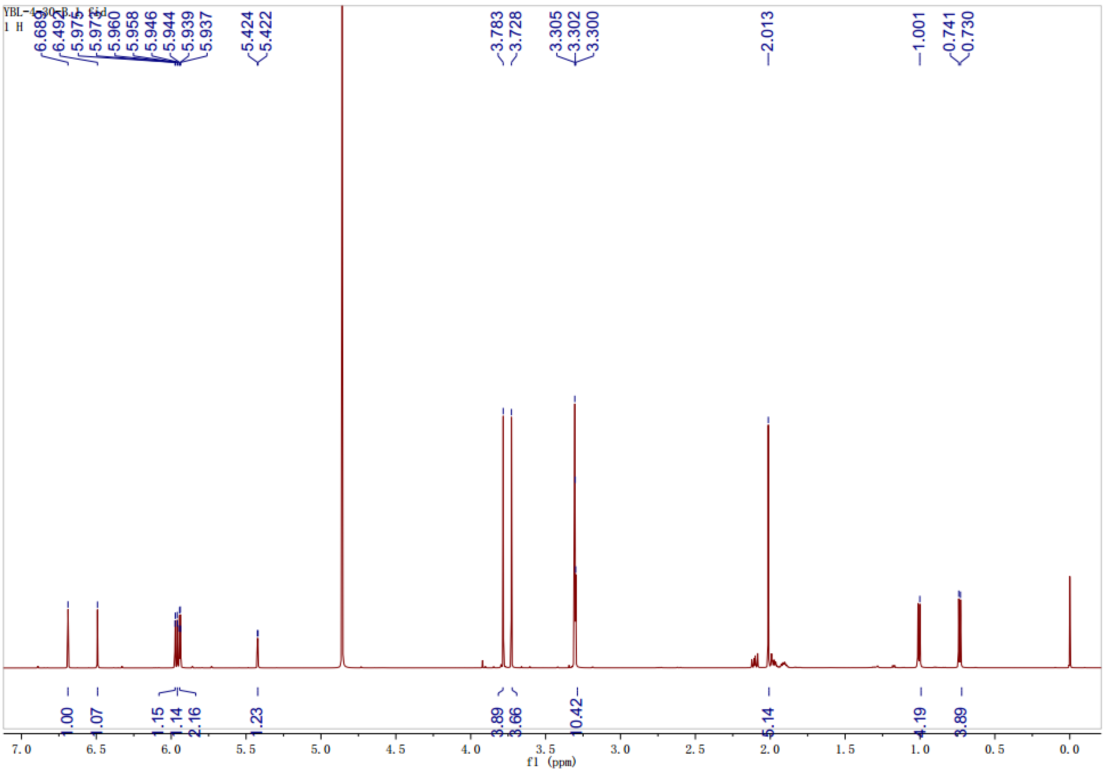

Figure S45. ^{13}C NMR (150 MHz, MeOD) spectrum of acetylgomisin R (**16**)

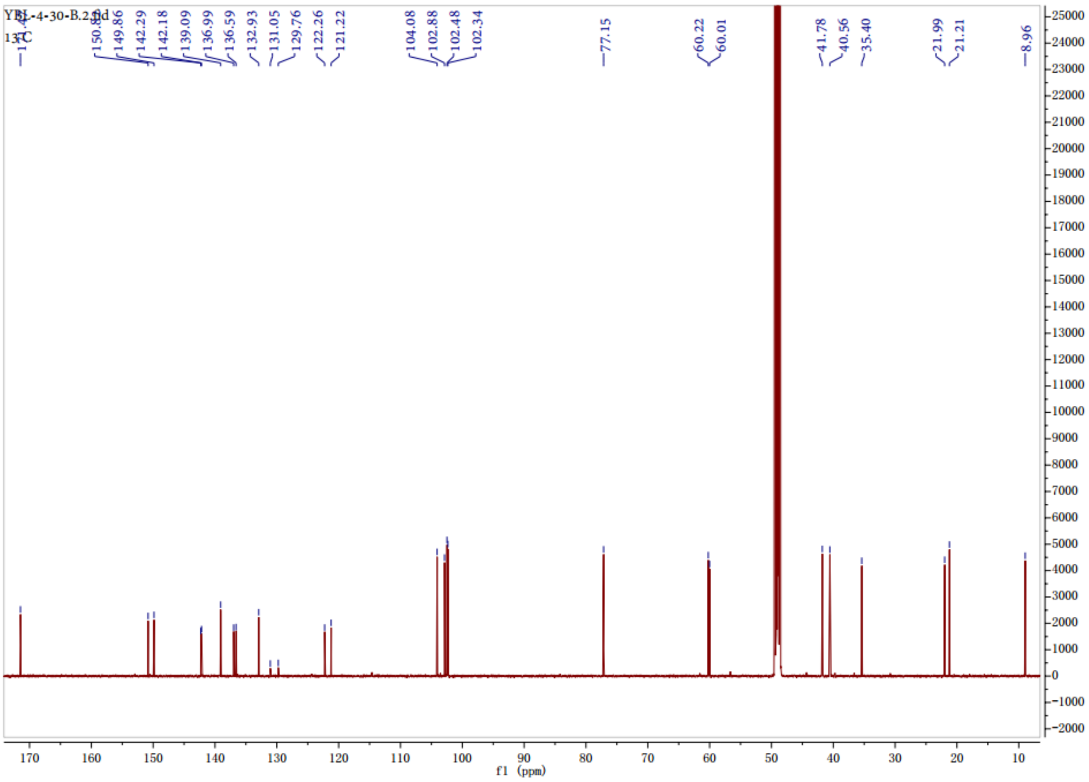

Figure S46. ^{1}H NMR (600 MHz, $CDCl_3$) spectrum of intermedin A (**17**)

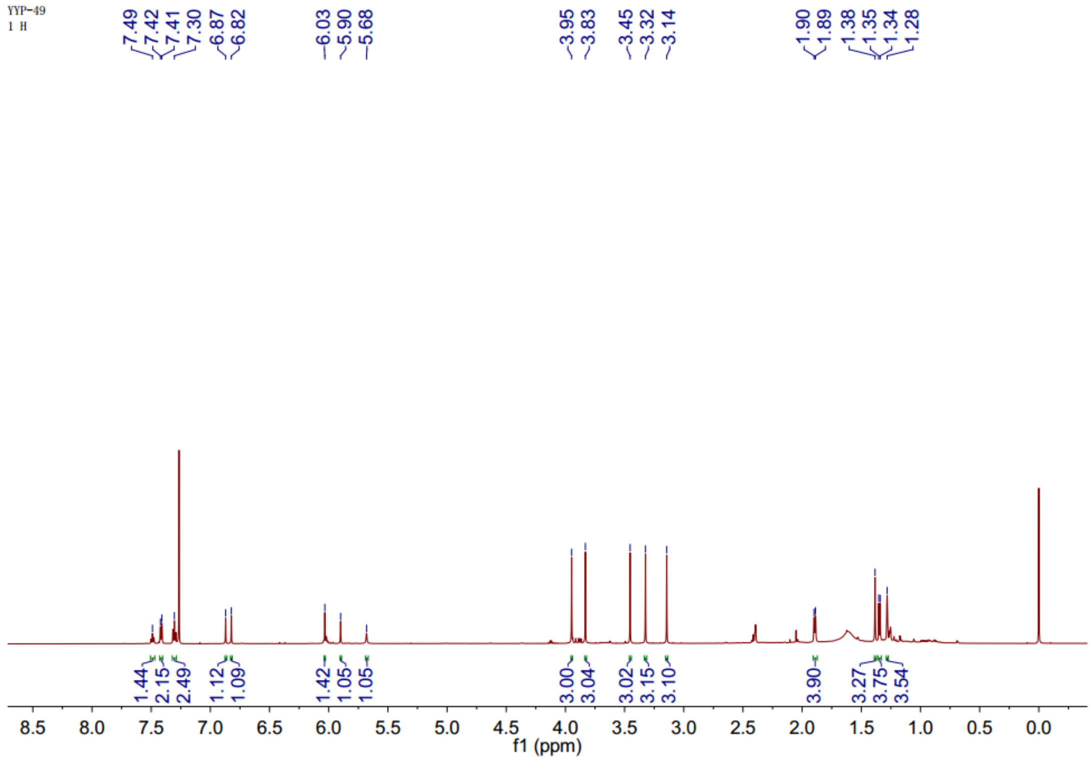

Figure S47. ^{13}C NMR (150 MHz, $CDCl_3$) spectrum of intermedin A (**17**)

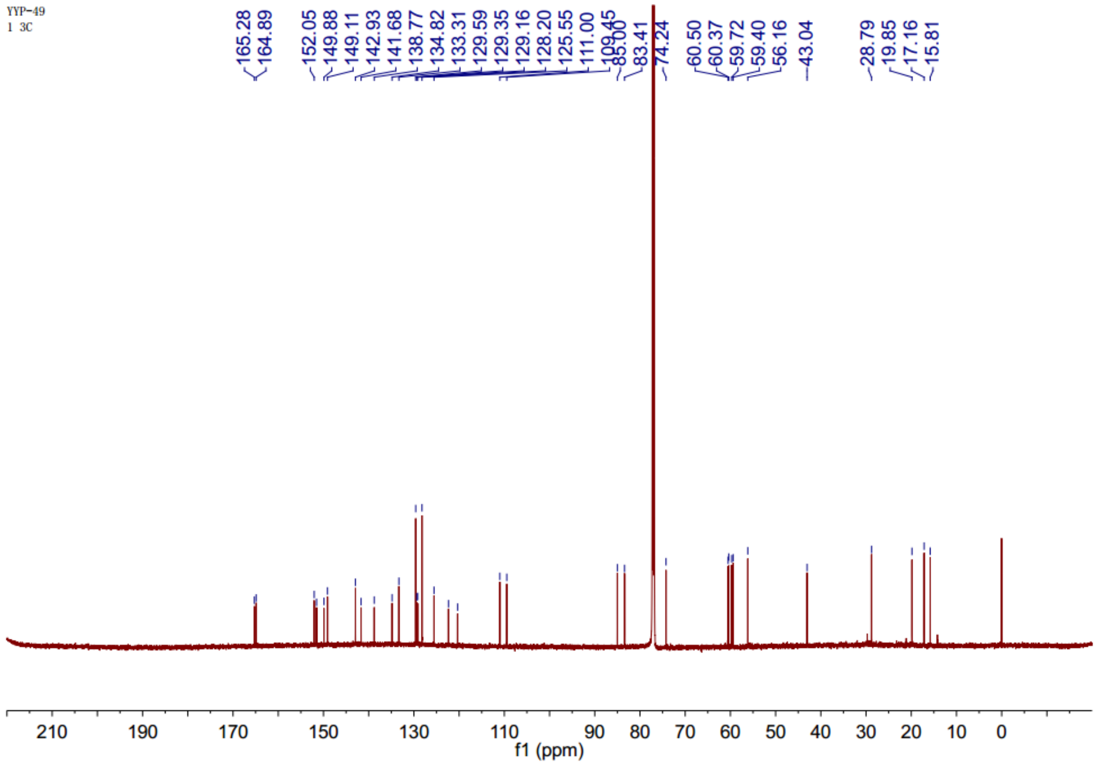

Figure S48. ^{1}H NMR (600 MHz, $CDCl_3$) spectrum of kadsurarin (**18**)

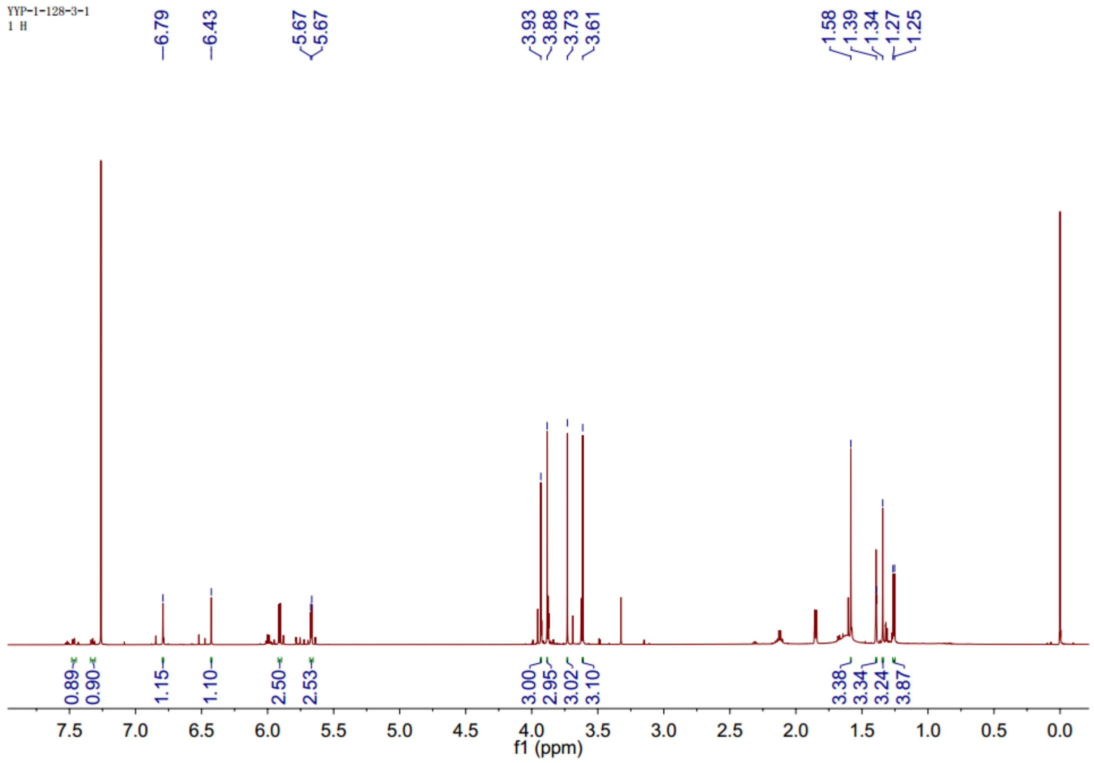

Figure S49. ^{13}C NMR (150 MHz, $CDCl_3$) spectrum of kadsurarin (**18**)

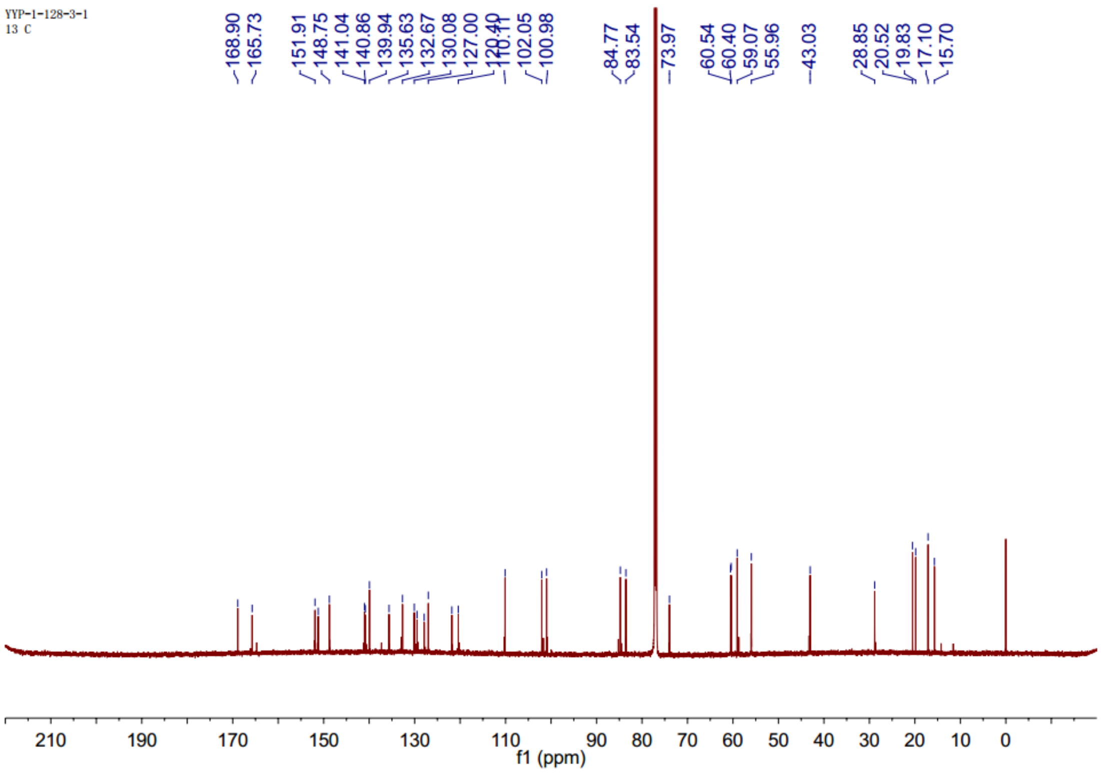

Figure S50. ^{1}H NMR (600 MHz, $CDCl_3$) spectrum of kadsutherin A (**19**)

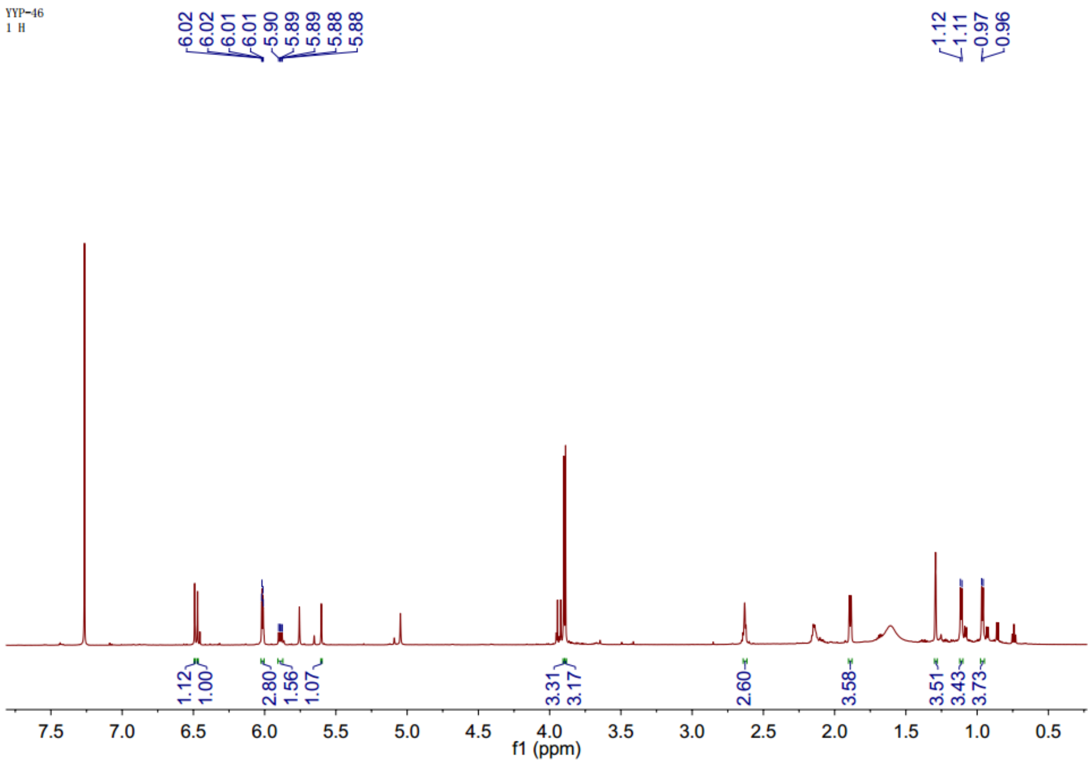

Figure S51. ^{13}C NMR (150 MHz, $CDCl_3$) spectrum of kadsutherin A (**19**)

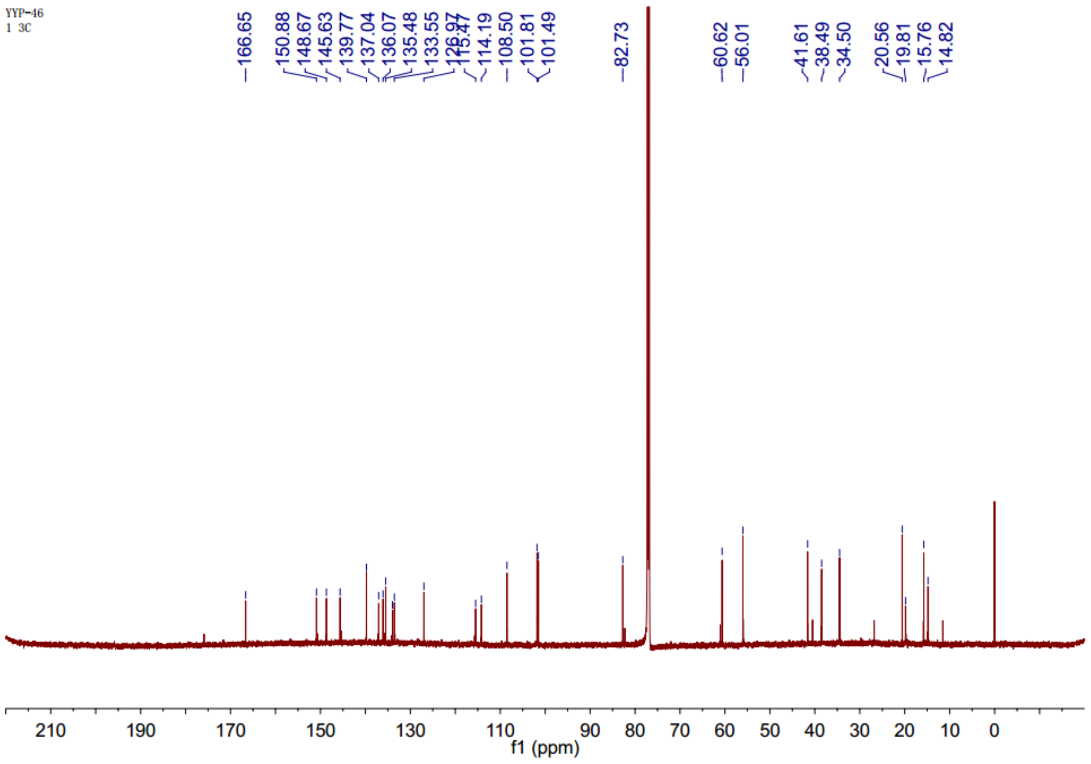

Figure S52. ^{1}H NMR (600 MHz, $CDCl_3$) spectrum of kadsuphilol A (**20**)

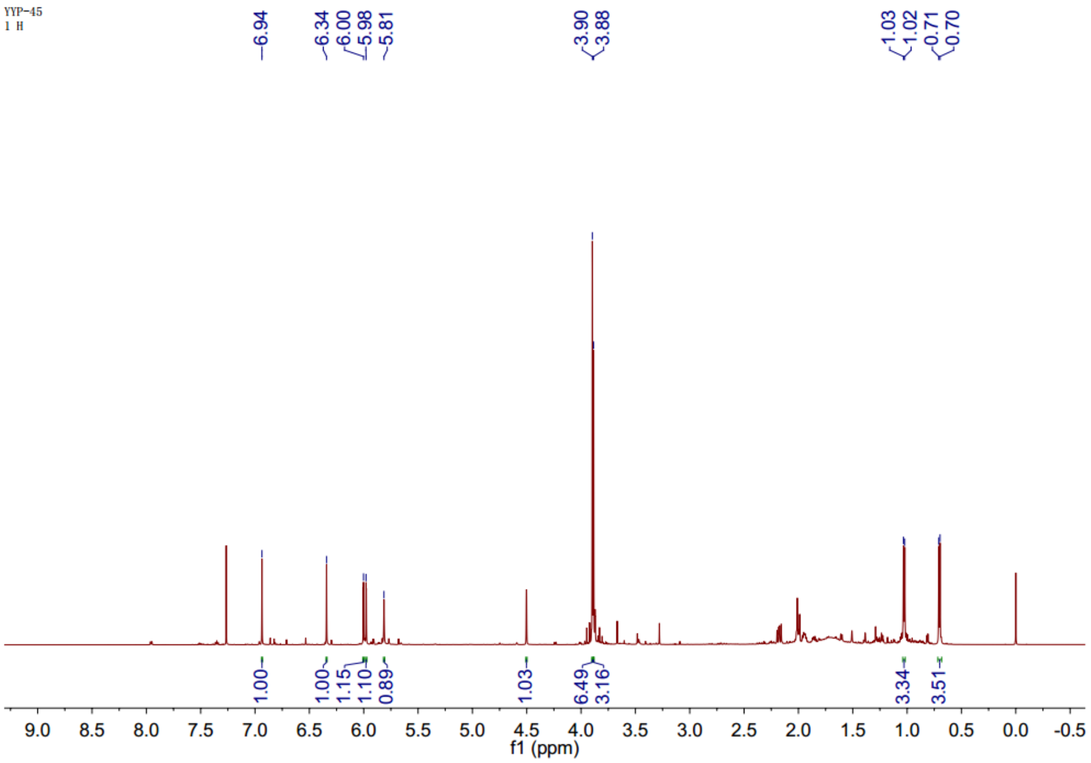

Figure S53. ^{13}C NMR (150 MHz, $CDCl_3$) spectrum of kadsuphilol A (**20**)

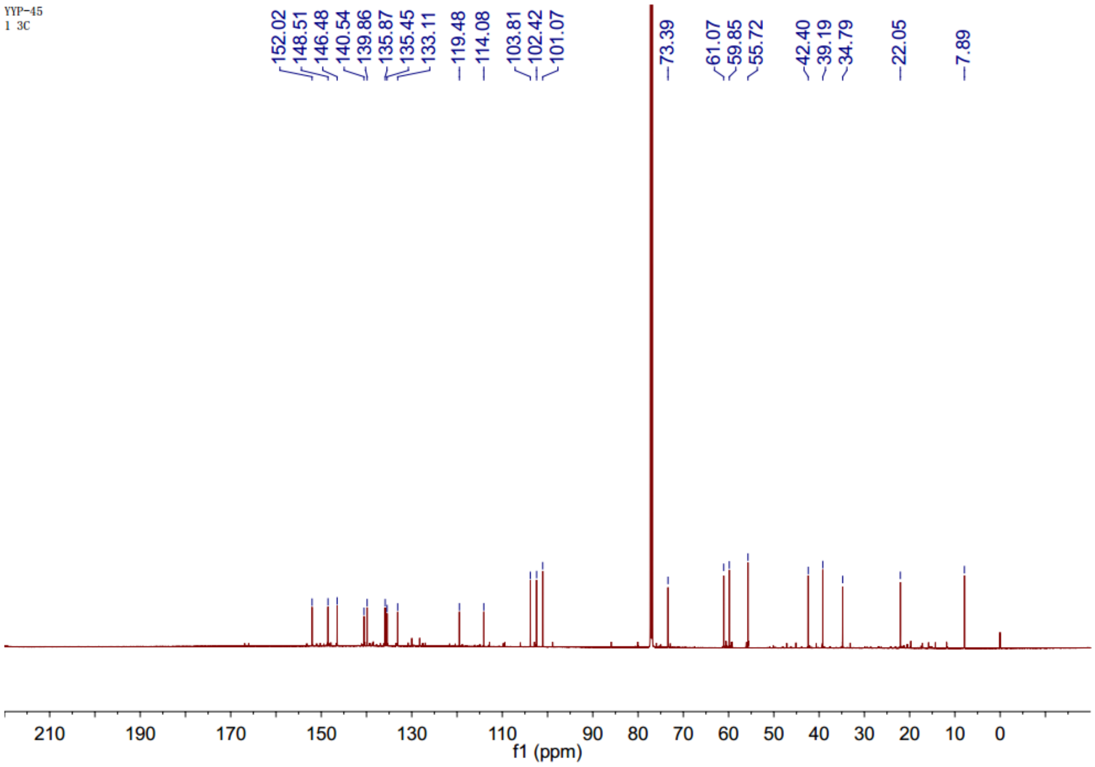

Figure S54. ^{1}H NMR (600 MHz, $CDCl_3$) spectrum of meso-dihydroguaiaretic acid dimethyl (**21**)

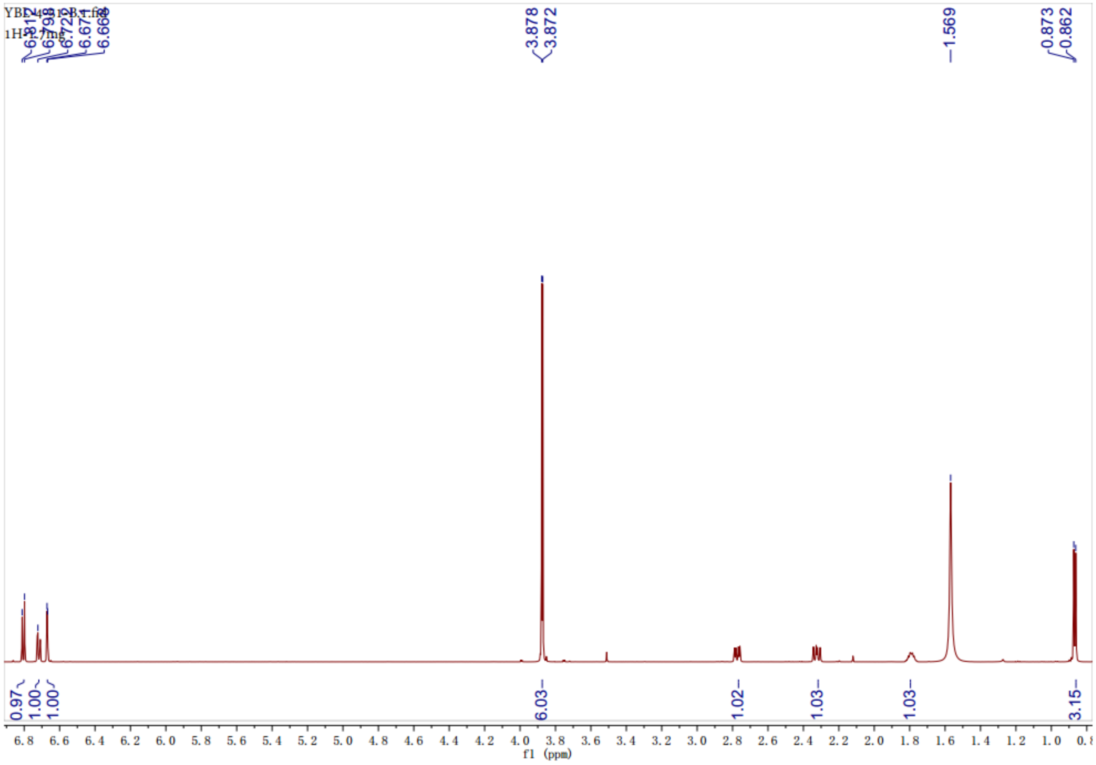

Figure S55. ^{13}C NMR (150 MHz, $CDCl_3$) spectrum of meso-dihydroguaiaretic acid dimethyl (**21**)

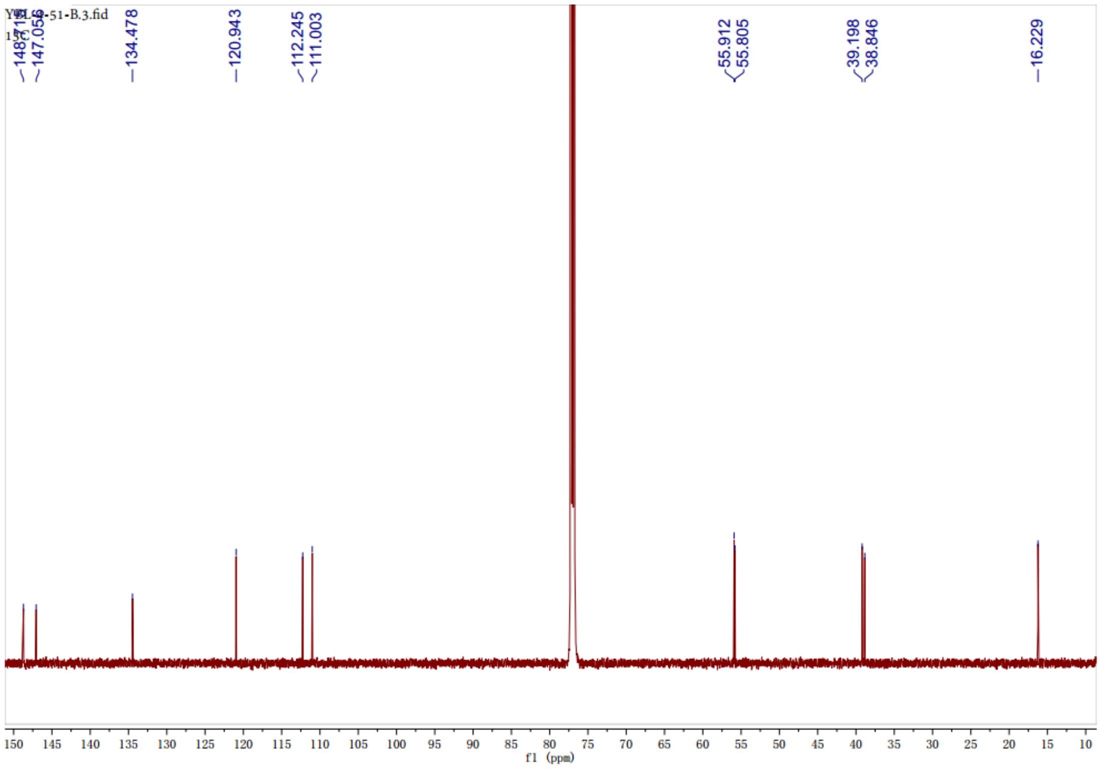

Figure S56. ^{1}H NMR (600 MHz, $CDCl_3$) spectrum of schiarianrin E (**22**)

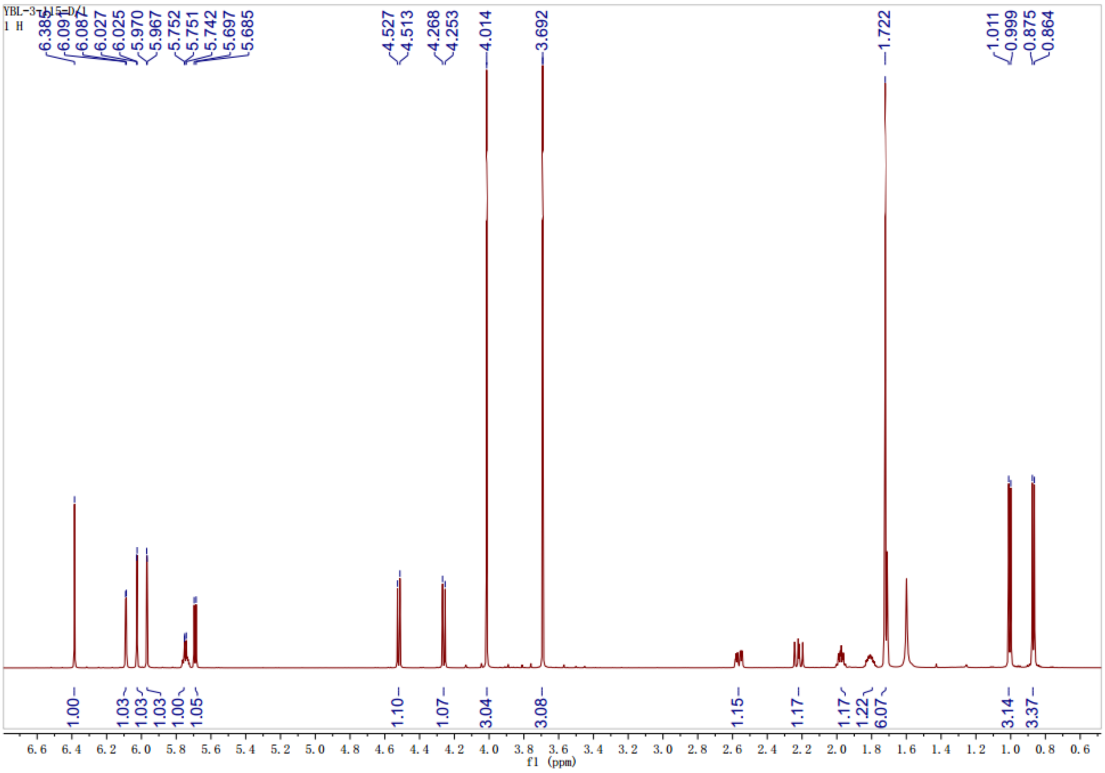

Figure S57. ^{13}C NMR (150 MHz, $CDCl_3$) spectrum of schiarianrin E (**22**)

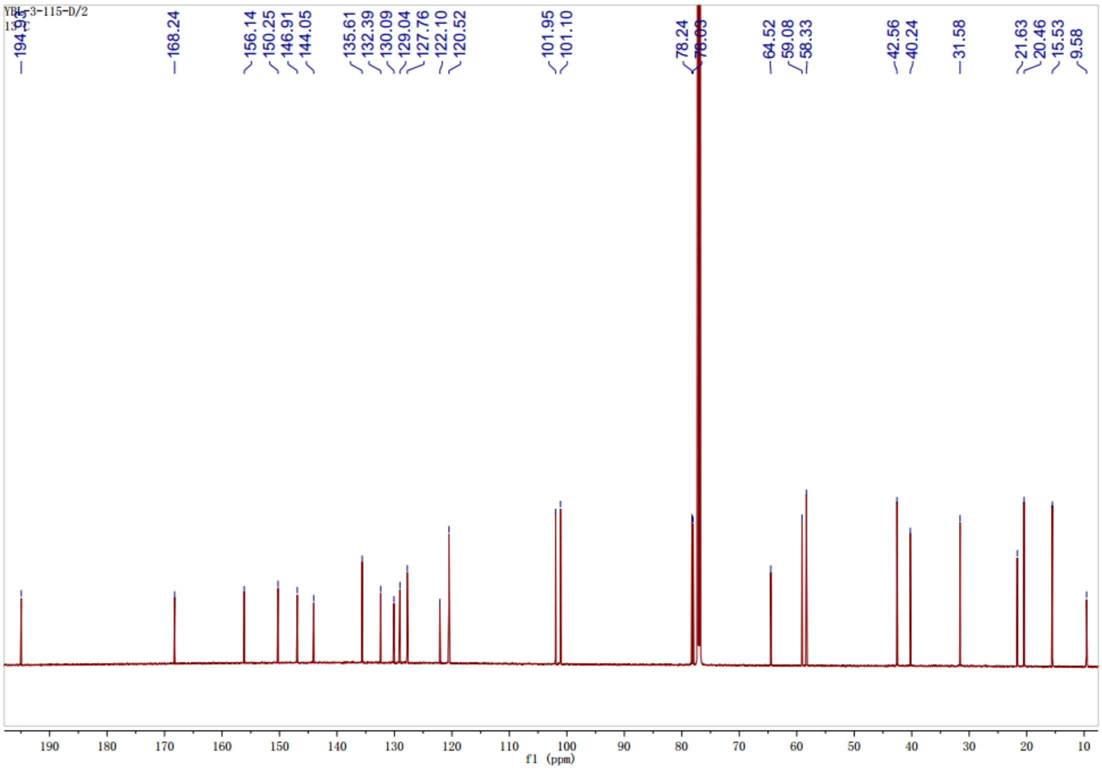

Figure S58. ^{1}H NMR (600 MHz, $CDCl_3$) spectrum of schiarisanrin A (**23**)

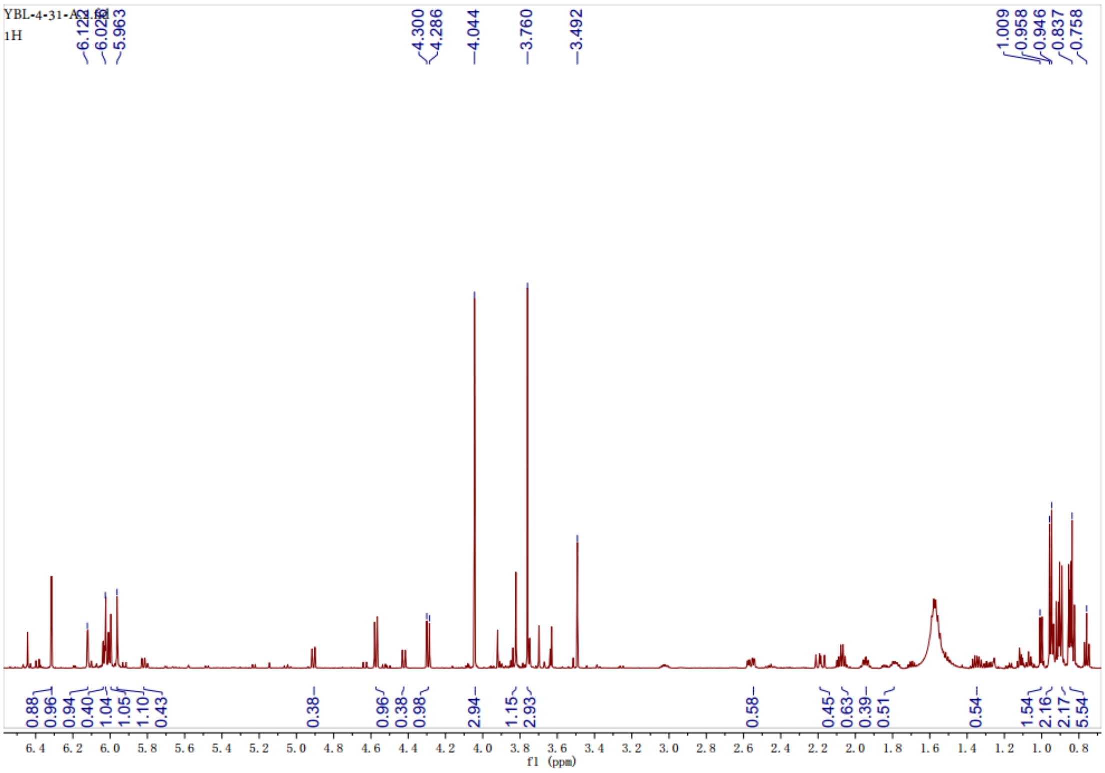

Figure S59. ^{13}C NMR (150 MHz, $CDCl_3$) spectrum of schiarisanrin A (**23**)

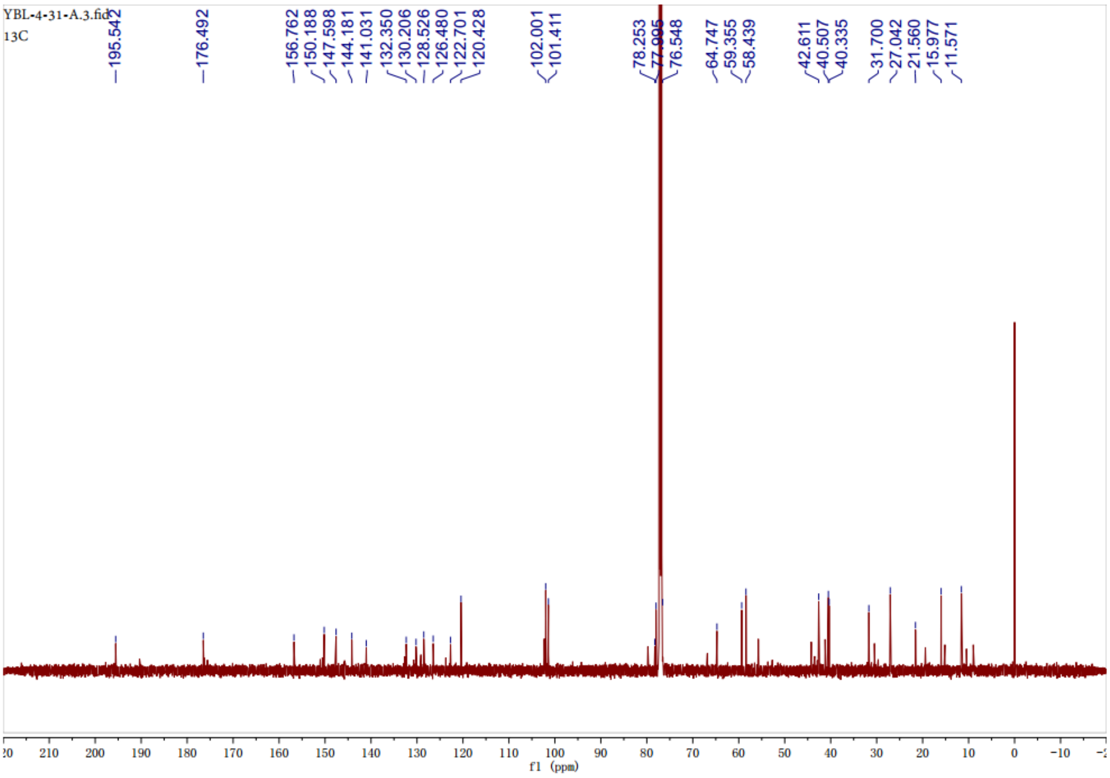

Figure S60. HRESIMS spectrum of heilaohulignan A (**1**)

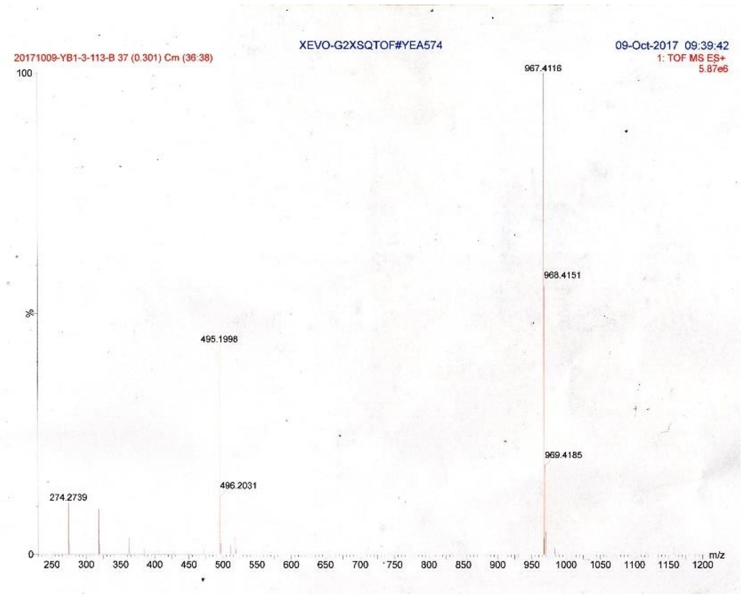

Figure S61. HRESIMS spectrum of heilaohulignan B (**2**)

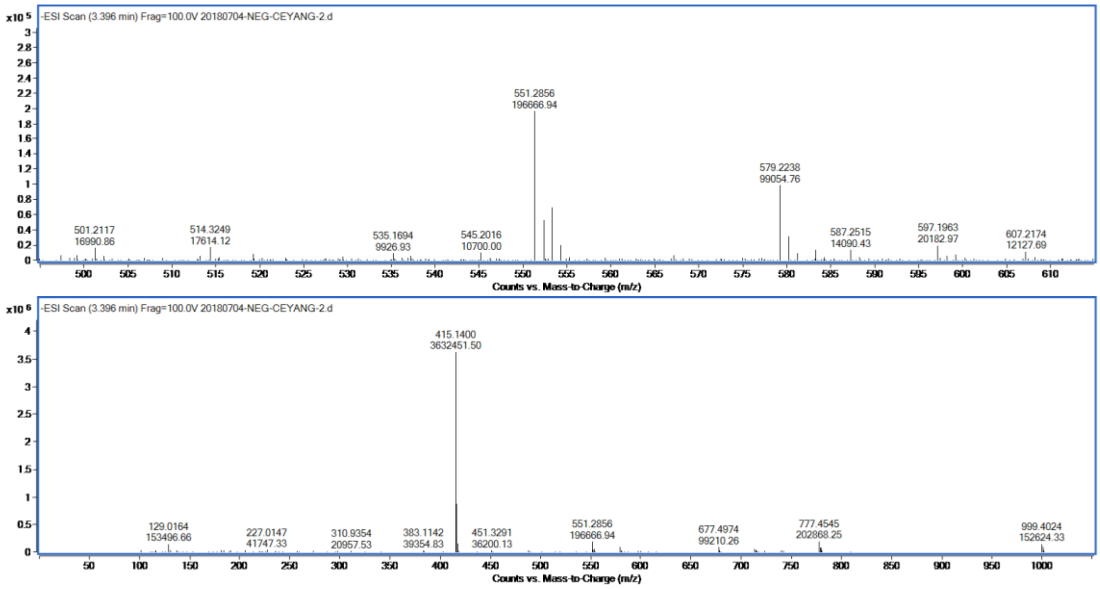

Figure S62. HRESIMS spectrum of heilaohulignan C (**3**)

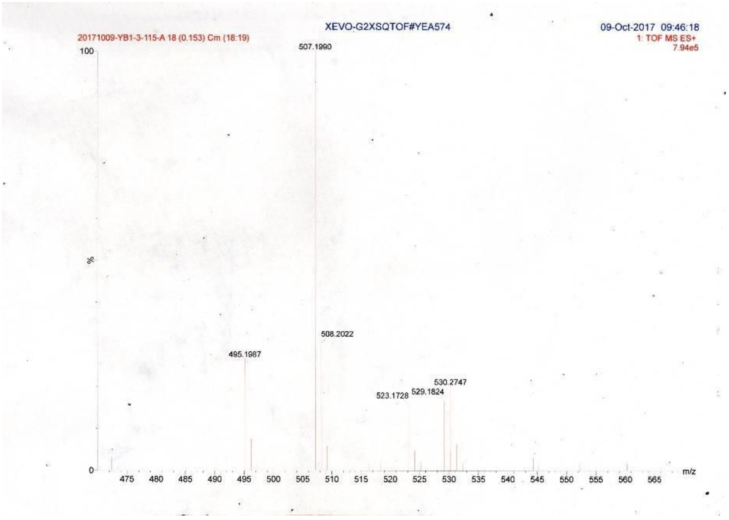

Figure S63. CD spectrum of heilaohulignan A (**1**)

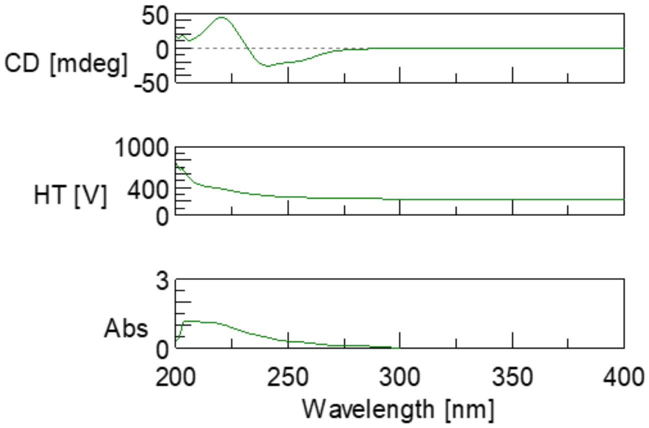

Figure S64. CD spectrum of heilaohulignan B (**2**)

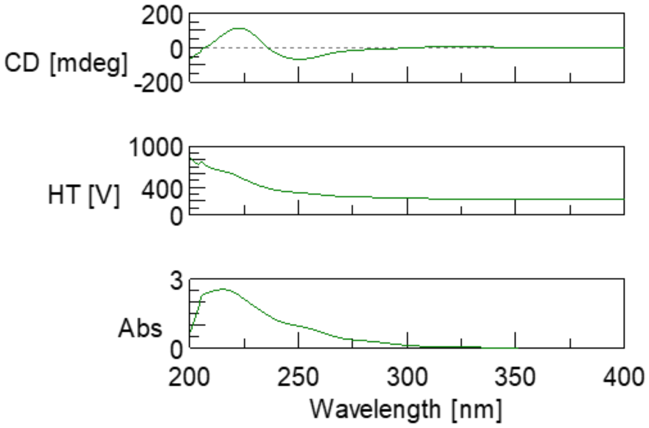

Figure S65. CD spectrum of heilaohulignan C (**3**)

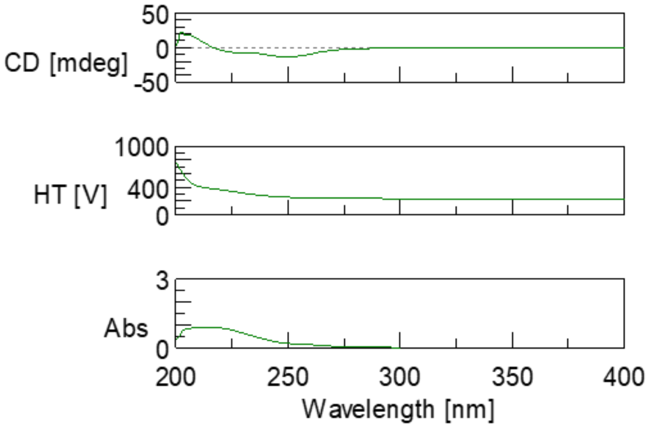

Supplement: Supplementary file 1 [file molecules-23-02147-s001.zip › molecules-342961-SI.pdf]
